# Telomere sequence variability in genotypes from natural plant populations: unusual block-organized double-monomer terminal telomeric arrays

**DOI:** 10.1186/s12864-023-09657-y

**Published:** 2023-09-26

**Authors:** Alexander Belyayev, Ruslan Kalendar, Jiřina Josefiová, Ladislava Paštová, Farzaneh Habibi, Václav Mahelka, Bohumil Mandák, Karol Krak

**Affiliations:** 1 Institute of Botany, Czech Academy of Sciences, Zámek 1, CZ-252 43, Průhonice, Czech Republic; 2 Institute of Biotechnology HiLIFE, University of Helsinki, P.O. Box 65, Helsinki, FI-00014 Finland; 3 National Laboratory Astana, Nazarbayev University, 53 Kabanbay Batyr Ave, Nur- Sultan, 010000 Kazakhstan; 4 Faculty of Environmental Sciences, Czech University of Life Sciences Prague, Kamýcká 129, Praha, Suchdol, 165 00 Czech Republic

**Keywords:** Evolution, Oxford nanopore sequencing, Plant, Population, Species, Telomere

## Abstract

**Background:**

Telomeres are the nucleoprotein complexes that physically cap the ends of eukaryotic chromosomes. Most plants possess *Arabidopsis*-type telomere sequences (TSs). In addition to terminal TSs, more diverse interstitial TSs exists in plants. Although telomeres have been sufficiently studied, the actual diversity of TSs in land plants is underestimated.

**Results:**

We investigate genotypes from seven natural populations with contrasting environments of four *Chenopodium* species to reveal the variability in TSs by analyzing Oxford Nanopore reads. Fluorescent in situ hybridization was used to localize telomeric repeats on chromosomes. We identified a number of derivative monomers that arise in part of both terminal and interstitial telomeric arrays of a single genotype. The former presents a case of block-organized double-monomer telomers, where blocks of *Arabidopsis*-type TTTAGGG motifs were interspersed with blocks of derivative TTTAAAA motifs. The latter is an integral part of the satellitome with transformations specific to the inactive genome fraction.

**Conclusions:**

We suggested two alternative models for the possible formation of derivative monomers from telomeric heptamer motifs of *Arabidopsis*-type. It was assumed that derivatization of TSs is a ubiquitous process in the plant genome but occurrence and frequencies of derivatives may be genotype-specific. We also propose that the formation of non-canonical arrays of TSs, especially at chromosomal termini, may be a source for genomic variability in nature.

**Supplementary Information:**

The online version contains supplementary material available at 10.1186/s12864-023-09657-y.

## Background

Telomeres are the nucleoprotein complexes that physically cap and protect the ends of eukaryotic chromosomes [1, 2]. In most eukaryotes, telomeres are specialized chromosomal DNA composed of short tandem repeats. Telomeric repeats are ancient and conserved sequences: the repeats of all vertebrate telomeres are (TTAGGG)_n_, those typical for insects are (TTAGG)_n_, and those of most plants are the *Arabidopsis*-type telomere repeat (TTTAGGG)_n_. Thus, the monomers of these large taxa differ by only one to two nucleotides [3]. However, even a small change in the telomere motif appears to result in marked interference in the system of sequence-specific telomere binding proteins which coated telomere tract and act as a barrier against DNA repair machinery and exonucleolytic degradation [2, 3]. In this regard, any variations in the structure of telomeric monomers are evolutionarily significant [4, 5]. In land plants, in addition to the dominant *Arabidopsis*-type motif [6], the vertebrate-type motif (TTAGGG)_n_ was found in Asparagales [7], and different variants of telomere motifs were found in several Asterid species (reviewed in [3]) That proposed to result from telomerase RNA paralogs whose template regions could support the synthesis of diverse telomeres [8]. Although variations in the monomers were observed, the terminal telomeric arrays were determined to be homogeneous.

In addition to terminal TSs, interstitial telomeric repeats (ITRs) were identified in several plant species [6, 9–13]. ITRs are more diverse in monomers and structure than terminal TSs due to many degenerative units with base substitutions or small indels [14]. ITRs might have originated from ancestral chromosomal rearrangements involving chromosome ends, from differential crossing-over, or from the evolutionary remnants of double-strand break repair events [15]. Therefore, the large number and diversity of ITRs may point to intensive genome rearrangements during key evolutionary events such as speciation, for example.

Notwithstanding that the structural and functional features of the telomeric regions of chromosomes have been sufficiently studied, especially in model species, the real diversity of TSs and especially ITRs in land plants is proposed to be greatly underestimated [3]. Thus, we sought to investigate the genomes of *Chenopodium* (*Amaranthaceae*, *Caryophyllales*) species from natural populations to uncover the structural variability of TSs and to evaluate their role in satellitome composition. *Caryophyllales* is a separate branch of Angiosperms and a member of the “Superasterids” at the base of the monophyletic “Asterids” group [5]. The genomes of four diploid species (2n = 2x = 18), *C. acuminatum*, *C. iljinii*, *C. pamiricum*, and *C. suecicum*, which belong to different *Chenopodium* branches [16], have been investigated (Table 1). The species selected for the study inhabit different environments. *C. acuminatum* is a continental steppe species with a central distribution range in Mongolia and surrounding countries. *C. suecicum* grows in the Eurasian boreal zone. Closely related *C. iljinii* and *C. pamiricum* are of special interest because of their sympatric distribution with ecological separation. *C. pamiricum* grows on stony, gravely, or desert steppes, on clay patches at high altitudes (2000–5000 m) of the Hindu Kush, the northwestern Himalayas, Pamir, Tian-Shan, and Tibet and at lower altitudes farther north, in the Altai and Sayan mountains, and in mountain ranges of Mongolia up to 2200 m. *C. iljinii* is a steppe-semidesert species growing on stony and sandy slopes, on steppes, along roadsides and in fallow and waste lands at lower altitudes [16, 17]. For these species, we studied several accessions from locations with contrasting environments. Thus, we assume that current sampling should reveal any: (i) interspecific polymorphisms of TSs in the genomes of the four *Chenopodium* species, (ii) intraspecific polymorphisms of TSs in the genomes of *C. iljinii* and *C. pamiricum*, and (iii) the role of TSs in satellitome formation. In our research we analyzed Oxford Nanopore (ON) read libraries and used fluorescent in situ hybridization (FISH) to localize telomeric repeats on chromosomes and on DNA fibers.

**Table 1:** Accessions and geographic origins of analyzed diploid *Chenopodium* species*

Species	Genome	Accession #	Locality	Coordinates	Altitude
*C. acuminatum* Willd.	D	429-3	China, Xinjiang, Altai, Burqin	N 47.815500, E 87.080028	527 m
*C. iljinii* Golosk.	E	433-9	China, Xinjiang, Altai, Hoboksar	N 46.541472, E 85.358083	1118 m
		441-6	China, Xinjiang, Hoxud, Bo Si’amu	N 42.474417, E 86.877806	1932 m
		461-5	China, Xinjiang, Tumuxiukezhen	N 41.667306, E 79.693528	2202 m
*C. pamiricum* Iljin	E	177	Russia, Altai, Kosh-Agach	N 50.055278, E 88.708611	1939 m
		830-3 C	Tajikistan, Gorno-Badakhshan, Murghob	N 37.821667, E 73.566667	3937 m
*C. suecicum* Murr	B	328 − 10	Czech Republic, Švermov	N 50.176806, E 14.105472	349 m

*All species are not endangered

## Results and discussion

**Fig. 1:**
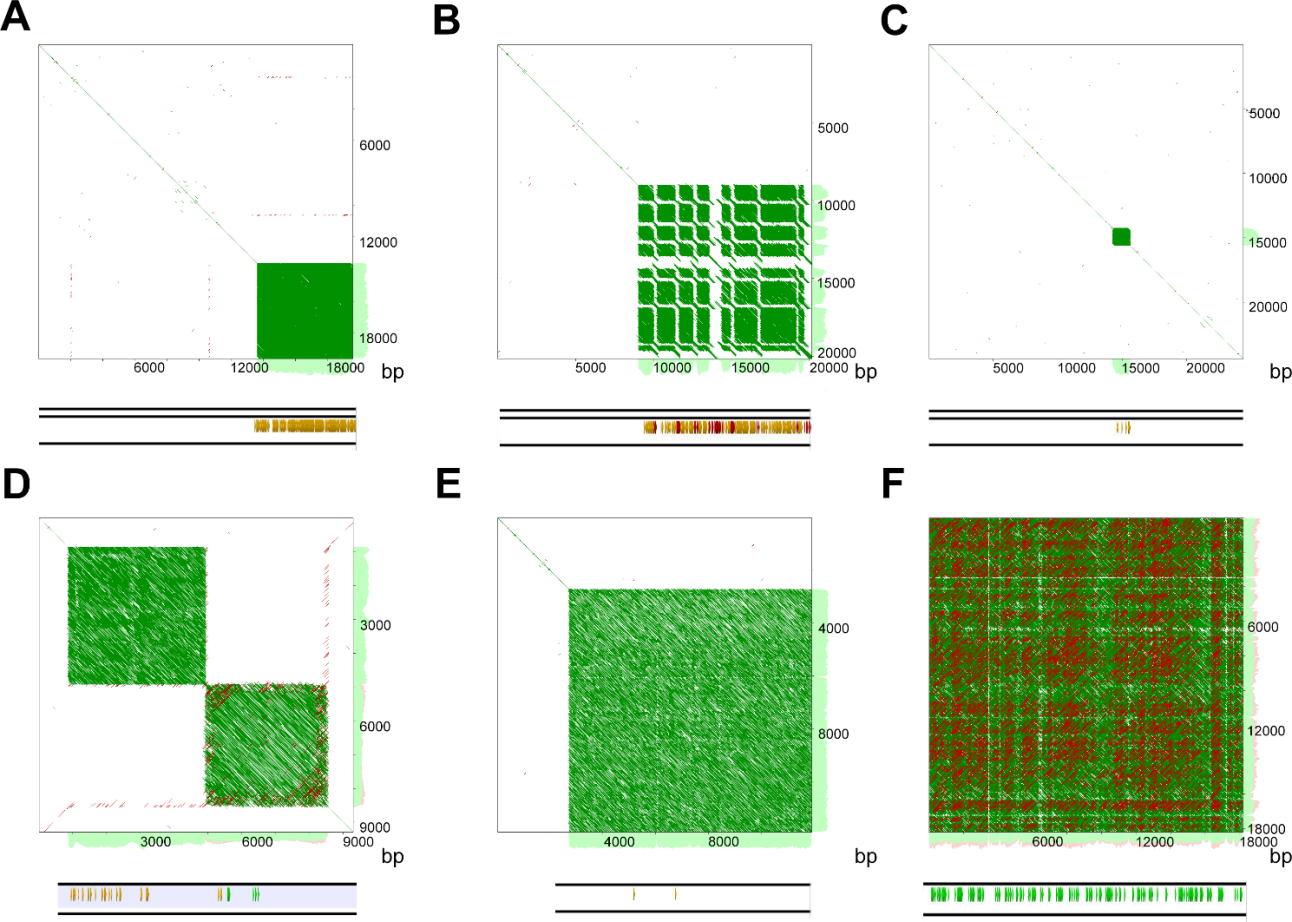
Analysis of the ON reads with telomeric sequences and their derivatives in the genomes of *Chenopodium* species. Self-to-self comparisons of the ON reads displayed as dot plots (YASS program output) (Supplementary information), where parallel lines indicate tandem repeats (the distance between the diagonals equals the lengths of the motifs; green lines are forward, red lines are reverse) at the top. Arrays of telomeric sequences in ON reads detected by the “search for motifs” command of Geneious Prime software based on of telomeric monomers at the bottom. Yellow – (TTTAGGG)_3_, red – (TTTAAAA)_3_ and green – ((C)CCTGGG + (C)CCTAGG)_3_. (**A**) Array of the *Arabidopsis*-type monomer TTTAGGG at the edge of the ON read (*C. pamiricum*, accession 830-3С, read #21 644 of 19 572 bp, see also Data S1). (**B**) Block-organized double-monomer terminal telomeric arrays where clusters of TTTAGGG monomers interchange with clusters of TTTAAAA monomers at the edge of the ON read (*C. acuminatum*, accession 429-3, read #5413 of 20 152 bp, see also Data S2). (**C**) Array of the *Arabidopsis*-type monomer TTTAGGG in the middle of the ON read, ITR array (*C. acuminatum*, accession 429-3, read #1954 of 24 378 bp, see also Data S3). (**D**) Colocation of ITR and DTR arrays (*C. pamiricum*, accession 830-3С, read #36 779 of14 352 bp, see also Data S4). (**E**) ITR array where elongated monomers are forming (*C. pamiricum*, accession 177, read #10 528of 11 922 bp, see also Data S6). (**F**) DTR array where elongated monomers are forming (*C. acuminatum*, accession 429-3, read #8544 of 18 185 bp, see also Data S7)

**Fig. 2:**
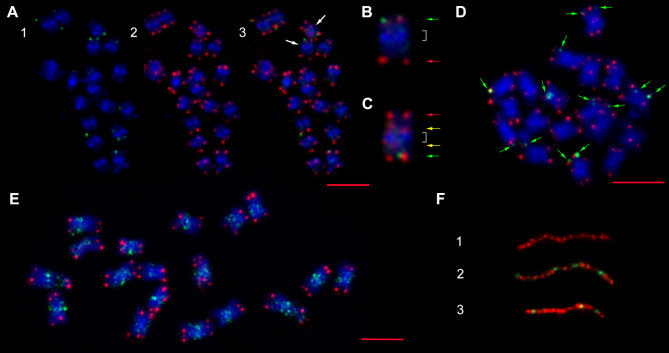
Multi-FISH with telomeric sequences of the *Arabidopsis*-type labelled with Cy3 (red signal) and derivative sequences labelled with biotin (green signal) in the somatic chromosomes of *Chenopodium* species. (**A**) Somatic chromosomes of *C. acuminatum*. (1) Non-canonical TTTAAAA probe (green channel). (2) Canonical TTTAGGG telomeric probe (red channel). (3) Combination of non-canonical and canonical telomere probes (red-green-blue channels). (**B**, **C**) An enlargement of the two chromosomes from the metaphase plate (shown with white arrows on A) showing chromosomal constitution. Green arrows indicate the position of combined TTTAGGG + TTTAAAA cluster-organized sequence arrays, red arrows indicate the positions of terminal telomeric arrays of the *Arabidopsis*-type, yellow arrows indicate the positions of the ITRs, and white square brackets show pericentromeric heterochromatin. (**D**) Somatic chromosomes of *C. iljinii* (433-9). Green arrows indicate the position of combined TTTAGGG + TTTAAAA cluster-organized sequence arrays. (**E**) Somatic chromosomes of *C. iljinii* (441-6) hybridized with sequences of the *Arabidopsis*-type (red signal) and DTR CCTGGG (green signal). All chromosomes were counterstained with DAPI (blue signal). Bars represent 5 μm. (**F**) Fiber-FISH conformation of ON data on the interchange of monomer blocks within the telomeric array on DNA strands of *C. pamiricum* (830-3 C). (1) Pure TTTAGGG array (red signal); (2, 3) two examples of combined TTTAGGG (red signal) + TTTAAAA (green signal) arrays

Analysis of ON libraries revealed the presence of multiple terminal and interstitial telomeric arrays in the investigated genotypes (Fig. 1). The basic monomer of telomeric repeats for *Chenopodiaceae* was previously identified to be of the *Arabidopsis* type (TTTAGGG) [6]. However, unexpectedly, we found significant variations in both the monomers themselves and the structures of both terminal and interstitial telomeric arrays. The *Arabidopsis*-type monomer clearly dominated in the genomes of the investigated species, where it formed long arrays on the edges of ON reads that appeared in dot-plots as blocks of tandemly organized microsatellites (Fig. 1A and Data S1). Arrays were mainly homogeneous, except for a small proportion (2–5%) of monomers that differed by one or two nucleotides, which can be explained by both method error and the reasons described below. FISH analysis showed mostly terminal positions of these arrays (Fig. 2A-E). However, in addition to “pure” arrays composed of *Arabidopsis*-type monomers, combined telomeric arrays where blocks of TTTAGGG motifs were interspersed with blocks of TTTAAAA motifs were found (Fig. 1B and Data S2). FISH analysis confirmed that TTTAAAA blocks are located in the terminal regions of several chromosomes of the set, are visible on one or on both chromatids, and fit positions with the *Arabidopsis*-type terminal telomere repeats (Fig. 2A-D). Fiber-FISH analysis reconfirmed ON data on the interchange of monomer blocks within the telomeric array (Fig. 2F). Block-organized double-monomer (TTTAGGG plus TTTAAAA) sequence arrays were found in different proportions in all analyzed genotypes, except one accession of *C. pamiricum* (177) and one of *C. iljinii* (461-5) (Table 2). For the two latter genotypes where TTTAAAA arrays were not found, we additionally scanned the entire ON library of 1,056,966 and 2,500,000 reads, respectively, for their presence, but with the same result. Previously, monomer variations within terminal telomeres were detected in the proximal part that flanks the Xp/Yp pseudoautosomal regions (PAR1) of human chromosomes [18],, and intermixing of the two non-canonical telomere sequence variants (T)TTCAGG on *Genlisea hispidula* chromosome ends [19]. Recently, a similar structure of telomeres in the human genome was described by Tan et al. [20]. Based on a comparison with PacBio data for the same genotype, the authors concluded that this is a bioinformatics error. Our data, in contrast, show that block-organized double-monomer telomers could physically exist in single genotypes. We detail our arguments in a separate paragraph “Arguments for physical existence of block-organized double-monomer telomers” in the Methods section. If we are correct and the structure does physically exist, then the Tan et al. [20] data on both the human genome and the genomes of several other model eukaryotic species including *Arabidopsis thaliana* support the ubiquity of the combined telomeres formation in individual genotypes (Tan et al. also determined absence of double-monomer blocks in one of two genotypes of *Caenorhabditis elegans*).

**Table 2:** Telomeric and their derivative sequences frequencies in the 50,000 longest reads of the studied *Chenopodium* species ON libraries

Species	Accession #	Number of ON reads with TS arrays	TTTAGGG	TTTAGGG + TTTAAAA	CCTGGG + variants
*C. acuminatum*	429-3	43	4	10	29
* C. iljinii*	433-9	30	12	4	14
	441-6	21	12	2	7
	461-5	32	15	0	17
* C. pamiricum*	177	17	10	0	7
	830-3 C	35	4	17	14
*C. suecicum*	328 − 10	22	2	6	14

**Fig. 3:**
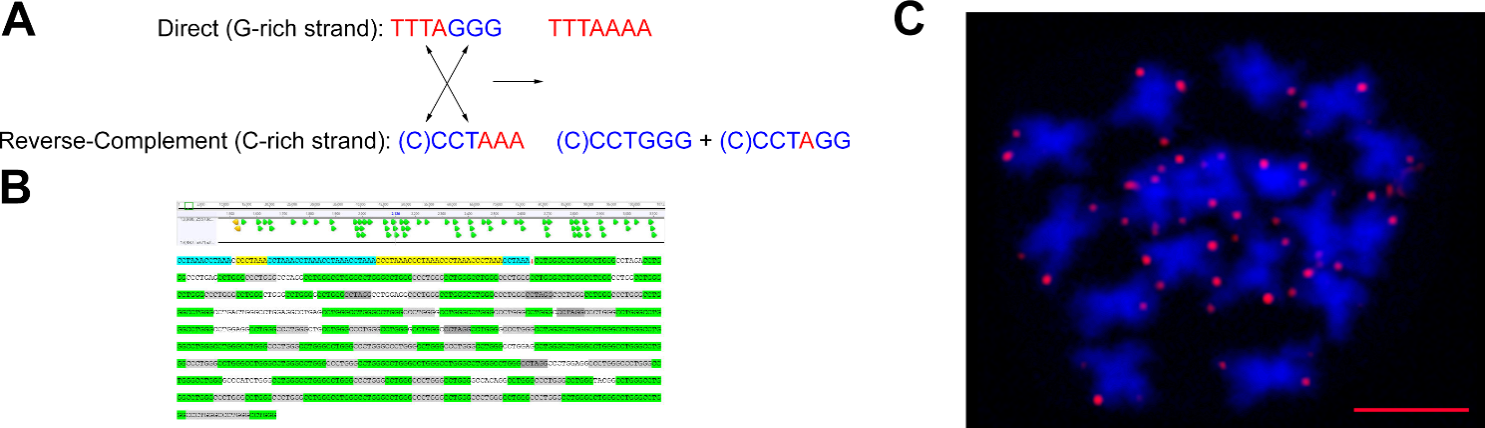
Putative recombination between G- and C-rich strands. (**A**) Scheme of the alleged exchange between DNA strands with the formation of derivative monomers that physically exist in the genomes of the studied species. (**B**) ITR-DTR junction (*C. iljinii* 433-9 read #13,986, see also Data S5). ITRs: TTTAGGG – yellow, TTTAGG and TTAGGG (small percentage, approximately 2–5%) – blue; DTRs: CCTGGG – green, CCCTGGG and CCTAGG – grey. (**C**) FISH on somatic chromosomes of *C. iljinii* (441-6) of the synthetic probe mimicking junction of TTTAGGG and TTTAAAA blocks

The highest variability was observed in ITRs, as expected. These repeats can be identified in ON libraries by their location in the middle of a read (Figs. 1C and 2C and Data S6, S7). In addition to the *Arabidopsis*-type, monomers with different substitutions and indels were observed within ITR arrays (Data S3). We observed that ITRs are often colocalized with arrays of cognate microsatellites consisting of the dominant monomer (C)CCTGGG interspersed with (C)CCTAGG that have a dispersed mode of chromosomal distribution and are located predominately in pericentromeric heterochromatin (Figs. 1D and 2E and Data S4). The latter two resembles recombinants of G-rich (TTTAGGG) and reverse-complement C-rich (CCCTAAA) DNA strands of telomeric monomers (certain helicases have the ability to disrupt G-G (Hoogsteen) base pairs, a feature that might be necessary to remove G base-paired structures from the 3′ TTTAGGG repeat overhang so that it can anneal to the CCCTAAA repeat strand of the duplex telomeric tract [21]). The scheme of the hypothesized exchange between DNA strands with the formation of derivative monomers specific to both terminal TTTAAAA and interstitial (C)CCTGGG telomeric arrays that physically exist in the genomes of the studied species is shown in Fig. 3A. In addition to array collocation, we found four points in the genomes of three different species where a few copies of TTTAGGG were flanked by proposed derivative telomere repeats (DTRs, see below) of (C)CCTGGG monomers oriented in opposite directions (Fig. 3B and Data S5). These junctions appear to be sites for the derivative monomer’s formation captured at the time of the event or shortly thereafter. Additionally, we determined chromosomal positions of junctions between blocks of TTTAGGG and derivative TTTAAAA by hybridizing chromosomes of *C. iljinii* with the synthetic probe (TTTAGGG)_5_ + (TTTAAAA)_5_ of 70 bp totally (Figs. 3C and 5). This experiment when the signals from the synthetic probe with a known nucleotide composition were determined at the part of chromosomal termini, confirms previously obtained data on the existence in the genome and terminal position of block-organized double-monomer telomeres (see above). It should also be emphasized that although TTTAGGG monomer mixing with or adjacency to the derivatives TTTAAAA or (C)CCTGGG is constant in ON libraries, but an array composed of only derivative monomers together (TTTAAAA plus (C)CCTGGG) has never been found. This result, most likely, indicates that the basic derivative monomers are not formed simultaneously. The particular mechanism of recombinant motif formation remains unclear, but it probably results from double-strand breaks with successive strand exchange, especially considering the fragile nature of TSs and the fact that they are considered recombination hotspots [15]. Alternatively, the mechanism by which telomeric units are formed and their structural variation and changes in copy number may result from local amplification according to a bidirectional rolling cycle model based on intermediate RNA synthesis as a primer, similar to DNA replication in bacteria. RNA sequences are rich in guanine, which forms stable G4-quadruplex structures and noncomplementary pairs (e.g. G-T, G-A, or both). As a result, structures with noncomplementary bases and convergence of A to C or T to C can periodically form in telomeric arrays [22]. Further elongation of a block of modified monomers may be linked to a t-loop replication mechanism (attributed to alternative telomere lengthening, ATL) when under certain conditions, the t-loop enters a prokaryotic recombination-dependent (or break-induced)-like replication pathway and may amplify blocks of TTTAAAA [21, 23]. We can propose that the appearance of combined arrays at the part of chromosomal termini may have far-reaching consequences. During meiotic prophase I, the pairing of homologous chromosomes for meiotic recombination occurs. This movement involves clustering of telomeres at the nuclear membrane to form the so-called telomere bouquet. Any changes in telomere length, composition, or both can disrupt (or partly disrupt) bouquet configuration. This causes enhanced genomic rearrangements, aneuploidy and thus produces variants for natural selection.

**Fig. 4:**
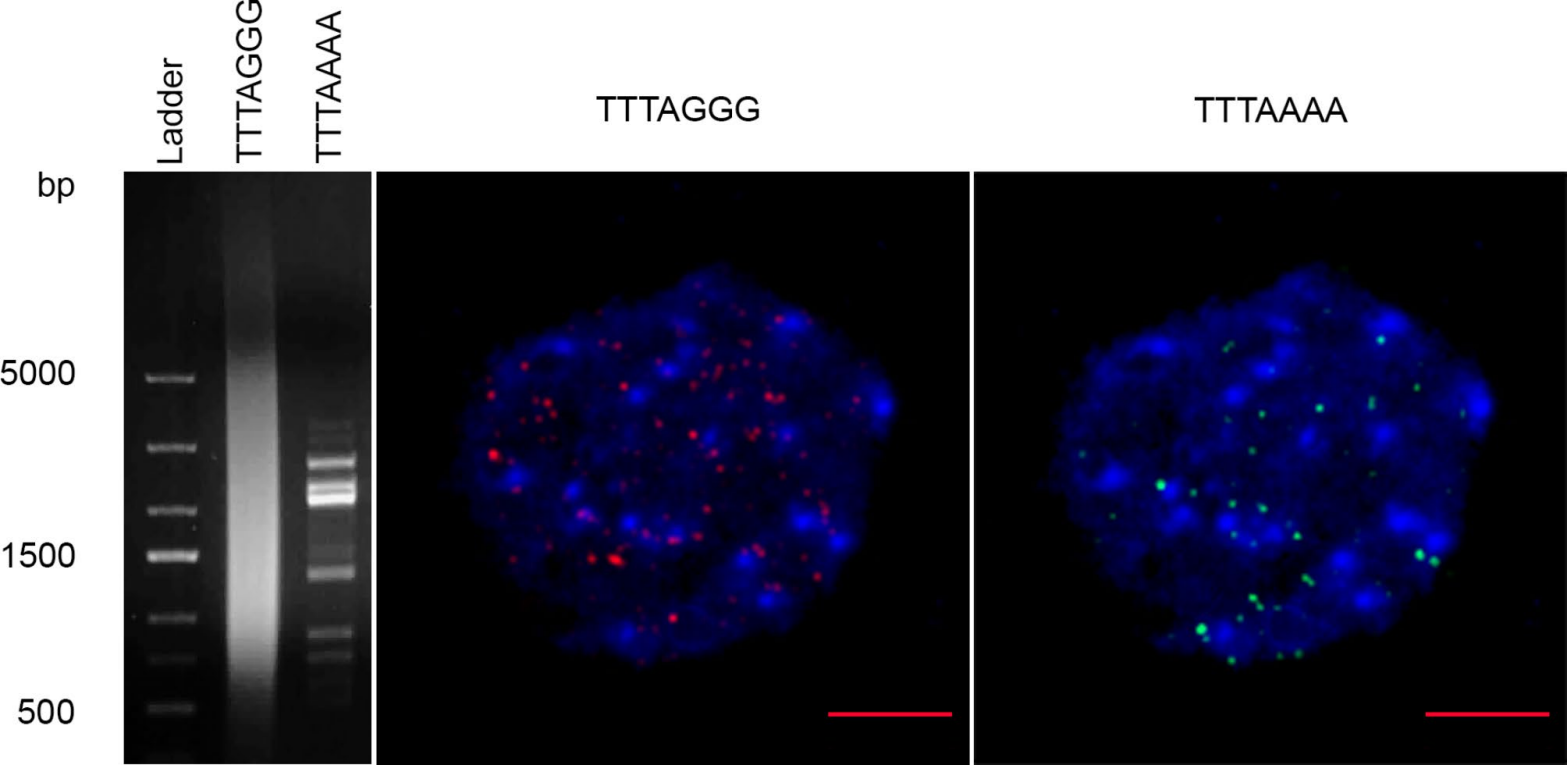
Probes for in situ experiments. PCR products obtained with forward (TTTAGGG)_3_ and reverse (CCCTAAA)_3_, and (TTTAAAA)_3_ primers (left). Hybridization of labelled PCR products on the interphase nucleus of *C. acuminatum* (central and right). Bar represents 5 μm

**Fig. 5:**
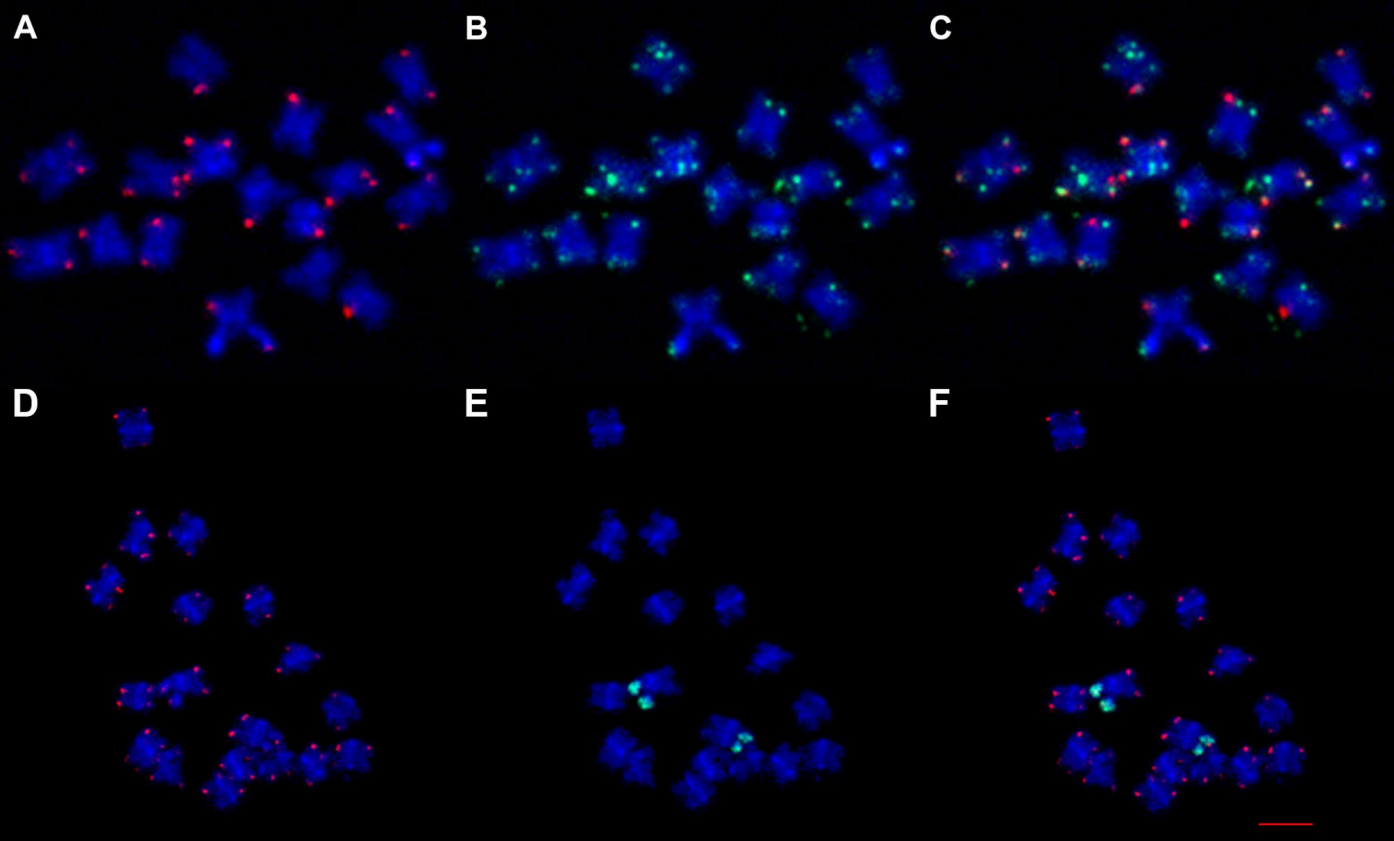
Hybridization of synthetic probe mimicking junction of TTTAGGG and TTTAAAA blocks together with telomeric probe on chromosomes of *C. iljinii* (**A-C**). (**A**) Synthetic probe (TTTAGGG)_5_ + (TTTAAAA)_5_ (red signal). (**B**) Telomeric probe (green signal). (**C**) Combination of synthetic and telomere probes. Control experiment for determination of the efficiency of synthetic probe hybridization on chromosomes of *C. acuminatum* (**D-F**). Simultaneous FISH of synthetic probe and 45 S rDNA (pTa71) was performed. (**D**) Synthetic probe (TTTAGGG)_5_ + (TTTAAAA)_5_ (red signal). (**E**) 45 S rDNA (green signal). (**F**) Combination of synthetic and 45 S rDNA probes. Bar represents 5 μm

Another determined form of TS variability identified in the genomes of the investigated species is the formation of longer monomers based on the TTTAGGG sequence. The process occurred exclusively in the ITR and DTR arrays in the chromosome internal (Fig. 1E, F). Tandem Repeat Finder (TRF) revealed that new monomers derived from the original TTTAGGG sequence can reach a length of up to 195 bp (49–66 different telomere motif-based consensus monomers in total per array) in ON libraries of the investigated *Chenopodium* species (Data S6 and S7). This can be considered as the first step towards high-order repeat (HOR) unit development when a complex monomer is formed from modified repetitive motifs by concurrent amplification and homogenization in the original satDNA [24, 25]. Monomer elongation within arrays of ITRs and DTRs seems to be a common trend in the plant satellitome, as a similar process was recently described for species of the genus *Arachis* [26].

In summary, while maintaining a dominant presence of the *Arabidopsis*-type telomeric heptamer, several derivative monomers arose in the genomes of all *Chenopodium* diploids studied. We detected the formation of different DTRs in part of both terminal and interstitial telomeric arrays. The former is a case of block-organized double-monomer telomeres. The latter is an integral part of satellitome evolution, with transformations specific to the inactive genome fraction [24, 27]. The derivatization of TSs is most likely a permanent process in the plant genome. Confirmation of this assumption would provide a comparison of the identified DTR frequencies in the genomes of the studied species (Table 2). The ON library of *C. acuminatum* appears to be the most enriched with DTRs. The species is ancient (originating at the Miocene-Pliocene border) with a heterochromatinized genome [28]. We propose that *C. acuminatum* underwent numerous of breakage and repair events during its long evolutionary history, which would lead to the accumulation of DTRs.

We did not find any qualitative interspecific differences in the presence of DTRs: all studied species from boreal *C. suecicum* to semidesert *C. iljinii* had all types of determined variants. However, there is a clear intraspecific variability in the occurrence and frequencies of DTRs of different types. This is indicated by differences between the populations of *C. pamiricum* and *C. iljinii* with contrasting environments. Thus, the lowest number of DTRs with complete absence of combined terminal telomeric arrays was found in a population of *C. pamiricum* (accession 177) located at 1939 m in the Altai Mountains valley close to the last remnants of the Mammoth Steppe, while the genotype from the population located at 3937 m (accession 830-3 C) exhibited enhanced DTR frequencies with the highest number of combined terminal telomeric arrays (Table 2). This comparison reasonably raises the question: can the determined variability result from the processes associated with high-mountain adaptations, or, in other words, if DTRs frequencies are ecologically dependent? The analyzed genotypes of *C. iljinii* from populations located 450–700 km from each other also showed population-specific DTR patterns. Despite these clear intraspecific differences, the causes and consequences of DTR formation remain to be elucidated, and more definitive empirical evidence is required before TSs variability in natural plant populations can be confidently assessed.

## Conclusions

It was assumed that derivatization of TSs is a ubiquitous process in the plant genome but occurrence and frequencies of derivatives may be genotype-specific. We also propose that the formation of non-canonical arrays of TSs, especially at chromosomal termini, may be a source for genomic variability in nature.

## Methods

### Plant material, DNA extraction, Oxford Nanopore library preparation, and sequencing

The following species were used for preparation of DNA libraries: *C. acuminatum* Willd., *C. iljinii* Golosk., *C. pamiricum* Iljin, and *C. suecicum* Murr (Table 1). Seed samples were collected in the field by us or our collaborators in various parts of Eurasia [16]. All plants were cultivated at the experimental garden of the Institute of Botany, Czech Academy of Sciences, Průhonice, Czech Republic. DNA was extracted using the DNeasy Plant Mini Kit (Qiagen) according to the manufacturer’s instructions as described previously [16]. For in situ hybridization experiments, tips of the young, fine roots were collected and fixed and then stored until use.

ON libraries were prepared from 1 µg of DNA using an SQK-LSK109 Ligation Sequencing Kit (Oxford Nanopore Technologies) following the manufacturer’s protocol. The DNA was treated with 2 µl of NEBNext FFPE DNA Repair Mix and 3 µl of NEBNext Ultra II End-prep enzyme mix in a 60 µl volume that also included 3.5 µl of FFPE and 3.5 µl of End-prep reaction buffers (New England Biolabs). The reaction was performed at 20 °C for 5 min and at 65° C for 5 min, followed by purification using a 1x volume of AMPure XP beads (Beckman Coulter). Subsequent steps, including adapter ligation using NEBNext Quick T4 DNA Ligase and library preparation for sequencing, were performed according to the provided protocols. The entire library was loaded into a FLO-MIN106 R9.4 flow cell and sequenced for 20 h. Basecalling was performed with basecaller Guppy v6.0.1. The error rates for ON sequencing were determined elsewhere [29, 30] as follows: within 1.6–2.7% for deletions, 1.2–2.2% for mismatches, and 1.1–2.4% for insertions. The error variation is not high (standard deviation ca. 0.1% for deletions).

### Search for telomeric repeats in ON libraries and data processing

Geneious Prime software, version 2022.1.1 (https://www.geneious.com) was used for the processing of ON data and the identification of telomeric sequence arrays in the genomes of all investigated species. The 50 000 longest ON reads in each library (average genome coverage 0.7x) were scanned for the presence of the telomeric sequences with the aid of the motifs. The *Arabidopsis*-type microsatellite was identified using the motif (TTTAGGG)_3_ as a query. The DTRs were identified using motifs (TTTAAAA)_3_ and (CCTGGG)_3_. Scanning was performed by using the “search for motifs” command of Geneious software with zero mismatches. For two genotypes *C. iljinii* (461-5) and *C. pamiricum* (177), where TTTAAAA arrays were not found, we additionally scanned the entire ON library of 2,500,000 (4.4x genome coverage) and 1,056,966 reads (1.8x genome coverage), respectively, where these arrays were also not found. This was done to avoid questions about undersampling. Each selected ON read that contained telomeric sequences was further analyzed on public web servers: (i) YASS genomic similarity tool (http://bioinfo.lifl.fr/yass/yass.php) and (ii) TRF (https://tandem.bu.edu/trf/trf.html). YASS allows for searches of more fuzzy repeats for potential tandem organization [31], visualized by dot-plots comparing each contig against itself (Fig. 1). TRF was used to determine of telomeric sequence array structures and consensus monomers [32] (Data S1-S7). The algorithm of TRF searches for tandem repeats that are often hidden in larger homologous regions or that may fall well below the level of significance required for other programs to report a match. The detection criteria are based on a stochastic model of tandem repeats specified by percent identity and frequency of insertions and deletions rather than some minimal alignment score and align repeat copies against a consensus sequence, which reveal patterns of common mutations [24]. The described algorithms were applied to each read with TSs array of ON library.

### Slide preparation in situ probe preparation and FISH procedure

For slide preparation, root tips were pretreated in 0.002 M 8-hydroxyquinolin for 3 h in the dark and fixed in 3:1 (v/v) 100% ethanol:acetic acid. The fixed root meristems were thoroughly washed in water and enzyme buffer (10 mM citrate buffer, pH 4.6) and partially digested in 0.3% (w/v) cytohelicase, pectolyase, and cellulase (Sigma, St. Louis, MO, USA) at 37ºC for 3 h, followed by several washes in water. The material in a water drop was carefully transferred to a grease-free microscope slide, and cells were spread as previously described [24]. Fiber-FISH slides were prepared from the total DNA of *C. pamiricum* (830-3 C) according to the technique described by Schwarzacher and Heslop-Harrison [33].

FISH was performed to detect the chromosomal distribution of the telomeric sequences of the *Arabidopsis* type and its derivatives in the chromosomes of the investigated *Chenopodium* species. This method provides sufficient resolution to determine whether the block of telomeric repeats is in the terminal or interstitial position [19]. Probes for in situ experiments were produced using the approach described in [34]. PCR was performed in the absence of template using the following primers: for the *Arabidopsis* type, forward (TTTAGGG)_3_ and reverse (CCCTAAA)_3_; for derivatives, single forward (TTTAAAA)_3_ (Fig. 4), and another derivative forward (CCTGGG)_3_ and reverse (CCCAGG)_3_. Staggered annealing of the primers provided a single-strand template for extension by Q5 polymerase (NEB). The primers served as templates in the early PCR cycles, whereas the newly formed templates served as primers and templates in subsequent stages of PCR, resulting in a heterogeneous population of molecules comprising repeat arrays of various lengths. The reaction cycle consisted of 2 min at 98 °C, followed by 10 cycles of 98 °C for 30 s, 35 °C for 30 s and 72 °C for 1 min, 35 cycles of 98 °C for 30 s, 35 °C for 30 s and 72 °C for 1.5 min, and final extension at 72 °C for 10 min. Purified PCR products were labelled with biotin (biotin-16-dUTP, Roche, Basel, Switzerland) and Cy3 (Amersham, Amersham, UK) according to a standard oligo labeling protocol. For determination and localization of the of block-organized double-monomer telomeres by FISH we mimicked the TTTAGGG and TTTAAAA blocks junction by designing the synthetic probe (TTTAGGG)_5_ + (TTTAAAA)_5_ 70 bp totally (TTTAGGGTTTAGGGTTTAGGGTTTAGGGTTTAGGGTTT AAAATTTAAAATTTAAAATTTAAAATTTAAAA). The probe was prepared and labelled by Cy3 by Eurofins Genomics, Ebersberg, Germany (Fig. 5A,C). We also performed control experiment for determination of the efficiency of synthetic probe hybridization by simultaneous FISH of synthetic probe and 45 S rDNA (pTa71) (Fig. 5D-F). FISH was performed in a ThermoBrite programmable temperature-controlled slide processing system at 42 °C overnight. Biotin was detected with fluorescein isothiocyanate (FITC)-conjugated avidin (Molecular Probes, Eugene, OR). Slides were stained with DAPI, mounted in antifade mountant (Vector Laboratories, Peterborough, UK) and examined and photographed on a Zeiss Axio Imager.Z2 microscope system.

### Arguments for physical existence of block-organized double-monomer telomers

Based on the analysis of ON sequencing data of the CHM13 sample of the human genome, Tan et al. [20] described a similar phenomenon, namely appearance of (TTAAAA)n blocks within a telomeric array of TTAGGG monomers, which the authors interpret as a “bioinformatic error”. These conclusions were made based on comparison with PacBio data from the same sample where corresponding structures were absent. It is difficult for us to evaluate the difference between the ON and PacBio platform algorithms, but we can also propose the converse situation where PacBio smooths out subtle fluctuations in telomeric repeats. As the TTAAAA blocks were interpreted as an error, the authors attempted to eliminate this via programmatic methods, although it is not clear from the publication whether they managed to do so completely. In this situation, it is unclear if computer manipulation reveals the true physical structure of the telomeric arrays.

On the basis of our data, and primarily on molecular cytogenetic results, we assume with a high degree of probability that combined arrays physically exist in some eukaryotic genotypes. Our argumentation is presented as follows:

- The strongest argument in favor of the existence of the block-organized double-monomer telomers is their genotype specificity, namely the presence in some genotypes and the absence in others. If this were a method error, then these would be present in all libraries in a varying degree. However, we found two genotypes that did not contain combined arrays at all, while others contained telomeric arrays with and without TTTAAAA blocks (Table 2). Moreover, Tan et al. also provide data on the absence of blocks with errors in one of two genotypes of *Caenorhabditis elegans*, which confirms our data. Further, the authors concluded that “Together, our results suggest that repeat calling errors in nanopore sequencing can be observed at telomeres of some other organisms beyond human telomeres”. From our perspective, it is more probable that genotypes with and without combined arrays were analyzed, and the CHM13 sample belongs exactly to the group of genotypes that contain the block-organized double-monomer telomers.
- Another argument is the differences between terminal and interstitial telomeric arrays. In the latter, the numbers and variability of monomer variations are much higher (see Data S4, S6 and S7) but must be almost the same if this is just an error.
- One of arguments of Tan et al. is that errors are very often found at the ends of a read. This is not surprising considering the chromosomal position and fragile nature of telomeric sequences, and it can be assumed that the DNA strand breaks off at these sequences during ON analysis (telomere sequence damage during analysis may also be among the reasons why PacBio does not identify such types of telomeres).
- Tan et al. do not provide any molecular data to support their conclusions.
- We have performed several experiments that confirmed the presence of combined telomeric arrays. In particular, a clear in situ signal was obtained by hybridization on the metaphase chromosomes (Fig. 2A-D) and interphase nucleus (Fig. 4) of the *Arabidopsis*-type telomeric probe and derived TTTAAAA repeats probe.
- Another approach was in situ hybridizing chromosomes of *C. iljinii* with synthetic probe (TTTAGGG)_5_ + (TTTAAAA)_5_ that mimic junctions between blocks of TTTAGGG and derivative TTTAAAA in terminal telomeres (Figs. 3C and 5). This experiment when the signals from the synthetic probe were located at the part of chromosomal termini confirms previously obtained data on the existence in the genome and terminal position of block-organized double-monomer telomeres.
- The third approach was fiber-FISH, where TTTAGGG and TTTAAAA probes were hybridized to DNA fibers on the slide. Fiber-FISH analysis reconfirms ON data on the interchange of monomer blocks within the telomeric array (Fig. 2F).

At an early stage of our research when the unusual structure of the telomeric array was determined, we believed that the appearance of TTTAAAA monomers were a result of recombination of DNA strands and the further formation of combined block-organized telomeres should be a general phenomenon due to the conservatism of telomeric monomers. However, we did not have the opportunity to test this on other species. The paper of Tan et al. provides a strong argument in favor of the universality of the discovered phenomenon.

### Electronic supplementary material

Below is the link to the electronic supplementary material.

Supplementary Material 1

## Data Availability

The datasets generated and/or analyzed during the current study are available in the public Zenodo repository, 10.5281/zenodo.6517222. The datasets for accession numbers are as following: *C. acuminatum* accession 429-3 https://zenodo.org/record/6517222/files/429-3-acuminatum_ON_50000.fasta?download=1, *C. iljin*ii accession 433-9 https://zenodo.org/record/6517222/files/433-9-iljinii_ON_50000.fasta?download=1, accession 441-6 https://zenodo.org/record/6517222/files/441-6-iljinii_ON_50000.fasta?download=1 and accession 461-5 https://zenodo.org/record/6517222/files/461-5-iljinii_ON_50000.fasta?download=1, *C. pamiricum* accession 177 https://zenodo.org/record/6517222/files/177-pamiricum_ON_50000.fasta?download=1 and accession 830-3 C https://zenodo.org/record/6517222/files/830-3 C-pamiricum_ON_50000.fasta?download=1, and *C. suecicum* accession 328 − 10 https://zenodo.org/record/6517222/files/328-10-suecicum_ON_50000.fasta?download=1.

## Abbreviations

TSs: Telomere sequences
ITRs: Interstitial telomeric repeats
ON: Oxford Nanopore
FISH: Fluorescent in situ hybridization
DTRs: Derivative telomere repeats
ATL: Alternative telomere lengthening
TRF: Tandem Repeat Finder
HOR: High-order repeat
