# Supplementary material for: Telomere sequence variability in genotypes from natural plant populations: unusual block-organized double-monomer terminal telomeric arrays
Source: BMC Genomics. 2023 Sep 26;24:572. doi: 10.1186/s12864-023-09657-y (PMC10521516; doi:10.1186/s12864-023-09657-y)
Supplement: Supplementary file 1 — Supplementary Material 1 [file 12864_2023_9657_MOESM1_ESM.docx]

**Additional file 1**

**Data S1.** Example of telomere sequences array of *Arabidopsis*-type

*C. pamiricum*, accession 830-3С, read 21644 (19572 bp). Refer to Figure 1A.

TRF analysis:

Sequence: 830-3С_read_21644

Parameters: 2 7 7 80 10 50 500

Length: 19572

| Indices | Period Size | Copy Number | Consensus Size | Percent Matches | Percent Indels | Score | A | C | G | T | Entropy (0-2) |
| --- | --- | --- | --- | --- | --- | --- | --- | --- | --- | --- | --- |
| 837--881 | 21 | 2.3 | 19 | 88 | 7 | 63 | 57 | 4 | 11 | 26 | 1.52 |
| 3955--4049 | 2 | 47.5 | 2 | 100 | 0 | 190 | 50 | 0 | 0 | 49 | 1.00 |
| 13662--19560 | 7 | 837.7 | 7 | 90 | 7 | 9348 | 14 | 0 | 42 | 43 | 1.46 |

Consensus pattern (7 bp):

TTTAGGG

Sequence:

>830-3С_read_21644

CATTGCTCAAGTAATATCAGATGGTACTGCAAGATACTTACAAAAATATAGAACGAAATGATAAGAAAAAGCTCAAAGCGCTGGGCGAATTTAGAAGAATCAAGGGTGATGTCAGACTCCGACGGTCAAGTATATCGATTAATCAGGAATACATTAATTAAGTTGTAAATAACAAGTCCAAGCTATAAGCGCAGGCAATAAATCATTGACTAAAATTCCACCCTTTCCGTCAGCGCAATAATAATAAAATAATACTCATACAACAGAGGATAGCAGAATTAAAGGAAAGATTTAGAAATTAAAAATACCTGTGTTTTGGAGGATGAGTAATAAGGGCGTTGGCAGAGAACCAGAGGATTAGAAGGAGGATGAGCCGGAATCGTTACAAGAAGCACCACCTGTAACCAAATCTCGCATCGCCATGATAGACCAGATTTAGCTGCTGAAGATGAATTACCCTCTCTGTCCAATCTCCACCCACCCTCTCGCCTTCCTCTTCACCGCTCATTGCACTCCCTCTCTCTGACTCACTAATTCTAACATGTCAAATTGGATTGCCAATGTTGATAATAATTCATTAATTATGATAAATCAACGGGAATAAATGAAAACAAAACAAAAAGAATCGAAGAGAAAAACAAAAAACTAAAAATAAAAGAGTCCAAGCTAAAGAAAGAAAAAAAGATAGAGAGAAATTGGGGATTTAATAAACCGACAATGTTTATGGAAATGTCAATTGGTCTTATGCACCTTAGATAGGTGCAGTGGCGGCGTCTTGGTACAAGGTAATACTTGCGACAACGTATCATGATCATAAAATCAATATTTCTATTTCTAAAATGAGCATTTAATAAAAAAATGAAGCGATTTAATAATAAAATAAACCACGTTAAAAATGAACATTTAAAACTTATAATGCATATGTTTAAACCATATTATTTTTATTTCTTCAAATTTTATGTTAAATGCGTATATTCATAATAATATTTTATATGTTCATATTTAATTATAATATAAATCGCGAAAACAAGGTCAAAAAAGAAGAGAAAAGAAGTGTGAATAGTGAAAATGTCTAAATAATGCAATTAAATAAAGAGAAAATATTTAACAAATTATATAAAATTATTCTGAATTAACAAATTACATAACTATTTAATAATTATAAAAATAAATGATTTTTTTAATAATCATTTATATAAGACCTCCAAATTGTCAAAGTGAAAATATGTAAGTAAAGTTTAAAACATATGTAAGTAAATTGAAATTAAAGCATTAAAAAAAAGTAAATTATACGAGATAATTAGCAGGCAAGAGGAAGGAGATTTATGCTCAAATCATCTAAATCATGAATATTACCAAAAACCCTATTCAAAAACCACAAAATGTGAAAAAGAAGCACAAGAAATTAAATCGAGAAAATTGAAGAAAATAGAAAACTCAAGTAAATAAAAAAAAAAAAAAAAACTAGAAGGCGAAATTGATGAAATTAGGGAAAAACTCAAGCAAATTCACAATCTGGAAAAAAAAAGCTGAAATTTTTATGAAATACTTATGTTTTATTCACCAAATACCCATCAAAACTTTCAAATTCATAAATACTCACGGATTTAAGAAAAATGTCACCAAATGCCTTTAATGACTAAGCTGCGGTTAACTTAGTTAGTCAATCTTGATGTATGCCCGCGTGTGACCTCTAATTAGTTGTAATATTAGTTAATTAATTATTAAAAAACTTAAAATTTTAAAAAAAACTAAAAAAAAAAATAAGATACGCTTCCCCTGACGCCCCGCTGCTACCACCGGCCCACCGCACGGCGCCAGCCAGACCATGACCTCCGCTCCTGCTTGTAGGTGGCGATGTGACTCACTCCACACCCCGGACCTGTCTCCTCAGTTTCACTCCCTTAAACCCGCTCACAAGTAAGCGTAGAAAAAAATCCCCCTCCTTACATTTGTAGGCGTCAAACCCCCTCTCCCGGCGTAGAAGTGAGCCTTTTCGCCCTTTACATAAATCCCTTTGTTTCACCACTAAGCCACCACCCAAACCCACTTAAACCTTAATCGAAAAAACCCCAAACCGAAATCTAGGAGGTTTCGATTTGGGGGTTTGTTAGTGGTGGTTTAAGTGAATGGTAGATGGCAGTGGGCTGGCATTCGCCGGAGGGCCCAGCCGCTGCCCAGCAGGCAGGCTAAGTGAGTCGAGGGGTTATGAGGGATCTGTGAAGCCCAGGCTAGGGAAAGTGAAAAAGTGGGGAAATTTGACCATCATAGCAGGCGAGGGAGGGTGGGGCTGCTGACCCTTGCCATCGTCAGCGAGTGCAGTGGTGAGTGAGATATTGTTCTTAGGAGATTTTTTAGCAACAATGTTATTGAGTTGAGGTTCATGATCAAAATCTGCTTGCATATCATCAAAATTAGCACAAACCTCATCACAATGAGCATAAGAGGGAGGGGTCAACATGCATATTTGGATCATCCACAGAGAGCATATTAGAAGCATCAAGAGAATTTCATGCAAAAAATTAAAAGATCTTGACATTGAGAATCAAAACAAGAGAAACATGACATTCTATCATAGAATCTCTACTTAAACCAAGTGAAGAAATTCCAAGCATCATGCATAGACAAGATGGAATCTACCATCATACATATTCTCAACCGTTCCTAGTATGTTCATTTCATCTTGCAAATTTAGTATATTACAAATCAGCTAGAGAACCTTGTAGAGCAAAGGAAAATAATCTATCAAAGTAAGCATAGAAGGTTCATCATTAAGTGAGAAAGATAAGCATGCCATTTTTTTACAAAAAAACAAAAGAAAAGCAAACAAAAAATCAAATAATATACAAGAACCTAGCTAATCTAATCAACACACATTAAAATACGCCTTAGTCCCCTGACAGCGGTGCCAAAAAATTTGATCACGAGGTAGGGAGTAGGTAGTTGGGACTACTAACCACAACCAAATTAGAAATTTATAATACCTAAATTTACACTAAGCTAGTTAGTGTCAGAAAGGAATTAGAACCACAAGGACTAAGTGTATCAATGTATGCTAATTTGACTACTAGGATGATGCTAGAGATAAAGAGAGAGGGAACATCTAAAGCACTACTTAGAAACAACGAAAACTAGGGAAAGTGGAGATCCGATTACATCAATACATTCAACAACATCATAAAATTAGATGAAAATGTCTTGATTAGGAACGATTAACACAATTAACTGTTTCTCGCGCAATTCGCATGTTAATTATCCTACTAAACTAATTACAAACTTTGTAACTTTCAGTGCGTACGCGTGACCATACTAGTTGGTGGTTAATCCTCAAAATGAATCGTTATAAAAGCTTTCATAGGTATATAAATTGCAAAAAAAAAAAGTTTACATTAGATTAATTGATTAATCAAATCGGTATAAATTTATCGTTCTCATATTGACATTTAAGCATTAATTGTGACAAATGAGTATAAAATTTTGATTTGTATGAAAATTATTCTTTATTTCTTGGATGAGGCATTTAAGGTGCAAGTTGATTTTATTTTCAAAAAAAAATTGTTTCCTTTATTATTATATAGAGTATTATCTTTTGTATTGGATTCCTATTTGTTCTAACCTAGAAGTAAATGGCATAATTCTCTACTCTATGTAGGCTACAAGCAACTCTGACTCATGATAATATTTTGTTTGAGGCCACAAGGGCGGATGGCGAGAAATTGATCCAGCTCACTGCAAAAGTGGGAAAGAAGTTACAGCAGCTTGAAGCTATCTTTCCTTTCACTATCATGATAATTTGACTTAAGTGTTTCCTTATAGCAAGAAGGTAATGCAAATGAGGCCATATTACTTTGTTTCATGACATCATAATTTATATTTGAGATCAACAGGAAAATAAGATAGAATTTAGAAGAGAGTGAATCCACAAAAGGAACAACAATAAGATCAATTGATCAAATATATATATATATATATATATATATATATATATATATATATATATATATATATATATATATATATATATATATATATATATATATATATATATAATGTGACAAAAAAAGGAAGATTTGAATCACAATTAAGCTACCAAAGGATAGTGTTGAGTTAATTATTAAAGAATATGTATGATAAGGTCACTTAAATTCAAAACTACATATCTCATTTGTATTTCTGAGAAAAATTATAATGCCTTATAGTTCATTTGCACAAAAATAAATAAATAAAAATCACAAAGAGGAAGGAGAAGGAAAGAAATAGAACAATGAATTAGGCGGTTTCTCACCAAAGTGCATTTGGCTAGCTTCGCTTGATTTCTATTTCTTAAGTAGTTTTCTAGAAATTGTTTTTAGAGAAACACTTTTGAGAAATAAAATTGTTTAGTAAATAATCGAAGTGATTCGGTAAGAAACCATGTTTGGCTATTTTTGTTTATAACAATTTTACTTTTTAGCGCATTGTTTATTAACTTTGTTAATGCTCATTCACAATCCCCCTCGAAAAAAAAAAAAGAAAAGAAAAAGCGCATTCACCATTGAAGGGAACCCGTAGCACGATACCATTACCCTTCGGTCTCTGCTCCTCACTTTACCCTTTTCTCGGCAAACTACCACTAACCTCAAGAATTTTACGCCATCGACAACCTTAATCTATTACAACACTTGATACATGCATGACCACCAAAGGAATTTAATCAAATATAAACGTATTTTCGGTGCTTGTGGCTTTATCAAGAATGGTTTCTTAATTTTCGGTGAACGACAAAGGCAAGTAGTCTATTTTCCCTTCTCTTATGTTTAATTAAGTTTTGGGTTAGCAAGTCACATTGCCTTTGTGAATTTAATTGAAGTTTCTCTTATCACTTTAAAAGTAATTAGAATTTGGGAGTGTTCTGAGTTAAAGGAAGAAGATGAGAGAGGATATGGGTGATTGTTATATGGGCAATTGAGGATTTCATGATTGTTTGAGGTAATATTGACCCAAATAAAAGTTTCATTAAATGAGCAAAAGCTGCTCCCCAGAGAATTTGTATTGGCTCAATTTGGTGCTTACCTTCTAAGCCTCCTAGAAACTATTTTCGGTTTAGAAAAAGTAGAAACTACCTTCCAAAAAACATAGTAATTAATTTCCCAAATCTAACTATGGTTGCAAATATAAAATGAGAAAGTTTTCTCGTTAAGATAAACTGATTCATTTATCTGATGACAATTTTATAATTTTTACATTCTATTTATTTTTTGAAAAAATATCATTTTCATCTTCACCACTCGGATCGTGAGATTCATGATCCACCACATGGTTTTCTTCCTCCTCTTTCCTTCCTTCCTCGTGACTACCTACCTTAGTTCATCTTCTTCTTTCTCCTCCAATCCCAACCCACCTTCATCCTACTTTTTGTTTCCTTCTTTAATTTGTGATGAAAGAACACCAAATCAAAATTTCAGGAATACCTAAGGAAAAAAAAACTAAGGAATGGACAACACTAAAGGAAAGGAGTGTTTGTCATTTGGTTGTGAATTTTGTCCTTTATTGATTTCACCATTGGCGGTGCTCATGAGCTTATTCATCTTCTTGCTCAATCTCTATAGAGCGGCTATGAAACCTTTCTGAGCTTTATGGCAAAAGGGTCTTATTTTTAAAAAGGTAAAATGACTAAAACAGCCGTAATATATTTTGAAGTTATCGCGTTAAGAAAGCTTGGATACCATATGATATAGGATTAAACTGACCCATTGCCTGATGCTAGTACTCATTTTAAAATCATTCACAAAAGCTTAATTTTATACTTAGCGGTATAATCTTGACAATGTGTGAAGGGGTGTTAGTATTACACTTATCAATGCACAATTTATCTGAGTAAAAATAAAGAACAAATTAACTTATTTTGTCAGAATTACCTCTTCTGGCATGTTAAATCTGCTTTTTATAATTATTTTATTCCTTTTTTTCTAATCTCCTTTTTATTATCTTTAATTTGTATTATTAGCTTTGGTTTTTAAGAGTTATCTATGATTCATATGTATCCTTTGTATAAGAATAATGAATAAGTGACATACTAATTTATAATGGAAAAAAAAAGTTTGCTCAAAAAAATCAAACTTTATATGGAGTATGTGGAAACAAATTAGTGAACAACAATACAGTAAAACATTTGAACACAACTTTGAAATTACAAATATCTACGAACTATATCATTTACATTGTACACTTTTCTGCACCAAGACTTGAATTTGCAGCAAGTAATTACAATAATATCTAACCAGCATTATCTAATAATCATTTTCTGGTGTAGAAGTATCATATAGAGCCTTTAACACCTTCAGCTAATAGAGTATAGGATAATACCAGGCCAAACAGAAGCGTCGTTTACCACCAGAAGAAACCTGAAACAAGTACACTTCATTTCATCAGCCTGTTATCGAGATATTCACAGAATTAAGAATACTAGTAAAAAGCATGAGTACCACTACCATCTAAAATAATACAAATCCTCATTGTTGGGAAAGATTTAGATGGAAAATGGGAAATTCCTTTCCCTCCACAATATCAAACCAAAGTGCAAGTTTCCATAGTACTAAATTGAATTGCAAAATCAGTGTGATTAACTGTATGTCTATACCCCTGGAAGATGGTAAGATTGATTGAACCACGCAATCCGGCTTGGTGACCTTTAATTGTTTTCAAGATGAATTTGAGGTCTGTTAACATTTCTTTTGTATGTCTATTTTGAGAAGGGCTAGCACCTACTAAACAACTTGAACCCGGCAGAGCTAGTACAATAATTCAGCACAATCAACCAGATCAGGGTAGAGAAATTTGGAATTGAGATAGGGCAATTTATTTAACTATAGGTTTATGGATCCAAGTCAAGAAACACTACTTTATTTGGTCATGTTATGTTTGAATGCTTAACCAGTAATGTTAACTCAAAGCTGAGGTCAAAATAGATTTTAGATAACCTTGACCAATGTTAACTCAAAGCTGAGGTTAAAATAGACTATATAGTAGCAGTCAAACTGTAAGAGCAAGAAATGCAGCAGGAAATATGTCTTGTGAAGAAAATTCTTAAAGGCAGACAAAACACAAACATCGCACCTCACTTAATGTACATAGCCGTCTGCCAGACAAAATAAAGCATGCCCTTATCTTTTTTATAGTAGTCTGATTCTCCGGTTAGTCAATCACTTGTGCTTGATCAAGCAGACAGGAGTGCAAACTTCTTGATATCTCTCCCATCTTCATTCTTCTCGTACTTGGCTGATGTGAGTTTCAATTTTCTTTTCCTACAGAGTCAGCATACAGGTCAATAATTTTAATACTGTTAAGATGGTGCATTAAAGGTAAACTGGAAAGGATTTTAGCAAAAGACTTGCAGAGAATTTTTATAACAAGTGATTATGATCTCACATAAAAGCTATGAAAACTTGGACATAGGAGCTACAAAAAGAGAGGAGGGTGGAAGGTGACTGAGTGATACTTCAACCAGGTAAATTGCACACAAATTCAGGGTTAAGGACTCAAGTTGTATCACATTCCGTGAATAAGCATAACAGATGGGCTCAGAAAGATTTGACTGAAAGATTCTAGTACAGGATAGGCTGAACAATTATGCACTGAGCAACAAAATGGGAATTTGAGTCAGCAGCACCTGTGTATGCAAACCTGACGAGGCAGCTTACTATGCATGAATCTGGCATGTTATTTTTTTAAATAAAATCAATTACGTAGCTAGCTAAGAAATCGTTACATGTAATTGTAAGGACTTAAAATAACATGCTACATTTGTCTTCAAAGTCGATGCAATTTAAGTAATTAATTCATTCAAATGTAAGCAGATTAGACTAGCACAATAATAGAATTGCCTTTGATTATAATACTAATACAAAAGTCAGCAGAGTAGTGAAGAATCAGTAGCCAGGAGCTGGAAACCTTTGAAAAAGTGAACACAATCTGCCTTAAAATTTGAATCAACACAATATCTTCATCATTATAATCAAATATGATACGCTGGAGATCCTAAATGACAGAGATAATCTAAGAGTGGCACAATCCTCACCCATAAATGACACTTCTAATCCAGCAGTAAAAAATCAACTCTAACAGCAAGGGCTTTAGAGCTTTCTAGAGCGGCTATGACACTAAAAGCTCGAAAAATGGCATTCCCTCCACCCATGGTGTATATACCTGGGTAATTGTACAGTCTAGCATCAATATAATAATACAGTTGTGAAACAATTGACAAACACTAGCTATTTTGTTCAACCTCTTATTTAGTTATTTACCCTTATGCAAAATTTTTATTATTATAATTTATACTAGTAATGCAATGATGCTAAAATGCAAGGCTAATAATAATGCAAACAGAATATTCAAAGCACATTACCTGTCTGCAAGTCTCCAAAAGCTCATACAAAGCAGACCACATGGCCTCTTTGCAGAATGAATGTCTGACGGCTAAGAAAATTAGAGTCTTTGATGATTGAGTAACTTGGTTTGAAATTTGCGTTCATCAGTGTCATTCTATCTGGACTAAGACCTCTCTCAAGCATTTCATCGTACATTTTGAAAGCTTCAGGCAAATTACCCTCTTTAAAATACCCAGCAACCAAGCATTATACATAAAGGGAGTTAGGAGTCGCTCCCTTTTCTTCTATCTCTTTGAAAATTCACGTTAAATTTTCAGCTTGCCTTTCTTACAGGCCATTCACAAAGCTGTGTAAGTGATTTCATCAGGCATGATGCCTTTCTTCATTTCAGCATAGAATTCTAAAGCTGATTGCAGTTTATCTTCCTTCAGAAGTCATCGATTAAAGAGGTGTATGTTTCAAATCACAAAATATCCCTTGTTCAGTCATTTTTCTTATGGAACAGAGCCGCTTCAATGTTATTTATATTCCTATAACCACTGATCATGGAAGTATAAATATATATGTTTGAGACAACCCATAACTTCACACAATTCATCAAAAAGTTTATGCACTCTTCATGTCCCTTCTTTTGCAAAAAGCGTCAATAAGTGTGCCATATGCGGTAATATCAATTTAAGACCCTTGCTTATCATCTCATTGAGCATTTTCAGAGCAGATCAAAATTATTGCTCTTGCAGCATGCATTAATTAAATTGTGTATGTGAAAACAGTTGGGTAGACTCCTTTCTCACTCATCTCATTATAAACAGAAAATGCAAAATCAGATCACCTTCCTTCAATAATCCATCAATTACCATATTGTAAGTCATGCAAAAAAAAAAAAAGATGGTATGAAATTATCTTTCTTTATAAAATTCTCTAGTAGATTCTTCGCATACGATGTTTGCCATGTTGCACAATCCATCAACAGTATTGTAAATTGTAATCTGTACAATCAATCCTTAGGTCCACCCTCTCATCAAACAACTTCAGAGCTTTCTCTACCTCACTTTCTTGAAATACCTGCTTATTAATACTGTTTAGGTAGTAGTGGGTTTCACACCCCTTAACAATCATCTCAACATACATGCTATGTGCCATAGTGGCGTTTCTATCTTACAATAACCATAAATTAGGTTGTTATAATTAACTACATTAGGTCTGACTCCACATCTTATCATCTTATCCCAAATCTTTTGAGCTTCATCCACCTTGTCATCTCTACAAAGCCAAGATACTAATATGTTAAATGTGGATCCATCAGCCATAAACCAGATTCAGCCGCCTTGTTAATCATCTCATAGGCTTTCTCCAATAACAGCCTTTCAGAAACCCTTTATCATTGAATTAATATTATAGACACTTTGGGTTTGATACCCTTTTCAGTCATTTGATCAAAAAGCTCAGATGCTTTCTCCATATCCCCCTTCCTTACAACATCCATCAGTATAACTGAATAAGTGGCTATATTTGGTGTAAGTTCATGCTCAGCTAATTCAGCAAACAGACCTAAAGCACTCTCCCTAATAATCACCCGATAAACAATACCCCTTTATCAAGTTCATAGCAACCACAAATTCATCGGTTTTCACAGACATCATTTCATCTTTCCAGTCTCAAAGCTTCCATCAAATTCTGCTGCTTCACACACGCGATAATAACGGTGTAGGTACCAGCAGAAGACACAAAACCTTTATTTCATCTCCATCAACAACTCACAAGCTGCATTACAATCAGGTTTTACTAACAACTCCAACAGCACAACTAAACACGCCCGGTTCTAATTCCAGCCCCTTATCCTTTGCTGCCTAAAATATCCCGCTCCGCCTCCTCAGCTCATCTTTCAAACAGCCCTCATCATCAAACGAACAGTAGCACTATTCTCAACAAAACCTCTACAACACACCTTATCATATTACAACTCTAGCTTCTTTAATCATATTCTTCTGAAACACTAAGACTTGGATAATATCATTCACATAAACACCACACATCAATGTTATTATAAAGCATCCAATTAAAACAACTAATTGCATCATCAACTCTGTCAGTTGCTGCAAGATATCAAATAGCAAATGGATAAAATCTCGCGAATCACAATTCGAAATCAAACCTTTGGAGCAATCATTGAGGCTGCGGTCATTTGTAGTAGCATCAGGAAATTTCGGGGTTGAAACCAGATTGCTGCTGATTACTGAAACACAAGCCCTAAAATTATTACCCAATAAACAGAAAGACTCAACCCCATCACCAAAACCTGGTTGATTTTCAGCCCATTGAAGTATGATGATAAGAAGGTTGTCCTTTGAGCTCAATAACTCATTTGCATCATAAAACTGAAAGATTTGTGGGATTTTGATGATTTTTGTGAGATGGGTATTTGGGAATCAGCCTCGTTTAAATCATTTGAGATTTGGGATTGGTTTAATGATGAAAGATTCAAGAGACTGGTTCCCTGGGTAATTAAGGGTTCTGAAGAAGATAAGAAGGTTGAAGATGAAGATAGTGGTTCGTGGATTAGTGAAAGAACGCCATAAAATTTGATGAAATTTGCAGAGATTTTAAATTTCATCTTTTGGCAGGATTTAGCGACAACGGGGAAATTAGTGGTGTTTGAACCTAGTTTTGTTAGAGAAACAAAAATTGGAAACAGTGCTGCTCTGTAGTGAGTTGCGAAGAGGAAGTAATGGACCGGAGTCTACCCGAAAAGGAAGAGATGGACCGATTCTGTTATCCTGCTTTATATTTTTGGTTTTATGCTCTTTTTACGAGCATATGATACGGAGATCGATAGTAAGCATGGGCAACAGGCCGTGCTGTGCCTGCTTAGTCTTAGCCACGTGGTGGGCGATATCTTGCGTCTTGAATCTTTACGTTGCTTGTGCCGTAGCGTTACGCCGAGCCGTCTTGTCTTGAACATGAATGGCCAGATTTTTTTTTTTTTTTTTTAATGTGCATCGTGAAATACGATATATATATTTTTAGTCTGAGCAATTTTATATAAATAAATAATGTTATTAAGTTTTAGTGACTATTTTAATTAAAGTTTATAAGTATCTATGTAATTATGTTTTAAGTTTATATAAATAATTTTATTAATTTAGCAAGCAAACAATCACGTATAATAAAAAAAGCTACCGAGTGCGATAAGCGAAAACACAAAAAAATTGATCGATAACCTTTTTATTATATGTCGGTGAGTCGGTGCGATAAATCGGTAATACGAAAAATTAATGATAATTGAAAATCGTCTGATAATTGGTGAATAAGCTTGTTTTATTAAACGGAATAGAAAATTTGTACAATAGTATGTAAATATGTAATTATTATTCTTCGTCTTAGTCTTGTTAAAGTTCTTATTAATTGAATTGATTGAAGCCCGTAGAAACCAAGCGATTCGGCTTCCGTCGTAGTTGTACATAAAAATCGATCTCTTGAGCAGTTTCGTCATTGATCCTTGAAGCAAACACACATTTCAAATGTTTAGGGTTTTTAGTCTTGTTCTTATCGTCAAAACCGTCAGGCACTAAAAGCTGGATTCGAGCAACGGAAGACATTATGCAAAAATATCACGAGCCATTGCGCTAGAACAAGGATGTTTGTACTTGTAGTTCACAATCAAGATATGAGGCCAAACTTATAATAATAGCCGTCGTCCACGCGCTTTCATGAAATTAAATGAAACTGAGTATATTTGCGGCTTCAGAAACATGGCTGAGTTGCCAGGATGATGATTCGATCTTGGCTTCCCAAAGCAGGGTTAGCGACTTGTGCAAAATAAACCTTTAGGCCCCCTTTCTTTACGTTTTGGATGTTTTGAGAAGGCGGAGAATCGAAACTAGATTAAGTATTTAGATTCCATAAACTCATGTAAAGAGAAAAAATCATTAGTCACCTTAGTTTTAAAAGTTTCAGCATCATCTTGTAAATTCATGTTAGTATAGTAAATATCAATTAATGTAAAGTGCCATCATACTTTTGTCTAGGATTAAAATGAAGCAAAATTATAAATCATAGGAATATTAGTATATATTCGCCATTTTCCTCGATAGATGGAAGATATCCATCATAATCATGTTGTGAAGCGGTGTTCGGCCAAAGAAGTTGTGCAATTAAAGCATGCTTTGCTAAAGAAAAGAATGGGAACAAGGATTATATACGGAAGAAAAACAATGTGTCATGCCATATAATAATTGTAAAAGCGCATATATTTTCACAATTAGCCCAATCATCTTCTAGTAGAAACGAATTGTGGAAATTTTTGATTATAAAAGCGTTAAATGAACATATCTTCGGTGCATCTTCTTCAAATAGGTAGGTTCTTTGGTTGGACAATCGATTGCTATTTTTATAGCGCTTCTTGCTTTGTTCTTAAAACCCTTTTCCAGATTCTTGATATCTCAAGTTAGAGCTATTAATAGACACTTAATAGCATTTCTAATTTTGTGATAAAATGGACCCAATTGCTTGTGTTCCATCCTTACGCACATAAGTTAGAGTATGACAAATGCATCTAATATGAAGTGCATCTCCATCTAATATAACATCGGTTGTCAGAGGTTTAACCAAATTTATAAATGTCGCAAGAGTTGCATTATCAAAAGTCATAGACATGATTTTATCCACTAAGACTATATCATCCAATAAGCTTACATTAAATGATGAGAAATGTTAAGGCGGTATATGAGGTATTGAGCATTTTAAAAAATGAATGATGCGTTTTGTAACAATAATCATTATCAACCAATGACAAGTAACACATAAATAGTGAACTCATTAACCACTTATCCATTACATCGGCAGTTAAAGCTACTAGGAAGATTACTACAGGAATTCATTTTCTAATTTCAATTTATATAAAAAATTCTTCATAGCGTAGCGAAACGAAGCGGTTTCGGGAGATTTTGTGTTAGGGTTGGAGTGCTTTCAGTTAATGTAATTATTAAAGGCATCATTTCGGCGAGAAAAAAAGAAAGGTTATTCGACGCATGGACTATCATTCTCTGATTCATCTCTATCTATCTCTTAACTATAAGTGAAGCCATGTGGATGGAAATGAGAAATTTTAGGTTGGGATGGATCACTAGATGGTTCACTACATTTTTTGCATGTCTCTTAAGGTGTCACGTCAGCGCTTAGTTTCGAGGCGGAGAAAAATTTTCTACTGACTCCGGCCAGCGGAACTCAGTCAAAAAATGCTTTACTTAATTGATAATGTGCCCAGGCTTCACTCTTTCGATTAGGAATTAGGAATTAGGGTTAGGGTTTAGTTTAGGGTTTAGGGTTTAGGGTTTAGGGTTTGGGTTTAGGTTTGGGTTGGATTGGGTTTAAGGGTTTAGGGTTTAGGGTTTAGGAGTTTAGGGTTTAGGGTTTAGGGTTTAGGGTTTGAGGTTTAGGGTTTAGGGTTTAGGGTTTGGGGTTTAGGGTTTAGGGTTTAGGGTTTAGGGTTTAGGGTTTAGAGGTTTGGGGTTTAGGGTTTAGGGTTTAGGGTTTAGGGTTTGGGGTTTACGGAGTTTAGGGTTTAGGGTTTAGGTTTAGGGTTTAGGGTTTAGGGTTTAGGAGTTTAGGGTTTAGGGTTGGGTTTAGGGTTTAGGGTTTAGGGTTTAGGGTTTAGGGTTTAGGGTTTAGGGTTTGAGTTTAGGGTTTAGGGTTTAGGGTTTGGGTTTAGGAGTTTAGGGTTTAGGGTTTAGGGTTTGGGTTTAGGGTTTAGGGTTTAGGGTTTAGGGTTTAGGTTTAGGGTTTAGGGTTTAGGGTTTAGGGTTTAGGGTTTAGGGTTTAGGGTTTAGGGTTTAGGGTTTAGGGTTTAGGGTTTAGGGTTTTAGGGTTTTTAGGGTTTAGGGTTTAGGTTTAGGGTTTGAGTTTAGGGTTTAGGGTTTAGGGTTTAGGGTTTTGGGTTTGGGTTTAGGTTTAGGGTTTAGGGTTTAGGGTTTAGGGTTTAGGGTTTAGGGTTTAGGGTTTAGGTTTAGGGTTTGGGTTTTTAGGGGTTTAGGGTTTAGGGTTTGGGGTTTAGGGTTTAGGGTTTAGGGTTTAGGAGGTTGGGTTTAGGTTTTGGGGTTTGAGGTTTGGGGTTTAGGGTTTAGGGTTTAGGGTTTAGGGTTTAGGTTTGGGTTTAGGGTTTAGGGTTTAGGAGTTTAGGGTTTGAGTTTAGGTTTAGGGTTTAGGGTTTGGGTTTGGGTTTAGGTTTAGGAGTTTAGGGTTTGGGTTTAGGTTGGGATTCTAGTTGGAGTTTAGTTGGGTTGGGTTGGGTTAGTTGGGATTAAAGTTTAAGGTTTTAGGGTTTAGTGAGTTTAGGGTTTAGGGTTTAGGGTTTTAGGGTTTAGGGTTTAGGGTTTGGGTTTAGGGTTTAGGAGTTTTTAGGGTTTAGGGTTTAGGGTTTAGGGTTTGAGTTTAGGGTTTGGGGAGTTTAGGGTTTAGGGTTTAGGGTTTAGGGTTTAGGGTTTAGGGTTTGAGGTTTAGGGTTTAGGGTTTAGGGTTTAGGAGTTTAGGGTTTAGGGTTTTAGGGTTTAGGGTTTAGTTTAGGGTTTAGGGTTTAGGGTTTAGGGTTTAGGGTTTAGGGTTTTAGGGTTTAGGGTTTAGGGTTTAGGGTTTAGGGTTTAGGGTTTAGGGTTTAGGTTTTAGGGTTTAGGGTTTAGGGTTTAGGAGTTTAGGGTTTAGGGTTTAAGGGTTTAGGGTTTAGGGTTTAGTTTAGGGTTTTAGGGTTTTTAGGGTTTAGGGTTTAGGTTTAGGGTTTAGGGTTTAGGGTTTAGGGTTTTAGGGTTTAGGGTTTAGGGTTTAGGGTTTGGGTTTAGGGTTTAGAGGTTTAGGGTTTAGGGTTTAGGGTTTTAGGGTTTTAGGGTTTAGGGTTTAGGGTTTAGGGTTTAGGGTTTAGGGTTTAGGGTTTGGGGTTTAGGTTTAGGGTTTAGGGTTTAGGGTTTAGGGTTTAGGGTTTAGGGTTTAGGGTTTAGGGTTTAGGGTTTAGGGTTTAGGGTTTAGGGTTTAGGGTTTAGGGTTTAGGGTTTAGGGTTTAGGGTTTAGGAGTTTAGGGATTAGGGTTTAGGGTTTAGGGTTTAGGGTTTAGGGTTTAGGGTTAGGGTTCGAGGTTTAGGGTTGGGATTTAGGGTTTGGGGTTTAGGGTTTTTAGGGTTTAGGGTTTAGGAGGTTTAGGGTTTAGGGTTTAGGGTTTAGAGGTTTAGGGTTTAGGTTGGGTTGGGTTTAGGGTTTAGGGTTTAGGTTAGGGTTTAGGGTTTAGGGTTTAGGGTTTAGGGTTGGGTTTAGGGTTTAGGGTTTAGTTTAGGGTTTAGGGTTTAGGGTTTAGGGTTTAGGGTTTAGTTTAGGGTTGGGTTGAGGTTTAGGGTTTGGGTTTAGGGTTTAGGGTTTAGGGTTTAGGGTTTAGGGTTTAGGGTTTAGAGGTTTAGGGTTTAGGGTTTAGGGTTTAGGGTTTAGGGTTTAGGGTTTAGGGTTTAGGGTTTAGGGTTTAGGGTTTAGGGTTTAGGAGTTTAGGGGTTTTAGGGTTTAGGGTTTAGGGTTTAGGGTTTAGGGTTTAGGGTTTAGGGTTTAGGGTTTAGGGTTTAGGGTTTAGGGTTTAGGGTTTAGGGTTTAGGGTTTAGGGTTTAGGTTTAGGGTTTAGGGTTTAGGGTTTAGGGTTTAGGGTTTAGGTTAGGTTTAGGTTTAGGGTTTAGGTTTAGGGTTTAGGGTTTAGGGTTTAGGGTTTAGGGTTTAGGGTTTAGGAGTTTAGGGTTTAGGGTTTAGGGTTTAGAGGTTTAGGGTTTAGGTTTAGGGTTTAGGGTTTAGGGTTTAGGGTTTAGGGTTTAGGGTTTAGGGTTTAGGGGTTAGGGTTTAGGGTTTAGGGGTTAGGGTTTAGGGTTTAGGGTTTAGGGTTTAGGGTTTAGGGTTTAGGGTTTAGGAGTTTAGGGTTTAGGGTTTAGGGTTTAGGGTTTAGGGTTTAGGGTTTAGGGTTTAGGGTTTAGGGTTTAGGGTTTACGGGTTTAGGGTTTAGGGTTTAGGGTTTAGGGTTTAGGGTTTAGGGTTTAGGGTTTAGGGTTTAGGGTTTAGGGTTTAGGGTTTAGGGTTTAGGGTTTAGGGTTTAGGGTTTCGGGGTTTAGGGTTTAGGGTTTAGGGTTTAGGGTTTAGGGTTTAGGGTTTAGGGTTTAGGGTTTGGGAGTTTAGGGTTTAGGGTTTAGGGTTTAGGGTTTAGGGTTTAGGGTTTAGGGTTTAGGGTTTAGGGTTTAGGGTTTAGGGTTTAGGGTTTAGGGTTTAGGGTTTAGGGTTTAGGGTTTAGGGTTTAGGGTTTAGGGTTTTTAGGGTTTAGGGTTTAGGGTTTAGGGTTTTAGGGTTTAGGGTTTTTAGGGTTTAGGGTTTAGGGTTTAGGGTTTAGGAGGTTTAGGGTTTAAGGGTTTAGGGTTTAGGGTTTAGGGTTTTTAGGGTTTAGGGTTTAGGGTTTTAGGGTTTAGGGTTTAGGGTTTAGGGTTTAGGGTTTAGGGTTTAGGGTTTAGGGTTTAGGGTTTAGGAGTTTAGGGTTTAGGGTTTAGGGTTTAGGGTTTAGGGTTTAGGGTTTAGGGTTTAGGGTTTAGGGTTTTAGGGTTTAGGGTTTAGGGTTTAGGGTTTAGGGTTTAGGGTTTAGGGTTTAGGGTTTAGGGTTTAGGAGTTTAGGGTTTAGGGTTTAGGGTTTAGGGTTTAGGGTTTAGGGTTTAGGGTTTAGGGTTTAGGGTTTAGGGTTTAGGTTTAGGGTTTAGGGTTTAGGGTTTAGAGGTTTTTAGGGTTTAGGGTTTTGGGTTTAGGAGTTTAGGGTTTAGGGTTTAGGGTTTAGGGTTTAGGGTTTAGGGTTTAGGGTTTAGGGTTTGGGTTTGAGGTTTAGGGTTTAGGGTTTAGGGTTTAGGGTTTAGGGTTTAGGGTTTAGGGTTTAGGAGTTTAGGGTTTAGGTTTAGGGTTTAGGGTTTAGGGTTTAGGGTTTAGGGTTTAGGGTTTAGGGTTTAGGGTTTAGGGTTTAGGAGTTTAGGGTTTAGGGTTTAGGGTTTAGGGTTTAGGGTTTAAGGGTTTAGGGTTTAGGGTTTAGGGTTTAGGGTTTAGGGTTTAGGGTTTAGGGTTTAGGGTTTAGGGTTTAGGGTTTAGGGTTTAGGGTTTAGGGTTTAGGGTTTAGGGTTTAGGGTTTAGGGTTTAGGGTTTAGGGTTTAGGGTTTAGGGTTTAAGGGTTTAGGGTTTAGGTTTTAGGGTTTAGGGTTTAGGGTTTAGGGTTTAGGGTTTAGGGTTTAGGGTTTAGGGTTTAAGGTTTAGGGTTTAGGGTTTAGGGTTTAGGAGTTTAGGGTTTAGGGTTTTAGGTTTAGGGTTTTGAGGTTTAGGGTTAGGGTTTAGGGTTTAGGGTTAGGGTTTAGGGTTTAGGGTTTAGGGTTTAGGGTTTAGGGTTTAGGGTTTAGGGTTTTAGGGTTTGGGTTTAGGGTTTAGGGTTTAGGGTTTTTGAGGTTTAGGAGTTTAGGGTTTAGGGTTTAGGGTTTAGAGGTTTAGGGTTTGAGGTTTTTAGGGTTTAGGGTTTAGGGTTTAGGGTTTAGGGTTTAGGGTTTAGGGTTTAGGGTTTAGGGTTTAGGAGTTTGGGGTTTAGGGTTTAGGGTTTAGGGTTTTAGGGGTTTAGGGTTTAGGGTTTAGGGTTTAGGGTTTAGGGTTTAGGGTTTTAGGGTTTAGGGTTTAGGGTTTAGGGTTTAGGGTTTAGGGTTTAGGGTTTGAGGTTTTTAGGGTTTAGGGTTTAGGGTTTAGGGTTTAGGGTTTAGGGTTTGGGGGTTTAGGGTTTAGGGTTTAGGGTTTAGGGTTTAGGGTTTAGGGTTTAGGGTTTAGGGTTTAGGGTTTAGGGTTTAGGGTTTAGGGTTTAGGGTTTAGGGTTTTAGGGTTTAGGGTTTAGGGTTTAGGGTTTAGGGTTTAGGGTTTAGGGTTTAGGGTTTAGGGTTTGGGTTTAGGGTTTAGGGTTTAGGGTTTAGGGTTTTTAGGGTTTAGGGTTTAGGGTTTAGGGTTTAGGGTTTAGGTTTAGGGTTTAGGGTTTAAGTTTAGGGTTTAGGGTTTAGGGTTTAGGGTTTAGGGTTTTAGGGTTTAGGGTTTAGGGTTTAGGAGTTTAGGGTTTAGGGTTTAGGGTTTAGGGTTTAGGGTTTAGGGTTTAGGGTTTAGTTTAGGGTTTAGGGTTTAGGGTTTAGGGTTTAGGGTTTAGGGTTTAGGGTTTAGGGTTAGGGTTGGGTTGGGTTAGGGTTTAGGGTTTAGGGTTGGGTTTAGGGTTTAGGGTTTAGGGTTTAGGGTTTAGGGTTTGGGTTTTTAGGGTTTAGGGTTTAGGGTTTAGGGTTTAGGGTTTAGGGTTTAGGGTTTAGGGTTTAGGGTTTAGGGTTTAGGGTTTAGAGGTTTAGGGTTTAGGGTTTAGGGTTTAGGGTTTAGGGTTTAGGGGTTTAGGAGTTTTTAGGGTTTAGGGTTTAGGGTTTAGGGTTTAGGGTTTAGGGTTTAGGGTTTAGGGTTTAGGAGTTTAGGGGTTTAGGGTTTAGGGTTTAGGGTTTAGGGTTTAGGGTTTAGGGTTTAGGGTTTTTAGGGTTTAGGGTTAGGGTTTAGGGTTTTTAGGGTTTAGGGTTTAGGGTTTAGGAGTTTGAGGTTTAGGGTTTAGGGTTTAGAGGTTTAGGGTTTAGGGTTTAGGGTTTAGGGTTTGGGTTTAGGGTTTAGGGTTTGGGTTTAGGGTTTAGGGTTTGGGTTTAGGGTTTAGGGTTTAGGGTTTAGGGTTTAGGGTTTAGGGTTTAGGGTTTAGGTTTGGGTTTAGGGTTGCAGGGTTTAGGGTTTAGGGTTTGGGGTTTAGGGTTTAGGAGTTTAGGGTTTAGGGTTTAGGGTTTGGAGTTTAGGGTTTAGGGTTTAGGGTTTAGGGTTTAGGGTTTAGGGTTTAGGGTTTAGGGTTTAGGGTTTAGGGTTTAGGGTTTAGGGTTTTGGGGTTTAGGGTTTAGGGTTTAGGGTTTAGGGTTTTTAGGGTTTAGGGTTTAGGGTTTAGGGTTTTTAGGGTTTAGGGTTTAGGGTTTGGGTTTAGGTTTAGGGTTTGGGTTTAGGGGTTTAGGGTTTAGGGTTCTGGGGTTCGGGGTTTAGGGTTTAGGGTTTAGGGTTTAGGGTTTAGGGTTTAGGGTTTAGGGTTTAGGGTTTAGGGTTTAGGGTTTAGGGTTTAGGGTTTAGGAGCAATACGTAA

**Data S2.** Example of block structure of terminal telomeric array where clusters of TTTAGGG motifs were interspersed with clusters of TTTAAAA motifs.

*C. acuminatum*, accession 429-3, read 5413 (20152 bp). Refer to Figure 1B.

TRF analysis:

Sequence: 429-3_5413

Parameters: 2 7 7 80 10 50 500

Length: 20152

This is table 1 of 1 (11 repeats found)

| Indices | Period Size | Copy Number | Consensus Size | Percent Matches | Percent Indels | Score | A | C | G | T | Entropy (0-2) |
| --- | --- | --- | --- | --- | --- | --- | --- | --- | --- | --- | --- |
| 9196--9261 | 14 | 4.7 | 14 | 72 | 12 | 55 | 3 | 7 | 46 | 42 | 1.47 |
| 9249--19151 | 7 | 1406.0 | 7 | 85 | 11 | 5236 | 23 | 0 | 32 | 43 | 1.57 |
| 9992--10274 | 86 | 3.3 | 84 | 87 | 11 | 366 | 55 | 1 | 1 | 42 | 1.13 |
| 14950--14983 | 16 | 2.1 | 16 | 94 | 5 | 59 | 44 | 0 | 2 | 52 | 1.16 |
| 16605--16648 | 18 | 2.6 | 18 | 86 | 13 | 67 | 27 | 0 | 38 | 34 | 1.57 |
| 19113--19158 | 21 | 2.2 | 21 | 84 | 0 | 56 | 23 | 0 | 30 | 45 | 1.53 |
| 19147--19355 | 7 | 29.7 | 7 | 84 | 15 | 249 | 55 | 0 | 0 | 43 | 1.06 |
| 19190--19233 | 9 | 5.2 | 9 | 86 | 8 | 58 | 63 | 0 | 0 | 36 | 0.95 |
| 19351--19618 | 7 | 38.9 | 7 | 89 | 5 | 422 | 12 | 0 | 44 | 43 | 1.42 |
| 19614--20152 | 7 | 76.3 | 7 | 83 | 13 | 632 | 55 | 0 | 2 | 41 | 1.17 |
| 19831--19869 | 10 | 3.8 | 10 | 93 | 6 | 69 | 51 | 0 | 17 | 30 | 1.46 |

Consensus pattern (7 bp):

TTTAGGG

Consensus pattern (7 bp):

TTTAAAA

Sequence:

>429-3_read_5413

CACTGAGCTCTCCATTGAAACATAACGAGCTTTCTCTCCTCCATTGAAATAGTTTAAATGGTACAGACCAAAATTTGTTTATTCATTTTTTAATCTAAATTATTGAATATCAAGTTCTAATCTAAGGTTTGTTAAGTTTATTGTCGTTTTTCATTAATTTGATACTTCGTATGCATGCATCCCAGATCAATTGTAGCGTATATTACGAAATTGACGTTTAAATTTACTGCAATTGCAAATTTAAATTCCATAAATTTCCTACTTTTTCACTTTCTCTTGTCTTTAGTTTGATTGAATTTTACAACCCTCGATTAATTATCAAATTGGAGACAAATTAAAATTAATTAGCAGGGAGTTTGATTAGGGAGACACCATTGTTGTCTATATGGATACATGAGATATAGCCAAAGAGAACTACTCAAAGAAAAAGAAACGTAATTGCTAGCCAAATCAATTAGTGTTTGCATTACAAGGAGGCTGTTCCTTTAGGAAGAGGCCAGTGGATGAAAATATATGGGAAGAATTCGAGTCCATGATAAAAAACCTCCTCTCTTATCCTGGCAATATTTCAATTCAATTACCCCCATAATATATTTATGTTTGTTCTTTATTTTTGACTCAATTTGATGTTTTTGTATAGAATTTATGTTTTTGTTCTTTACTGTTTTAGCTCATTTTTGATGTTTTGGAGGTGAAGTTTGATCAATAGATTGAACTTAAAAGCAATAGAAGAAATCACGGTTTGCAATTCTCAGATATCCAGCATGCTCAAGCTCTACAAAACCTTTAAAGTTTAACACTAAAATGGAAATTATTATACTTGTTCAAAGTAAAATTGGTGTAATTATGTATGATTTATTTTATTTTATTTTGTGAATATGGACTAATTACGGTTTTGACTTTCCCTTTTCTCTATATATTATGTTCATCTTGACCATGAAATAATATCTTTGGTAAACAAGTGAATATAATGACTTACCCAGGATACCTCAATGGAAAAGGTGATATATAAAAGCTCTGCCTCATCGGAACTTTAAGCTCTGATCTCTCTCCATCAATAATTTACGTTCTAGCTGTACGCCGTTATAGACGTTTAGTTTTATAAGTCTTTAGTTTTTTTTTTATAGGAGAATAAGTCTTTTTAGTTTTGTAAAACATTATTTTGAAAAAGTATGTTTGACAATAAGGTTAAGAGTCAATTATAAAAATAATTTATTAATTTTGCAAATCTTCTAGCAATGTTGGTCACTTGTGGATTGGCTTTCTTATACATATATATATTCGTAGTTTGATTATAATTTTGCATTTTTATTCAATTGAAATGTAACATGAATATATATGTGAATATTCAAGCTCATGTATATGTCAAGCACATCAGTCATTATATGTTCGCTAATGTTCTTTTGTGAAGCACTTACTATACTTTTTCAACATTTAGTATGCATAATCATATATATATAACTTTCACTTAAAATTACTCCAATTAAGATTATCGTAAATTTTCAAAAATAAAAACAATTTATCACGAACCTTAATAGTGTTACAAATCGGCTAACTGTTTCAACTGGCATCTATCATGTGTCAAATCCCTAGTCAAAAAAGAAAGGTTACTTACGACACATTAAACTAATATATATATATGCTAAAATACCCATATGACTCTAATTTAAAAGCAGAGATATATTAACTGATTAATTAGGTACATTAATCGGTCAAAAAAGAAAAATTACGACCATTAAACTAATATACTATTCTCGTACTAAAATACCCATATTCTAATTAACAATGAAATTAATTAACGAGTGAATTAATAATTTTGTTATTATTCAAGAAGATTATGACATTAAGGTAATGTGCAAAATGTATATCTATAGTATGCTTAAAACATTATACAAATATAAAGTAGATCTGATGCATACGTTGAAGTTTGGTTATTTCACAATTACATTCTTTATAAAAATGTTGGGTTCGAACAGTGAGTTAACGGCGTCACCTAAGTCTTCAAACTTTACTAAGTCTCACCTATATCCACCACCTAGTAACTTTGGATCTTCCCACTAACCTTCAATCTCCTCTCACCACATTGTTGACCTTTCTCACTTTCTCATTACATTCCATTAAAACAACTCAAATCTCACAGAGCCCCATCAAACATCTTATTCAGTTAAGCACTTCTCTCTTCCTCTTAATTTCTCCTCAAAAACAGAGGAGAAGGAATTAGAGCAGGAGAAAACAATGTTGGGTATAAAGATACATACGCCAATCTTAGACCCAAGTTCTTGGAGTTTAATTGTTGTTAGTATTCTTCCTCTTCAGCTGCTGCAATTGAGGCTTTAGAACTAGTAAAAGTATTACAATGATTGGACTCATCCTGGTCAATCTATTTATGATTCTCCTGTTTTAGACCTTCTCTTTCATAAGGGTAGAATTTGATGCATTCTCTTTTTATTAATCTAATTTTTTCATGTTCTACTTTGTTTTGCTGGTAGTGATGTAGACAGATTTTGATTCAAGTCTTGTGTACAATCCCAGAGACTTACTAACTAAGATAGTTAAATTATTGACATTTATTATTCATTTAATTGCTTATGGAATATGTCAGATTTTTTGATTGAATCTGTAGTTCTGGTCTTGTGTTGAATTTTGTGATATTCTAAATTCTAATTTATAGTTAAAAAAAGGTTGAGTTTTGGTGTAATAGTAGCACTTCTATTTCTGTTTTACCCCTTAAATTTTCTAGCTTTTTATATGTTTTCTGAAATTAATTTCTTTAATACTTGATATGGTAGCATTGAATTTTTAATTCCAAACTTAGGTGCTAGACTAAGTTTATATGTACTTATGCTTGTGGCAATCAGTTTGACTTGGTTTGAGTCTATCTAAAGAAGCTACAATTCCTGCCAATCTAAAGTTTGGCCAGTCCTGCTATGTAAAAATTCAGCCTCCAAATTGGTTTGGTCACATAGAGCATTACAGCTTGAGTTTGATTGCGGCCTGCCCGTTAATAGGGATTTTCATGAAAACACGAACAAGGTGATACATTGCTAAATATTCTCCCACACTCAGTGAACACCGTAACTAATATAACTGGGATCCCTTGTAGTCATCAAGTCCAACAGCCAAACCGTCCATAGATATGTTATTCTTAAATGAGGCATATTCACTGACTATTATGAAAAGACCCTGTGTTAGTGGTTCAAGAATTAAGACAAGGAAAGTGGGCATTTGATGATGAATTTGGTTGTACTATATATTTCAATCAACCTATATTGATACTTTAATAGATTTTCTCTGAATGTAATGAACTTATAGTGATCTTTCTTGGTGTAAAAAATTCTTAAACTTTCTATGTGTCATATGTCGATGTTTTTGTCGTATTGCATCCATGATTAAGTCTAATACATTTTATAGCTATTCCTCTAGGGCTGCTACCATTTATTGTTCTCTTTGTATAGTTACTAAATCTAAGAGTGATATTTAGCGGATTCGTTCCACATTTTATTAGCCTTGTTATTTATTCCAGTTGTCACCATGACCAGTTCTCTTCACTGAACGGAGAATGTTGTTATATGGAAGTATTGCAGGCTGAGTTCATGGGCATACCCCACAATTTAGAGAGTTCAACAATGCACTACTCTCCTCCATAAGGCTGGAGGCTGCAGTGAGGATTGTTCTTATTGCCCACAATCCTCTCGATATGGCACTTTCGTGCAGGCTCAAAGGTATTGAATAAGGACCCACAGTCATTTATGAGCAGCAAAGAAGGTGTTTTTCCATCATCAAATGCTGAGATAGACAATGAATCCTCAGCCCAAACTACACATGAAAATTAAATATGATATTGAATATAATTTCTAATAAACATTGGTAACAGTTCAGATCTTTGTCTTAAAGATGAAGAGGAATAACATGATTTAGTGCAAATTGATGTATCCAATTAATTGTGTTAGAATGTAACTTCAATGACTATCAATTGGTTTTATGTAGGCCATATATTTGTATTGATTTGTGTGCTTTGTTTCTTCCCTGTGTTTGTTGTTTAATGGTATCTTTTCCTATACTGGCAGGCAAAAGAGGCTGGAAATACAGCGATTTTGCATAGGAGAGCTGCTTGGAGGGACACTGTAGGGCGGAAGACTAATTTCCAGCAAATTCTTGATTATGTGAAAGAAATAAGGTGATGAAGAATTTTCATTTTGATTTTATGTACTTTAGTTTTTTTTTTTTCCCTATTTTCTTTCTTTTCAATTATTGAGCCAAATTACAGACTTTAATGAAAGCATTTTCTTTTTATTTATTGAGTAAATTCTTGACTTTGTGAAAACATTTTTCTACCATTGACTATTCCCATTTGAAGGTTCACAAATCAGTTAGCTTCTTCTTGATTAAGGTCGCAATACTCATATATTGATGATAATTTTTAGTTTTTAAATCTGAGAAAAATGTCTTTGGCAATGCATTTCCTATCTATCAAGTCTTTGGTTATCCTTTCTCTTCTCCTCTCTTTTTGAACCTCTATCTATTCTTGTCCCACAGTGTTGTAAATTTTCAAGCTATCATCTTATTCTTATTATTGTTTTTATGTTATGAGCTTATCACTTGTAGTTGGTTTAAAAGAAGAAATCATATCATTGAGCTCTTAATCATAGCTAGATGCAAAAATTAAAGGTACTTTTATATCAACTGTTCTTTCACCTTTTTATTTCTTGTTTCATTAATTTCCTAGGATTGATTGGATTAGCCTATGTAACCACTGAGCTTCTTGAATTATTATTATAACCATTGAATTTTGCAGATTTGGTAGGTTGTCTTTTACGTAGATAATATCCTTTACCCCTTTCTTTTTTTCCCCCTTAGGGCATGCTTGGATTGGGAAATATATTATGGGGAGTAATAAAAGAAGTCGAAACTAAAGCAAAATGAATGTGAAAATAGGAAAAGGAAAGGAGATAGTGATGGTGTGATTGTAAGAAGATTATGTAGTGATAGAAGATATGGTGTGAAATTGAAGAAAAGAGGAATCAAATACCCTCATTTTAGGGGTATGGATTACGCTAGGGAGGGTGGGTAACTATTACCCTCATTATGAGTAATAATTCCTCCATCTCTCTCTTTTCCTCATGGCACAACTAGTTTATTTCATTTGTGGCTTTTATGTCTCCTACAACACCACCTCTAATTACTTTTGTTTTTCAAGTTTGACTTTATTACCCCCTTAATGTTTTACCCCAATCCAAAGCATGCCCTTAGATGAGATGCATTTCAAATATCTATTCAAATCATTTGGTCTCTTTCTTTATATAATGCTGCATACATATTTACCATGCTGCATACATATAATCTTTGCCCATGTGCATTAGGAATGTAGACCATTTAAGTGATTCAATTACGTGCCATGGGCTGAAGCCTTTCCAACACATTAAAATGTCTGATATTACAGGAGAATATGGCAGGGTGAGAGAGAAGGAGAGAAATACTAAGAAATTATTGGAAATAATGAGTCTAATTATTGTATAAGGAATTTCTGGATTATTTTCTTGACACCACCTTATAGCTAAAGACAAAAAAGAGATTGATGATTGTCCTAAAAGCTTTGTGGTTAATATCTGTCATTGTCATCGTAGTACTTTAGGTAAAGCTAATCTTCTTACTAAAGTTTATTTATAACAGAAATGGAGCATAGGTCTATTTTTAGCAGGCATGCTGGAGAAGCAGCAAGCAGAGGACTTGAAGAAGGCAGGTCTTACAGCATGCAATCATAATCTTGATACCTCAAGGGTTTATCCGAATATTACAACTCGATCTTATGATGAGCGACTACCAAACTCTTGAGTTTGTATGCAGTGATGCAAGGGATCAATGTCTGCTGTGGTAGGTTGGCCCACAAGAGACAGGTTGGAATTTTTATGTGTAGTACATTTCTGTTTTCTGTGCTACCATATCCAGTTGTGAATTGTGATAACCTAAGGCATTTAACTGGTTGCATTTAAAAGTTTTAACCACATTAAAATCGCCAACAACTACCATTTATATTTGTCAGATTATTACCACCTCTTATTTATTTTAATAATTTTAGTCTCAAACCACCTTATAAAAACTTTGTTAAAAGTAACTCCACCATTAAAATATCACCGGATCGATTCTGACACCCGAGGTTTTACAGGCCAATGAATTATGCAGTATTCTATTGGCCTCTTTAATGTATGTAGTTGGGTATTTCCTCATTTCAACTTGGTTGACTTTTATATTAGTTTGCCACAGGAGAATAATTGGACTTGGAGAAACAGCCGAGGATCAGTAGGTCTATTACATACACTAGCTACTTTGCCTTCACACCCAGAGAAGCGTTCCAATTAATGCACTTATAGCTGTAAAGGCACTCTCTTGAAGATCAGAAGGTAAATATAATCCCTAAAATTACCTGAAAATATAAATCTTGGCTCAATTCTTTGTTAGATTATGCACGTTTACATCACACTTTTGTGACATGAGTGCCTGGTTGTGGAGATAACTGATTGTTTGCTTGTGGTCAAAAAGTTTGTCACTCTGTTATAAATATTATTTTCCATGTATGGTGATACTTTTCATATTAGTATCAAATTATCCTATACTACTATTGTTTGTATGATTCTTGCCATTAAAGCTGTCTTAGTGTGTATTAGTGAAATAATGTTTCCTTTTTCTGAAATGGAGTATCTGGACTTTGGGCGCTTGTGGAATGAGAACTTTCCCGTGTTATATCTTAGCCTTATGGACCCTTTGCTTGGCAAAATTCTGAACTTTTGTTGTAGTCAGACATTCTCATCATATTTTGGTAGCGCGATAGTTTGATACTAATGTCATCCTTCCTGTGAGCAGCCGTTTTCATCCCTTTTTGGAAGCTCTGAACTGTGTAATTCGCTGAAAAATACATGTAAAGAAGCATTTCTTAGTCTTTATAATCCATTAAACTCTGATGTACTGAGAATGCATGTTCCTAGTTAAAGTTCATATATTTTTTTTTTGTGACAATACATCGACTTATTTAAACATTCTTATGTTCACTTCAAATCCTTCATGGTGAAATATGGGAGATGATTCGGATGATAGCAACTGCTTTCGCATAGTGATGCGAGTGCAATGGTCAGGTTATCAGCTGGGAGGACCCGGTTCTCAATGCCGAACAAACTTATGTTTTTCTTGCTGGTGCAAATTCGATCTTCACGGTGAGAAGCTATTGATAACTGCTAATAGTAACGACTTTGATGCTGATCAAAAAAATGTTCGAGTTCTTGGTTTAATTCTAAACCTCCGAGTCTAGATGATACACAAACAGAAGCTTGTGAAGCAGTTGCATCTAGTTCAGGTTAAGACTCTAATCCAATGTCTTTAGTTTGCTTGGATACTGCTTGGCTATATTAGTCGAGTTAAATGTGGAATTTACATATTATTTTTGAATTCTGCACCTCCTTCCCCGTTTTATCCCAAAGTCGTGTTTGGATGCATGTAATTGTTTTGAATGTATTAATTTTAATCTAATTGTAAACAATTTGAAGCAAGATTATTGGCCCCTATGACAGGATTACCCCAGTCCCCCAACAAAAGGCTAGGTTGGGGATGATATCCTCTATTATATCTTATAGTTTTTAACTAGGCTTTGCACACACATAAAGGAAATGTCGCGATCTTTACTTGACAGCATTTATAAAAAGAGAGGAAAGTTCACTTTAAGGAATACTTATCTTATATTCTTTATTTAAAGCATATGGCCAGCAGAAGTACAAAACTCTTGTATGGCTCGTACTTATTTTAATGTTATTATATAAAATATAAACCCACGTTTAAGTATCGCGCTAAAGGGTGTGTTGCGTCTAAAAACTGAGGAAGTAGGTTTTATGATATTATCTATAGTTTGGATGATCTCCTCTGATGGATTCTACACGATATCATTTATAAATTCGGATGATATCATTTATTAGAAATTTAATGATATCATCCATTGCTTATATTATACTCCTCCTTGGATTCCCAAAATGATAGTCGCATGGCTTATTTTGGTCGAATTGATGGTCAGTTGTTTTTCAAATCTTAATTAATATCAATTTTTTTTTTTTATCAAAATTCCCTCCAATTACAACAATATTTATTTTAAATTTAACTTTCTCCAAATGAAATTGTGTTTTCGTGTACTTTTTTTTTTATTTTCTACTGCTATGTTACATACTACGATGTAAATTTATGCTCGATGTAATTTTCACTTCACTTTGATTTAAGTTCTTACAGTGCGATGATTTTGCCAGTAAGGGACTATCGTTTGGGGACGGAGGGAGTATACGTTTATAGACTGAAGATAATATATATCTCTTAATAGCTATATGATATCATTTGCAAATTGTTTAGAAACATGTAATACTTTGTTGGATTATATCCACAATAGAGGATATCGTCGGTAATAAGAATATCGTTAGTTAGTAGAGGTTGTCGCCCAGACTTATAAAGTTATATCGACTTATTATAGATGATATAATTAAGAAATAAAATTTATTTAAAAAAATAATTAAAAGGCCAATAATGATGGTCACCATTGAAGGTCAGTGCCAGATCCCCTTAGATTATAACATAACATGTTCATTTCTCGACAAAAACATGCATTTGTTATCAAAGCGGCGCTCACAATTCAACATACAATTATATAGAACAATCCCACTTTAAACAGTGCCACATCACGAAAGCTACAAATGTCTCGATTTTTAGGCTATTTTGAAAAGTAAAGAATCTTAATCAATTATCAAAGCCAATTCAAGCACAAATTATGGACAAGTATGACCAACAATATTTATTTTTCATTATCATATCTATATCTTCTTCAAAAATGACATGAATTATAATTCAAATAAAATAGAAATAATTTAAGCAGTTAGAGAGTAAGAAATAGGGGTGAGTTAAAAGGTGAGAAGGTGAAGAAAGGGAGGTAGTTAGTGAAGTATTGTTGAGTAGTTTGTAGTGATAAGATGTGAAGTTTGTAATGTCGTGCATAAAACAATAACATATTTAGGTTTAGAAATTCGGGGTTTAGGGGTTTGAATTTCGGGTTTCGGGTTTGGGGTTCGGGGTTTGGGTTGGGGTTGAGGGAGTTCGGGTTAGAGTTTTAAGGTTTAGGTTGAGGGTTTTAGTTACAGTTTAGTTTAGGATTTAGTTTAGGTTTTGGGTTGGGTTTTTCGGGTTTCGGGTTTCGGGTTTGGGTTTGAGTTTGGGGGGTTCGGGATTCGGGGTTTGGGGTTTAGGGGTTAGGGGTTTTCGGGGTTTAGGGTTTAGTTTAGGGGTTGGGGTTAGGGTTTGAGGTTAGAGGTTGAGGTTTAGGAGTTTAGGAGTTTGAGTTTGGGTTTAGGTTTAGGGTTTAGGGTTTAGGGTTTAGGGTTTGGGTTTTAAGGGTTTAGGGTTCCAGTTCAGTTTGGGTTTAGGGTTTAGGGTTTAGGTTTTAGGGTTTAGGGTTTAGGGTTAGGGTTTAGGGTTTAGGGTTTGGGTTTAGGGTTTAGGGTTTAGGTTTAGAGTTTAGGGTTTAGGGTTTAGGGGTTTTAGGGGGTTGGGTTTAGGGTTTAGGGTTTAGGGTTTTAGGGTTTAGGGTTTGAGTTTAGGGTTTAGGGTTTAGGGTTTAGGGTTTAGGGTTTAGGGTTTAGGGTTTAGGGTTTAGGGTTTAGAGGTTTAGGGTTTAGGGTTTTAGGGTTTAGGGTTTAGGGTTTAGGTTAGGGTTTAGGGTTTAGGGTTAGGGTTTAGGGTTTAGGGTTTAGGGTTTAGGGGTTTAGGGTTGGGTTTAGGGTTTGAGTTTTAGGGTTTAGGGTTTAGGGTTTAGGGTTTTAGGGTTTAGGGTTTAGGGTTTAGGGTTTAGGTTTCTAGGGTTGGGGTTTAGGGTTTAGGGTTTAGGGTTTAGGGTTTAGGGTTTAGGGTTTAGGGTTTAGGGTTTAGGGTTTAGGGTTTAGGGTTGGGGTTTAGGGTTTAGGGTTTAAATTTAGGGTTTAAAATTTAAAATTTAAAATTTAAAATTTAAAATTTAAAATTTACAAAATTTAATTTAAAATTTAAATTTAAAATAAATTTGAAATTTAAAATTTAAATTTAAAATTTAAAATTTAAAATTTAAAATTTAAAATTTAAAAATTTAAAATTTAAAATTTAAAGAATTTAAATTTAAAATTTAAAATTAAAATTTAAAATTTAAAAATTTAAAATTTAAAATTTACAAGAATTCAATTTAAAATTTAAATTTAAAAATTTAAAATTTAAATTTAAAAATTTAAGGTTTAGGTTTAGGTTTAGGGTTTTAGGGTTGGGTTGGGGTTTAGGGTTCAGTTTTAGGGTTTGAGTTTAGAGTTTAGGGTTTAGTTTAGGGTTTAGTTTAGGGTTTAGGGTTTGGAGTTTAGGGGTTTAGGGTTTAGGATTTAGTTTAGGTTTGGGTTGGGTTTAGGGTTTGGGTTTAGGTTTTGGGTTTAGGGTTTAGGGTTTAGGGTTGGGTTTAGGGTTTAGGGTTGGGTTTAGGGTTTGAGTTTGGGTTTAGGGTTTAGGTTTAGGGTTTAGGGTTTAGGGTTTAGGGTTTAGGGTTTAGGTTTAGGGTTTGGGTTTAGGGTTTAGGGTTTGGGTTGGGTTAGGGTTTAGGTTTTAGGGTTTTGGGGTTTAGGGTTTAGGGTGGGTTTAGGGTTTAGTTTTTAGGGTTTGGAGTTTAGGGTTTAGGTTAGGGTTTGGGTTTAGGGTTTAGGGTTTAGGGTTTGGAGTTTGGGTTAGGTTTTGGGTTTAGGGTTTAGGTTTTAGGGTTTAGGGTTTAGGGTTTAGGTTTAGGGTTTAGGAGGTTTGGAGTTTAGGGTTTAGGGTTTTTAGGGTTGTTTTTAGGTTTTTTGGGGTTTGGGGTTGTTTAGGGTTTAGGGTTTAGGGTTTAGGGTTTAGGGTTTAGGGTTTAGGGTTTTTAGGTTGGGTTTAGGTTTGGTTTGGGTTTAGGGTTTAAGGGGTTTAGGGTTTAGGGTTTAGGGTTTAGGGTTTTAGGGTTTAGGGTTTGGGTTTAGGGTTTAGGGTTTAGGGTTTAGGGTTTAGGTTTGGGGTTTAGGAGTTTAGGGTTTTAGGGTTTAGGGTTTAGTTTGGGTTTAGGTTTAGGGTTTAGGGTTTAAGTTTAGTTGGGTTTTGGGTTTAGGGTTTAGGGTTTAGGGTTTAGTTTAGGTTGGGGTTTAGGGTTTAGGGTTTGAAGGTTTAGGGTTTAGGGTTTAGGGTTTAGGGTTTAGGGTTTTGGTTTAGGGTTTAGGGTTTAGGGTTTGGGGTTTAGGGTTGGGTTTGGGTTTGGGGTTTTTAGGGTTTAGGGTTGGGTTTAGGGTTTAGGGTTTAGGGTTTAGGGTTTAGGGTTTAGGGTTTAGGGTTTAGGGTTTAGAATTTAAAATTTAAAAATTTAAAAATTTAAAAATTTAATTTAAAATTTAAAAATTTAAAATTTAAAAATTTAAAATTTAAAGAATTTAAAATTTAAAATTTAAAATTTAAAATTTAAAGAATTTAAAGAATTTAAAATTTAAAATTTAAAATTTAAAATTTAAAATTTAAAAATTTAAAAATTTAAAATTTTAAAATTTAAAATTTAAAATTTAAAATTTAAAATTTAAAATTTAAAGAATTTACAAAAATTTAAAATTTAAAATTTAAAATTTAAAATTTAAAATTTAAAAATTTGGGTTTAGGGTTTAGGGTTTAGGTTTAGGGTTTTAGGGTTTAGGGTTTAGGGTTTTAGGGTTTGAAAGTTTAGGGTTTAGGGTTTAGGGTTAGGGTTTTAGGGTTTAGGGTTTAGGGTTTAGGTTTTGGGTTTCAGGTTTAGGGTTTAGGGTTTAGGGTTTGGGTTTAGGGTTTAGGGTTTAGGGTTTAGGGTTTAGGGTTTAGGGTTTAGGGTTTAGGGTTTAGGGTTTAGGGTTTAGGGTTTAGGGTTTAGGGTTTGGGGTTTAGGGTTTAGGGTTTAGGGTTTAGGGTTTAGGGTTTAGGGTTTAGGTTTAGGGTTTAGGGTTTGGGGTTTGGGTTTAGGGTTTTTAGGGTTTAGGGTTTTTAGGGTTTAGGGTTGGGTTTAGGGTTTAGGGTTTAGGGTTTGGGGTTTAGGGTTTAGGGTTTTGGGGTTTAGGGTTTAGGGTTTAGGGTTTAGGGTTTAGGGTTTGGGGTTTTAGGGTTTAGGGTTTAGGGTTTAGGGTTTAGGGTTTTTAGGGTTTAGGGTTAGGGTTTAGGGTTTAGGAGTTTAGGGTTTAGGGTTTGGGGTTTAGGGTTTAGGAGTTTGAGTTTTAGGGTTTAGGGTTTAGGGTTTGGAGTTTAGGTTGGGTTTGGGGTTTGGGTTTGGGTTTAGGGTTTAGGGTTTAGGGTTTAGGGTTTAGGGTTTAGGGTTTAGGGTTTTTAGGGTTTAGGGTTTAGGGTTTAGGAGTTTGGGGTTTAGGGTTTAGGGTTTAGGGTTTAGGGTTTAGGGTTTAGGGTTTAGGGTTTAGGGTTTAGGGTTTAGGGTTTAGGGTTTAGGGTTTAGGGTTTAGGGTTTAGAGGTTTAGGGTTTAGGGTTTAGGGTTTAGGGTTTAAAATTTAAATTTAAAGAAAATTTAAAGAATTTAAAAATTTAAAAATTTCAAAATTTAAAAGTTTAAGAAATTTAAAATTTAAAAATTTAAAATTTAAAATTTAAAATTTAAATTTAAAATTTAAAATTTAAAATTTAAAATTTAAATTTAAAATTTAATTTAAAATTTAAATTTAAAAATTTAAATTTAAAATTTAAAAATTTAAAATTTAAAATTTAAGAATTTTAATTTAAAATTTAAAATTTAAAATTTAAAATTTAAAATTTAAAATTTAAAATTTAAAAATTTAGTTTAGGGTTTAGGGTTTGGGTTTAGGGTTTGGGGTTTGAGGTTTAGGGTTGGGGTTGGGGTTTAGGGTTTAGGGTTTAGGGTTTAGGGTTTAGGGTTAGGGTTTAGGGTTTAGGAGTTTAGGGTTTAGGGTTTAGGGTTTAGGGTTTAGGGTTTAGGAGTTTAGGGTTTAGGGTTTAGGGTTTAGGGTTTAGGGTTTAGGGTTTAGGGTTTAGGGTTTAGGGTTTAGGGTTTTTAGGAGTTTAGGGTTTTTAGGGGTTTAGGAGTTTAGGGGTTTAGGGTTTAGGGGTTTTGAGGGTTTAGGGTTTAGGGTTTTAGGGTTTAGGGTTTAAGTTTTAGGGTTTTTAGGGTTTAGGGTTTAGGGTTTAAGGGTTTTAGGGTTTAGGGTTTAGGGTTTAGGGTTTGGGGTTTAGGGTTTAGGGTTTAGGGTTTAGGGTTTAGGGTTTAGGGTTTAGGAGGTTTAGGGTTTGGGGTTTAGGGTTTAGGGTTTAGGGTTTAGGGTTTAGGGTTTAGGGTTTAGGGTTTAGGGTTTAGGGTTTAGGGTTTAGGGTTAAAGGTTTAGGGTTTAGGGTTTAGGGTTTAGGTTTGGGTTTAGGGTTTGAGTTTAGGGTTGGGTTTAGGGTTTGGAGTTTAGGGTTTAGGGTTTAGGTTTAGGGTTTAGGGTTTAGGGTTTAGGGTTTAGGGTTGGAGTTTAGGGTTTAGGTTTAGGGCCACTAAAATTTAGGTTTAAAATTTAAAATTTAAAATTTAAAAATTTAAAAATTTAAAATTTAAAATTTAAAAATTTAAAATTTAATTTAAATTTAATTTAAAAATTTGTTAAAATTTAAAATTTAAATTTAAAATTTAAAAATTTAAAATTTAAATTTAAAATTTAAAATTTAAGAATTTAAAAATTTAAAATTTAAAATTTAAAATTTAAAAATTTAAATTTAAAATTTAAAATTTAAAATTTAAAATTTAAAATTTAAAATTTAAATTTAAAATTTAATTTAAAATTTAAAATTTAATTTAAAAATTTAAAATTTAAAAATTTAAAATTTAAAATTAAAAATTTAAAATTTAAAATTTAAATTTAAAATTTTAAAATTTAAAATTTAAAATTTAAAATTTAAAATTTAAAATTTAAAATTTAAAAATTAAATTTAAGAATTTAAATTTAAAAATTTAAAATTTAAAATTTAAGAATTTAAAATTTAAAATTTAAAATTTAAATTTAAAATTTAAAATTTAAAATTTAAAATTTAAAAATTTAAAATTTAAAATTTAAAATTTAAAAATTTAAAATTTAAAAATTTAAAAATTTAAAATTTAAAATTTAAAATTTAAAATTTAAAATTTAAATTTAAAATTTAAAATTTAAAATTTAAAAATTTAAAATTTAAAAATTTCACAAAATTTAAAATTTAAAATTTAAAATTTAAAATTTAAAATTTAAAATTTAAAAATTTAAAATTTAAAATTTAAAATTTAAAAATTTAAAATTTAAAATTTAATTTAAATTTAAGAATTTAAAATTTAAAATTTAAATTTAAATTTAAAATTTAAAAATTTAAAATTTAAAATTTAAAATTTAAAATTTAAAATTTAGGTTTAGGGTTTAGGGTTTAGGGTTTTTAGGGTTTAGGGTTTAAGTTGGGTTTAGGGTTTAGGGTTTAGGGTTTAGGGTTTAGGGTTTAGGGTTGCAGGGTTTTTAGGGTTTAGGGTTTGAGGTTTAGGGTTTAAAGTTTAAGGGTTTTTAGTTTTAGTTTGGGTTTAGGGTTTAGGGGTTTAGGGTTTTAGGGTTAGGGTTTAGGAGTTTAGGGTTTAGGGTTTAGGGTTTAGGGTTTAGGGTTTAGGAGTTTAGGAGTTTTTAGGGTTTAGGGTTTAGGTTTAGGGTTTAGGGTTTAGGGGTTTAGGGTTTAGGGTTTTAGGGTTGGGTTTAGGGTTTAGGGTTTTTAGGGTTTAGGGTTTTTAGGGTTTAGGGTTTAGGGTTTAGGGTTTAGGTTTAGGGTTTGGGGTTTTAGGGTTTAGGGTTTAGGGTTTAGGGTTTGGGGTTTAGGGTTTAGGGTTTAGGGTTTAGGGTTTAGGGTTTAGGGTTTAGGGTTTAGGGTTTAGGGTTTAGAGAATTTAAAGTTTAAATTTATTTAAATTTAAATTTAAAATTTAAAAATTTAAAAATTTAAAATTTAAAATTTAAAATTTAAATTTAAAATTTAAAATTTAAAAATTTAAAATTTAAAATTTAAAAATTTAATTTAAAATTTTAAAATTTAAAATTTAAAGAATTTAAAATTTAAAATTTAAAATTTAAAATTTAAAATTTAAAATTTAAAATTTAAAGAAATTTAAAATTTAAAATTAAAAATTTAAAATTTAAAATTTAAAATTTAAAATTTAATTTAAAATTTAAATTTAAAATTTAAGGGTTTAGGAGTTTGAGTTTAGGAGTTTAGGGTTTAGGGTTTAGGGTTTAGGGTTAGGGTTTAGGGTTTGGGGTTTTGGGTTTAGGGTTTAGGGTTTTAGGGTTTAGGGTTTAGGGTTTAGGGTTTAGGGTTTAGGGTTTGGGGTTGGGTTTAGGGTTGGGGGTTTAGGGTTTAGGGTTTAGGGTTTGGGGTTTAGGGTTTAGGGTTTAGGGTTTAGGGTTCAGGTTTAGGGTTTAGGTTTAGGGTTTAGGGTTTAGGGTTTAGGGTTTAGGGTTTTAGGGTTTAGGGTTTAGGGTTTAGGGTTTAGGGTTTAGGGTTTAGGGTTTAGGGTTTAGGGTTTAGGGTTTAGGGTTTTGAGGTTTAGGGTTTAGGGTTTAGGGGTTTAGGGTTTAGGGTTTAGGGTTTGGGGTTTTGGGTTTTAGGGTTTAGGGTTTAGGGTTTAGGGTTTAGGGTTTAGGGTTTAGGGTTTGGGGTTTTAGGGTTTAGGGGTTTAGGGTTTAGGGTTTTAGGGTTTAGGGTTTAGGGTTTAGGGTTTAGGGTTTAGGGTTTAGGGTTTAGGAGTTTAGGGTTTAGGGTTTAGGGTTTAGGGTTTAGGGTTTAGGGTTTAGGGTTTAGGGTTTTTAGGGTTTAGGGTTTAGGGTTTAGGGTTTAGGGGTTTAGGGTTTTAGGGTTTTAGGGTTTAGGGTTTAGGGTTTAGGGTTTAGGGTTTAGGGTTTAGGGTTTAGGGTTTAGGGTTTTTAGGGTTTAGGGTTTAGGTTTAGGGTTTAGGGTTTAGGGTTTAGGGTTTAGGGTTTAGGGTTTAGGGTTTAGGGTTTAGGGTTTAGGGTTTAGGGTTTAGGGTTTAGGGTTTAGGGTTTAGGGTTGGTTTTTAGGGTTTAGGGTTTGGGTTTAGGGTTTAGGGTTTTTGGGGGTTTAGGTTTAGGGTTTAGGGTTAGGGTTTAGGGTTTTGGGGTTTGGGGTTTTTAGGGTTTAGGGTTTAGGGTTTAGGGTTTAGGGTTTAGGGTTTAGGGTTTGAGGTTTAGGGTTTAGGGTTTTAGGGTTTAGGGTTTAGGGTTTAGGGTTTAAGGGTTTAAGGGGTTTCAGGTTTAAAGTTCAGAGGGTTTAGGTTTAGGGTTTGGTTTAGGGTTTAGGGTTTAGGGTTTAGGGTTTGGGTTTAGGGTTTAGGGTTTAGGGTTTAGGGTTTAGGGTTTAGGGTTTAGGGTTTAGGGTTTAGGGTTTAGGGTTTAGGGTTTAGGGTTTAGGGTTTAGGGTTGGGGTTTAGGGTTTTTAGGGTTTAGGGTTTAGGGTTTAAGTTTTAGGGTTTAGGGTTTAGGGTTTAGGGTTTAGGGTTTGGGTTTAGGGTTTAGGTTTGGAGTTTGAGGTTTAGGGTTTAAAGGTTTAAGGGGTTTAAGAGGTTTAAGGTTTAAAATTTAAAATTTAAAATTTAAAAATTTAAAAATTTAAAATTTAAAATTTAAAAATTTAATTTAAAATTTAAATTTAATTTAAAATTTAAAATTTTAAAATTTAAATTTAAAATTTAAGAAATTTTAAAATTTAAAATTTAAAATTTAAATTTAAAATTTAAAATTTAAAGAATTTAAAATTTAAATTTAAATTTAAATTTAATTTAAAAATTTAAAATTTAAATTTAAAAATTTAAATTTGAATTTAAAATTTAAAATTTAAAGTTAGTTTGAGTTTAGGGTTTAGATTTTAAGTTTAGGGTTTAGGTTAGTTTAGGGTTTAGGGTTTAGGGTTTGGGGTTTGGGGTTTAGGGTTTGGGTTTAGGGTTTAGGGTTTAGGGTTTGGGGTTTTTAGGGTTTAGGGTTTAGGAGTTTAGGGTTTAGGGTTCCCAGGGTTTAGGGTTTAGGGTTTAGGGTTTAGGGTTTAGGGTTTAGGGTTTAGGGTTTAGGTTTAGTTGGGATTTTGGGGTTTGGGGTTTAGGGTTTAGGGTTTAGGTTTAGGGTTTAGGGTTTAGGGTTTAGGGTTTAGGTTTAGGGTTTTGAGGTTTAGGGTTTAGGGGTTTAGGGTTTAGGGTTTAGTTAGGGTTTAGGGTTTAGGGTTTAGAGTTTAGGGTTTAGGGTTTAGGGTTTAGGGTTGGGTTTAGGGTTGGAGTTTAGGGTTTGGGTTTAGTTTTACAGGTTTAGGGTTTAGGGTTTAAGGTTTAGGAGTTTTTTTAGGGTTTAGAGTTTGGGTTTGAGGTTTAGGAGTTTAGGGTTTAGGGTTTAGGGTTTAGGGTTTGGGTTTAGGGTTTAGGGTTTAGGGTTGGGATTTAGGGTTTAGGTTTAGGGTTTTGGTTTAGGGGTTGGGTTTAGGGTTTAGGGTTTAGGGTTTAGGGTTTAGGGTTTAGGGTTTAGGGTTTAGGGTTTAGGGTTTAGGGTTTTTAGGGTTTAGGGTTTAAGAGGTTTACGGGTTTAGGTTTAGGAGTTTAGGTTTTTAGGTTTAGGGTTTTTAGGGTTTAGGGTTTAGGGTTTAGGGTTTTAGGGTTTAGGGTTTTAGGGTTAGGGTTTTTAGGGTTTTAGGGTTTGGGTTTAGGGTTTTTAGGGTTTAGGGTTTAGGGTTTAGGGTTTAGGGTTTAGGGTTTAGGGTTTAGGGTTTAAGGGAGTTTAGGGTTTTTAGGGTTTAGGGTTTAGGGTTGGGGTTTAGGGTTTAAGGGTTAGGGTTTAGGGTTTAGGGTTTAGGAGTTTAGGGTTTAGGGTTTTAGGGTTTAGGGTTTAGGGTTTAGGGTTTAGGGTTTTTAGGGTTTAGTTTAGGGTTTAGGGTTTAGGGTTTAGGGTTTAGGGGTTTAGGGTTTGGGTTTGGGTTTGGGTTTAAGGTTTAGGGTTTAGGGTTTAGGGTTTAGGGTTTAGGGTTTAGGAGTTTTAAGGGTTTAGGGTTTGAGGTTTAGGGTTTTTAGGGGTTTAGGGTTTTTAGGGTTTAGGGTTTAGGGTTTTTAGGGTTTAGGGTTGTTTAGGGTTCAGGGTTTAGGGTTTAGGGTTTAGGGTTTAGGGTTTAGGGTTTAGGGTTTAGGGTTTAGGGTTTAGGAGTTTAGGGTTTAGGGTTTGAGTTTAGGTTAGGGTTAGGAGGGTTTGGGTTGGGTTTGGGTTTAGGGTTTAGGGTTTTTAGGGTTTAGGGTTTAGGGTTTAGGGTTTAGGGTTTAGGGTTTGGGGTTTAGGGTTTAGGGTTTAGGGTTTGGGGTTTAGGAGTTTAGGGTTTAGGGTTTAGGGTTTAGGGTTTTTAGGGTTTGGGTTTAGGGTTTAGGGTTTAGGGTTTAGGGTTGGGGTTTAGGGTTTAGGGTTTGGGTTTAGGAGTTTTGGGGTTTAGGGTTTAGGGTTTAGGGTTTAGAGTTTAGGGTTAGGGTTTAGGGTTTAGGGTTTTAGGGTTTTAGGGTTTAGGGTTTAGGGTTAGGGTTTTTAGGGTTTTTAGGGTTTAGGAGTTTAGGGTTTAGGGTTTAGGGTTTAGGGTTTAGGGTTTAGGGTTTAGGGTTTAGGGTTAGGGTTTAGGGTTTGGGGTTTAGGGTTTAGGGTTTAGGGTTTGGGGTTTAGGGTTTAGGGTTTAAGGGTTTAGGGTTTAGGGTTTAGGGTTTTTAGGTTTAGGGTTTAGGGTTTAGGGTTTAGGGTTTAGGGTTTAGGGTTTAGGGTTTAGGGTTTAGGGTTTAGGGTTTAGGGTTTAGGGTTTAGGGTTTAGGGTTTAGGGTTTTTAGGGTTTAGGTTTGGGTTTAGTTTCAGGGTTTAGGGTTTTGCAGGGTTTAGGGTTTAGGGTTTAGGGTTTAGGGTTTAGGGGTTTGGGGTTTTTGGGTTTAGGGTTTTAGGGTTTAGGGTTTTAGGGTTTAGGGTTTAGGGTTTGGGTTTAGGGTTTAGGGTTTAGGGTTTAGGAGTTTAGGGTTTGATTTTAGGGTTTAGGGTTTAGGGTTTAGGGTTTAGGGTTTAGGGTTTAGGATTTAAAATTTAAATTTAAAATTTAAAATTTAAATTAAAATTTAAAATTTAAAAATTTAAAATTTAAAAATTTTAAAAAATTTAAAATTTAAAATTTAAAATTTAAAATTTAAAATTTAAATTTAATTTAAATTTAAATTTAAAATTTAAGAATTAAATTTAAATTAATTTAAAATTTAAAATTTAAAATTTAAAAATTTAAAAGTTTAGGGTTTGGGTTTAGGGTTTGGGTTTAGGGGTTTAAGGTTTAGGGTTTTAGGGTTTAGGGTTTAGGGTTTAGGGTTTAGGGTTTAGGGTTTAGGGTTTAGGGTTTGGGGTTTAGGGTTTAGGGTTTAGGGTTTAGGGTTTAGGGTTGGGGTTTAGGGTTTAGGGTTGGGGTTTAGGGTTTAGGGTTTAGGGTTTAGGGTTTAGGGTTTAGGGTTTAGGGTTTAGGGTTTGGGGTTTGGGTTTAGGGTTTGAGTTTAGGGTTTAGAATTTAAAATTAAGTTTAAAATTTAAAGTTTAAAATTTAAAAAATTAAAATTTAAAATTTAAAAATTTAGATAAGAATTTAAAATTTAAAATTTAAATTTAAGATTTAAATTTAAAAATTTAAAATTTAAAATTTAAAATTTAAAATTTAAAATTTAAATTTAAGATTTAAAATTTAAAAATTTAAATTTAAAATTTAAATTTAAAGTTTAAAATTTAAAGGAATTTAAAGGAGATTTAAAGGAATTTAAAATTTAAAATTTAAAGATTTAAAATTTAAAATTTAAAATTTAAAAATTTAAAATTTAAAAATTTAAAATTTAAAATTTAAAAATTTAAAATTACAAAAATTTAAAAATTTAAAATTTAAAATTAAAATTTAAAATTTAATTTAAAAATTTAAAATTTAAAATTTAAATTTAAATTTAAAATTTAAATTTAAAATTTAAAATTTAAAATTTAAAATTTAATTTAAAATTTAAAATTTAAAAATTTAAAATTTAAAAATTTAATTTAAAATTTAAAATTTAAAATT

**Data S3** Example of telomere sequences array of *Arabidopsis*-type in the interstitial position

*C. acuminatum*, accession 429-3, read 1954 (24378 bp). Refer to Figure 1C.

TRF analysis:

Sequence: 429-3_read_1954

Parameters: 2 7 7 80 10 50 500

Length: 24378

| Indices | Period Size | Copy Number | Consensus Size | Percent Matches | Percent Indels | Score | A | C | G | T | Entropy (0-2) |
| --- | --- | --- | --- | --- | --- | --- | --- | --- | --- | --- | --- |
| 14401--14431 | 14 | 2.2 | 14 | 94 | 0 | 53 | 16 | 0 | 41 | 41 | 1.48 |
| 14481--15578 | 7 | 156.7 | 7 | 81 | 8 | 1207 | 18 | 0 | 42 | 38 | 1.53 |
| 20109--20139 | 12 | 2.6 | 12 | 100 | 0 | 62 | 22 | 35 | 9 | 32 | 1.87 |

Consensus pattern (7 bp):

TTTAGGG

Sequence:

>429-3_read_1954

ACTCATTATACTATTATAATGTGGCAGCATTAATGGTTAGTACCATACACCTCTAGATGGTGATGTAGTAATTAGCCTCCTAGAAGACTAAAAAAAATCAACCAAAAATCAGGCATTTTTGAATGGTTTATACTTTTTAGCTCAAAACATGTGACATAAAAAAATGTCTTAGTATAACATAGACACTTTGTTTGGACATTGCTAATATGGACAATAAAAGAAATTAATATTATAAATTTTATAAAGAATAAAATTATAATTAAATGTAATTAGTTAGATGGCAACAAACAAAAATTGACACGTAATCATAAATTTGTATTTTAACCTTAGCTTCTACCTATTTGACAAATCTTCTCTTCCAATCATAATGGATTTGTTTACCGAATTAAAATAAAGTAATCATATAATTGTATATAATAAGGTTACAAGAAAAACAAAAACTTTGAATTTGAGCGTATTCGTGTGTTCCATGCTGCTCCTGCTCCCCTCGGTTGTGGGGTAATCATAGATCAATCACATAAAGACTGCAATTTCATAAACATTATCTTGTTTGGAAATGTTAATACAATGTATATTCGACCAAAATAATGTTAATTAATGGTTGATTTTACACCAAACTATTGTTTTATAATCTACTTGTCTTGATGCCTAAGTTATTGTGGTATGACTTGTGACTGATTGTATAGTTTCTTCACACATAATAATAGTTTTGATTGGTTTCTTAAAGAGTGAAATCGGAAATTCTAATGAAATTTCCCTTTGTCGGAGGAGAAGTTTACATTATTTGAGGAAACAGGTAAGATTTTAGGAATAACTAAACACGCCATTTAGAGCTACAGAGAGACATTGATGTATGTTAGATTGAGTTTGACAGTACAACAAAAATTAATTGATGTATGCCTTATAATAAAAACATCAACAATGCTAACAAAAAGAATGACATAGTTTCCAAAAGAAAGAAAAGACATTGCTAGTAAAATTTTCATAACTACTAATAAATCAGCAAAGAGTAACATCAGCTATTTTCATATTTTTTCCAGAATAAGGTAAGCAATCAGACTTAGCATAGAGTAAATAAATAAAACTGTGTGCAATGTTTTAATTGCTGTAAACGGCACCATTAAGCAAGGTCTGGCATGATAGCAAGAACATAGTTCACAGAATCACCATGGGTCAGGTTAGACACTTGACACAAGAGGACGAATAGACAGGTTTGTTATTCAATCAGAAATACTCCAGGATCTCATTGTTACATCATTCCCTGAAGAGTGAAAAGCCAAAGAAAGTAACTCTGCAAAACATAGACATAAATTTGAACTTAAATGAACTCCAACAGAATTGTAATTCAAGATATAACATTCCAAGATTGTGGAAACAAAACCTAATTTAGGAACATTAGTCATGCAGAAATGGCTCAATTGCTTGACTTCTCTTCTCAATTCTCATCAATTATTCAACAAGGGCCCACTACACAAATGCTTAACCCCCAAAAACTTTGCAATAAATACTTAAACCTGTTGTAACTGCCCTCCAAAATATCAGATTATTCAAATCATCAAGCCATAAAAAATATGCCAAGTATGTATGAATTATACCTTAGAGTTAGTCTTCAATTTGTCAAGTCTAAATCCTTGGTCCACAGTGTTATATTAATGCTACTGATACTAACTCGAAAGGGCTAATTAAAGTAGTCATAATCTTCAAGTAATACCACAGTGAGATATATAATAGAACGTCTAGAAACTCGATGCTGGATTGAGTGGACCACTTACAACATGTGAAGGGAAAGGTGACAAGGATAATTCTGTTTTCTCATGCGCCACTGTAAGACTGATATCTGCAGAGAGGGAATTACCTTATAATCTTCCACCAGCCGCGTTTTTCCATTGCATCAACAACAAAATCTTCACAGCACTTGACAGACTTTCCACAAATGATAGTATCTCAATTCAGCCCAACCAAGTTCAAAGGAGCTGATTTGTTAAGCCTCTTTAATGACCTGAGCAATGCCAAATGCTGATCTGCACCTTTGAAAGGAATTTCAAAGAATCTTTCTGCATTTTTGTAGCGCAAAACACACCTGCAGGTTACAAACTTACGCTCAACTTATAACATTAAATGCAGGTATTCTCAACTTCTAGCTACTAAACAGCTTATCACGCCAAAAATATCAAACTCATATAATTAAAAGTAGTACAGGAGCAACTATCAGAATAGAAGAAGGCAACCAAAACAAAGAATAAAGGTGACAACAGTGACAAGATAATATACAAATAGGATGTTATGAGAAAAGTTACCAGCATAGAACCAAAAAAAGCTGATCGTCCAAGTTTAACAGAAGCCATATAGTTTTTAGGGCTTTGGAGGCCATCTGAGTAGAAGCAGTAAAGAACACCCTCAGTCCAAAGTAAACTACATTTGTTCCTTTATTCAGTTCGTCAATAACCTTAATACCCAAAATAATCATCCATAACTTTTTCCAAAGATTATATAAACATTCTGTTCCTTTTTGAGTTTTACCAAAAAATCAAGCCTTCAAACCAGTGAGAAGTGAGGTTTTTCTTTTCTTTTTTTTTTAATTTACCTTTTCAGGTAGGCAGTTTTCAAATACACTGGTCCATTATTCAACAATTTGTCATTGTACATATTAAATTTAAAGACCCAAAAGCCACCAAGTCTGCAAATTAAATAAGCCACCGAACAAATTGAAATACTGGAATTAAGCTTACAAGGCTCAACAAACCTATGATTAGTCAGTTGTACTTAATGACACAATGGGACCTTTTGTTGTATATCACCAAGTAACAATGCTAACAAAGGCATGCAAGTATAGTGAAATGAGTCAATGACCAAGTAGCAAAAAGTTTATTAAGACACGTTTACTTCAAAATGTAGGGCTATCTACTTATTTTATATAATTCAATAAGCGTTATCTGTCTGATTTATAAGCACCATATTGCTGCTTTTGTTTAGCATCTTGAGAGACCACGGATGCTTTCATCAATCGAATCAAGTAATGATGTCTTCCAGTAACCTTATTTTTCAGAACCAGGAACATCTGATTATACTCAGCCTATATTACTAACAGATGATGTGACAAAGGAAACGTGAGACTATTTTAGGGCTTTCCATTTCATAAACACCTGGGTCACAACTCACAACAATGTGCATGCACAACAAACAATTCATTACAGTTAAGCAGAGCATAGTTTTATTGATTTAGCCAAGTCTAGCACTGGACACATATCCTGATGTACTCTGAGTCTCTGACAGGATTGAGAAACTCACGAGTCCAATAATAATGAGACAAATGCCAGTTACTGAGCATTAAAGACAGAGCAGCATGTAGCAATAGATTTAGTCAGCAAACAACTTCTCTGGAAGGGCAAATATTTTAAACATACCACTTGCTCCAGGTGTTGTACTCTCAAAACTTAAGGCTTTATGCTAGCAAATTATTAATCTTTGAAACAAGGTTTACCACAATTTATTTGTACCCCATCATATTGCAACCTGATATTGCATATAATGTAAGTAATTATCACCTAGCAATGAGTTCAATTTAGTTTGTTCTTTTCTTTCGCCCATCAATTTAGTTTGAATCTATCAAAAATTGACAATGACCACCATTGTATTATGTCTATCTACCCCATTGCTAATAGTAAGAGACCCCACTCTCTCATCCAATGAAAGTACCCCACTATATCCCATCATATCCACCTTTTTCTTATCATAATGTGTTTGATGTCAAACACCAATTTATCTCCTAAGCTATGCTTCATGATAAGTAAACAAACTAATTATTACTGAGGAAATATTTGTATACAAAAGTTAAGTAGACAAAGGAGAATACTGGCTTATACACTAAAGCTAAAGATATCCAGCCAAGAGATCATGCAAGTTCTCTAAAGAATTGTTGAATAAGGACATTACTGGACCCAATTCCTCATATACAGGTGGCTGGGAGCATGGATGAGTGGGTTGCCAAGTAGATAAGAAGACAGACAATGCCAAATATGGATAGGATAACTGGATAAGGCACCAGTATTTGGAGGGTTTATCTGCCTTTCACAATGATTATATTAGGAAGTGATTGGGCTACAGAGGTAGTGATTAACTAATCTTCCTGCATACTAAGATTGATGAATCATGACCCTTGATTCCAAGGACTTATCTACTAAGGTTGGAAATTACCATATAATAATATTATTAAGGTTACCATTTTATCATCCATTAAGGTTAGGAATAATTATTCCAGTCACCCCTTGTTCACTTTAACAAAGATTCCCAAGATATTTCATGCAATAATCTATGATTACATAGAAATACATAAGTAGGGAACTTTACACATACCTCTTTCTCTAGCTCTGAGGCTGGAACACTCTCATTAACAAATTATTTTGAAAACCAGTTCTGCTTTTCCTGTAGTAAAACAATACCTTAACAATATAAGGCGCCAAATAATAGGATTGAAGATGCAAAACTAAATATAAAATCAAGAATATCATTTTCTTACCTGCTTTTTATTCCTCTCCCACAAGTTGCTTCCCAAATTTCCGTAATGCAAGTGCATAAGCTTCCTTCCTTTTCCTTGAAGTTCCTTCAGCAACCAACTTGACAGCCTGCATCAATCACATATCAATGGCTAATACACAAAAAAAAGTTTGAACATTTGAATCAATTGAACAAAGGCATACAGCTGTAATACAAAATGCAGCAACTTTATATACAATAAAAATGAAGAATCATCCAACAAGCAGGGGTAATGTTGATGCAAGATATATACTTGACAAAATAACGGCAGGTACAAATAATACAGGGTGGAAATCGTGATATATGCTTCTGCTTTACCAGATTTAAAAGTGATTGATTTTCTTCATCAAAGCAGCTAATGTTATTCAAACACTCAACAAGCATTTAAAGACCACAGCTGGGTACACACAACGCATTTTAAGAGGTAAATATACTTAGTTTCAGATAGAAATGTAAGCTGATATGATAAAGCACAGATTACGTGTGTGCTGAAATTATGCTTCCTGAAAAACAACATCCAAGCACATTCAATATCAAACAAGTTTCAAAAGCAGAAGTCCATAGGAAGTCATTCCACCCCACCCCACCCCACCTCCCACCACCACAACACACACATAAGTTCAAGATTGTGATGTTAAAACTGAACAAAGTTAAATTTTTAATTACAAGCACAAATAAAAATTTTGAATTTTCAAATTGTTTAATTTTCAATTGATTTTTCCACCTATGCATATGAGGAAATTCAATTGATCATATAGCGATTGAAACATTCAAGAGAAATTGTAAAGATTAGTAGAGGACAAAGCCAACAAGAAAACTACAACAATTAAATAAGCATTTCAGAGGAACTACATCAATAGACGCATATTTCTCTCCAAACAATTAACTACATAGGCAGAACAATTTGCCTCTGTCTTTCCATGATCCATGCTCTCTTTTATTTTACAAAGATCATGCTCCCTAGATCAAAGGACTTTCTAGACATATAATCCATGCTTCAGTTAAAAAGTAAAACTTATTAATCACGCTGAAAAGTACACCCACAGATACAGAAGGAATGAACATCCATACCATCGATAGACTATGCCCATTAGTAATGAGGCTCCTAAGTTCATCCTCAATCTGAGAATCTGAGGTGGGCTTCATCAACACAGTCGGCCTTTCCCTCTATTAGAAGTGTCATTTCTCCTTTGGTTTCCGAGATACAAAATGCCTCATAGCGTCACTGCAGTCCTGCGCCAAAACTGCAAGGCTCATGTTAGTTAGAACTCAATGACAAAAAGGCCAATATCAATCTAGTCTAGATGGTAATATAAAATATATAATAGTCAAAACAATCAAACAAATGCTAATATAATCAGATCCATAAATTATGGAAATACTTTCCACTTATTCAAGTAGCTAACAAATCACTTTATCTAAAATCTTCATACATCAAAGCAGCAAACCCTCACTAGAATCATACCTCTTCATGGATTTTTGGTCATTTCTCGAGGCTATGACACATCTCACCAACATTAAAAGTATAATATCACAATGATAAATTACATAAACAAAAAAATGTTTAGGCAGTTGCAGTCTTGCAATTATTTACTCCTCCTTTCTCAACTTTCTGCATCTTTCCCTTCTATTCCATTTCATAATCCCTCCACCCAAAACTGATTGCTTCAATGGAGACAAAATGCCAAGAGGAAACAATTTGCCATAACCCAAATCAAAGTTACTTTGATTACTAAACAAAAGCTATCAGCTAAATAATCAAACAGGAGTCCATGCTTTAAATGCAATTAAGTATAGAGGTGCTTGTCCTTATTTGTAAGTACCATCCGAGAAGGAAGATAAGGACAAATCACTGAGGTGAAAGGACAGTAATCTAGGAAAACGACACGTCCTTTACGTACACAAGATAATTCTCTAACAAAATGTATTGAGAAACAATTTTTGACGCAATTTACAGCATGACTACACAGAGTAAGCACAAGCATTTGCCTATTTTATTAGATATCATCCGATTTGCATCGAGTAAACATTTACCTTGAGTCGCCAAAAACTGGAAAGTTTCTTCAAGAAACTGGTGTAGCTTGTCAGGGGAACTTAAGAAAATCTGTGTCGCACACTCTGGTTGGCGGAGAGCAGAGCCTCTCTCTTCTAGTTGGCTGTGCTTAGGAAAGAATCCAACTAGATCAACAAAGGAATGGAGGAATAAGATGCCATACGACTACATTAGGAAAAATTACAGATTTGTTTGATGGTTTGCAAATTACCGAGGTGAAGTCATCAGTTGGCAAACCGAGCCAGACTAGAGCAGAAACTACAGTGACTCAGGGATAGAACTACAATAATTTTTCTTCTTTGCACAGGCTTCACTGTGGACAAAAATAGTCAATACAGCTTCATAAGTGAGAATATTTTCCTCATGAAGTATTACATCACAACTCAATTTATGGTAACTGACCAAATCTTGAGTTAAAAGCAGAATCTTGATTTTTAAGCTGGTAGCAGGCAACTTTGTGTAACAGAGAAAAAGATATGATACAAGAACTATAATTGGTTAGATGAAATTCTGCTTCATGATGATAATATCAACGAACAGAAGGCAAATACTTTTAAAGGACATTCCACTAAAGTATAAGACACTTACATAAAGTGGATTGCAAATTTGAAATGTAAATCAAGGATGAAATTCATCACAATCCACCCCTTCAATGAACAGATTAAATGGTTAGATAGCAATATAGCATTCTAGCCAGCATTAGAAAAAGTTGGGATAAATATAAAAATGAGATTAAAGGGGCGCATCAACTGATAATTCTGTTTCCAGGTCACTAATACCAGGCATCCCAGCATCACTAATCAGGCGACAATTTCACCCCCTCTTCATAAGCCATTTCAAAACAGTTTGGCCTCTCACGAAACTAATTCCTTTTGTTGGCTGTCTAAGAAGCAAAAACAGGCTTCAATCAAGATTACAGCTAGCTGAATTAGGAAACAAAAACAATAATCATCATTATATACATACAAGGAAGCGTTTTGATTCCATAATACTGGAAGCAACTTTCAGAATGTCTAGTGTCCTAGAAAGTATCACGTCAGCTGATTTCAGCACCATAGCACTAATTTAAGATCGAATTGAAAGGGAAAAAAGTTCGATACACCAAAAATAATCTCGTCAAGAAACCCACACAAGCTCATGAAATATCAACAAGTAACAATTTTGCTGTAGTCCTAGTGTCCAATCAATACCATAACACAAACCAACAGCTTGAATATCATCAACGCACCTCAACGGAACAACACATAAACACTAAAATCTTCAACATTCCACATGATTCACCTTTAAAGGTCAAAGCTAAGATTAATGCATGTCGAAAACCTTTAACATGACCAAAAGATGAGGACATTTAACAACAAAATCCTTGCAGCACCTCAGAAAACAAGTTGTTGCTAAAACTCAACACATCACAATTACTAAATTTGAATGAAGTACATATTTCATAGAAATATATTGATTGATATACATTGCATTGAAAATTAGCATCAAACAATACAAGAAATGAAAAAACAAATCGAAATTTTCGGAATGGGAGACAAATACCGTAATGTGATATCTTCAAGATTACCAATGGGAATTGACTAAATACAACCAGAAGCAAGTCCCACTGTCATCGATTGCTATTACAATCAAAATTTTGAAGGACTAATAATTCCTAATTTGTAGCAACATTTTATATTAGAGTATGATTTAGGAATCAAATAACATACATACCTGATTAGTATTATAATAATTAAAATTAAAAGGGTACAAAATTAATCAACAAAATTAGGGTGTTAAGGGATAAACCCTAGTAAATTTTCCAAAATTAAAACTTGACCCAAATAAAATTGGCAAAATTAGAAGTTGACCCATTTCCCGGAAGTGGTCAAGGAGAAGAAGAGAGAACTTTGTTTAGGAGGTTGTGGGAGGGTGAAATCGGGTGTATGATCGAGAGGCGACATGAGAGTTGGACGACGAAGCAGCCGAGGCGTATTTGCGGGGTAACGGAAGGAGGAGATTGAAGAGGAGAGAGTGGGAGAAGTGAAAGGCGCCACCCAGTGAAACTATCGCCGAATTATCGAAATCGCGATCCATAGTAGTAAGCGCGCCACCGCCATCATTTCGGACTACGCGGGATAATCACACTTTGCTGGAGTTACTACGACTATTGGGGGTATTCTTAATTTTTATCAGATTCCCGAAAACGTCTCGCGGGTCGGCTAGGTCAATCTAACCCCAAAAATGGATTGGGTTAGAACCGAGCTGTAGGGGGTGAGAATCGGTCAAACTCTCGGAATCGGGAGGAAGCTCACAACCGCGGGTATCCACCAGCTCTCGATAGATATCTAATCTAAAGTAATCTCAACTTTTTAACTTCATATTAGTGTGGGATTGAAATATGTAAAAAACTACCATCTTACATTTGATAAAAAGTTTTTAGGGATTTGGATTAGGTAGGTTTAAATAATTTGATCATTTATATTTATGGATTTTTTGTTGCTAATACATGTTTGACTAATAACATGCATTTGTTGATAAGTTTTTATTTCTTGAATTATTTACCATTTTTTTTTTTTTTTTTTTTATTTCTTCAAATTTCACGAATATCCACCTTACTTACTTTTATCCATTCAACGCCTAGATTGTAAATAAGTTTGACAAACCATCAAATTGCTTGACTAATTGGTAGGGAGCGAACCACTAAAAACATGTTACATATATGAGATATAATCGACATGATAAGAGTATCATTTGTCAGAGTGGTAACCACATCATAATAAGTTTTAGCAATCAAAAACTCAAAATTTTTTTGTCTTATCAGTGCCCAATTTTTACATTATTTACATGTAGTAAAGCACATGTCAGGTGTGACAAATAGAGACATTTATCGATGCGAGGCAACTCGTTCGATTTGATGCGGCTCGTTGAGATAGCAAGGGAGTAAAGTGTTACAGCTCAACGCTTGAGGTCGGGTTAGGAAAGGCAAACAAGGCTATGGTAATATCGTTCGATTAATCGTTTGGTGAAGGGTATAACTACCGTTCGTTTGGTGAAGGGTATAACTCTGCCCAGGCGGGAAGTTGGACTACGTACTTTATGGCCGTCTCACATGTGCACTTCCTTTGAATAAGTGTCTTGAGTCGCATATAAGGAGGAAGGCAACTTTGTGCGCCGCCCACAGCAAGGCTTATTCCTTTAAGGGTAGAACGAAATGTAGATGAACCACATTACATCCTCATGAAATATCTACTTGTACTTACAGCCAGGATAATTTGATGAAATAATTATTAGTACTTCTTTTAAAAAAACTCATTGGGTGATGGGAGACTGGATGTTTATTAGTGCTGGACCAAATAATTGAAGCATAGAATTGCACTTCATCACTTCATTGCTAAGACAAGCAACTATCACAAGGCTGAATGTTGTGTATGAAGCAACTGGGGAATTTACTAGTTGATACCAGTCTAGCTTGATCAAATTAGGCTAAATTATTACTTGCTACTAATGGGAAAATTAAAAATCTTGATTATATATAATCAAGGGCATGCCAGAAAAACACCCTTACTTTATCAACAAATTTTCTGAAACATCATAAAATCTTCATATGGATCAAACTACATATTCATGTCTACGAATACTTTACCAGATGGACTATTAAATTATAAACAGCATTTTATTTGGAAATTAAAAATAAAATAAGTAAATAAATTAAATCATTGTAAGTGTTGTAAATTGACAATTCCCTCAAATGGATTACCTTGTGCACAAAGATCCTGGCACACTATGCAAACATGAACACACTTTGACTAAGCAGCCGCACACACATGCCAACAGGCCCAACAACTCTATGCTTGTCTAAACAAGTAGAATTAAGATATACAGGTTCACAGGTACAACTGTACAACAAAGTCAGTTTCTATAATAGTAGCATATTATTAGAAGGAATGATGATAAATTCTTACCATATAATGGTTTATGCTCCAAGGAAAAAAATGCTACAGAAGTTACAAACTGGGATGCTGAGGGAGACAGCTTCAAATGCGTCCTTTAGACAGTAAGAAGCTGCTGTCCACACAAGAGATCTAAAACCCTGCATTACATTTTCAAGGACATATTTTAAAACCACGGAGACTTTGTAGGATTTAGAAACTGACAAAAGTCGATCATAACAACAAAGATGATTCGGCAGCATTCCGCAGAAGTCCCAGGCTAGCATTACAAGATCCCCCACGCGATCACACAATTAAAAAGAGAGAAGGGGGAAGAACAAAATCACATAAACAGACAAACAGGAAGAGGAATTTGTATTCCCTCTATTTTCCAATAATTAAAAAGAACACTCCTGTCAGAACATAATTCCTGCCCACTTCTTTCTTGTGTCTAGATGGAAGACACACAGAGGTAAAGGTTGATAACTGATAAAACCATTAATTTAGCAAAAAGAAAAAGCAGCTGAAGCTGCACTCTTAAGGAGAAGATGTATACATTATATCACCTTTAAAATAAGTCTACTGCAAAATAAAACATCAGATATCAGCAATTAATTAGCAATTATTTACCATCATCCTGGAAGAAATATGTACCCCTCACAAAAGGTCACACTCAACAGCAGAAAGTTTTAAAAAATATCTGCTCATGCTTTATTCAATAACTGGTATTAGTGACAAAAATTCATATCATGTCTTCTGTACCAACATACAAGAGCGGACTAACTGTCTTAATATTGTTATTAACAACTAACTGCTGGTGTTAGAGCTAGCTACGGCAACAATAAAAGGACAGAGTGAAAAACACTTGAAAAATTAAAGGGATACGTATCTCAACAGACCTATCTCACAAGGACACTAGTAGAAGGGAGCAAACATCCAGTAACTATCTACTCACATACATACAGAGATTCTTTATAATTATCAAAGCTCTGCCAACTATCAAACAGATAGAATTATGAGGCCCTAATCGACATTATAACCATTAAACAAATGGGAAAATATATGAATCATCGCTAACATTAAGTGAGAACCTGCTTAATATGCTTCCGCAAACACAAGAGGCTGTGCTTTATTAATTATTTTGCATTTCCTAATATCCTCTCTAAACTGGCTTAGGATGACTTTTTCTTCAACATCTTCACCCCTCACATAATGACAACATCTCTCTTGGTTGTAAAAATCCAATACCAATTTACATATACTAAAAAGCTTGCCAATTTGGGTAGCCTATCTAGCTTTATTAGTTTCTAGATTAAAAAAAAAATTTGGACAGCATATACAACAAGCCACATACATATGCACCAAGCCTTATGATGACTCACTAAACATCTAAAACCTTATCAAAATTTTCAAATCTTGAAATCCTTTAGCCCTTCATAGTTGACACATCAAAGCTACCAGTGCCAATGTCCTCCAGATTCACAGCTAATTTCTCACCCTTCCAAAGCATCAATATGTCACCAAGTCAAGCATAAAATGTTGAGTTCTATATATTGGAATCACCTAGGTCTAATCTGAGTTCATTTCCAGCAGAATATTAGAAGCTACCACCCCAGCAACTACTTTTGTGAGAGCATAAGTATCCCATCCTCTACATGCTATGGAATAATATAACATTGTAGCATGCTATATTGAAGTACCAACCACAATCCTTCACATGACTCAAAACGATTTCATACATATTCCTACAAGGCTATAAATATGCATCTCACTAGTCTTGATCCCATCTAAGCAACCAAACATTCAGTAAAATTTAAAAAAGTACCACCATCCACCACCAACATCCACCATGGTAATTTTTAAAACACCGCGGTTCCATGGGAAGTTGTGAATTGAAGCTGGAAATCTCTCTACCTCTGAACTATACTTTCTAAATCATTATAAATATATTCTTCTACCCCATCTCAAAAAGATTGGCCGATTTAACTGTGATTTGCTTAACTCTAACCAATTCAAATACATAGCTATAGTAGATAAGCCATCTACTATGGTCTTAATATAAGTATGTCACTTCTAACTCCAACTACATACTACTTCAAGCCAAAATCTGACATTAAATCACAACGACTTTTCCTATAAATTGGCAACTTTAAGAAGAATTAAGTAGCAATGACAATGAACATTATCAATCCTAAGTCTAAACCACAAACCCAGGACCATATAATCATAAATTTACCCAGATATATCTAAATTTAACAGCAAAATAAAAAAAATCACACCCCATTTCATAAAACTAAAATGTCATCATACATTTCACCAACAGTTAAAAATAACAATCAATTTTCCCAAAAAAAAATCAACCCACAATAAAATGATGAAAATCATAGAAAGTATTAACCTGGGTAAAGTAGATCAAACAAATGAGCCATATACAAAAGAAGCCCAAAGGCGGATTTGAAAAAGGCTGCTTCGACCATTTATCATCGTCAGATTTTATTTTGCGCCAAATGATCCAGTGTCTAGACCTTTGATTAATAACTATAAACCCCAGAAATTAAAATTAAACAATACGATGTAATTGCACAGGATTTTTGTAAAATCTTTTCATAAACTCGTCAAATTCATCAGCATTTAAAGAAGTTTTTCTGGGTGCGAACTGTTTTCCGGCGATAACAGGCAGTTAGTTTTATTTTTATAAAAAGGGAAAAGAGTTGTAAGAACATTTACGTGGCGGATAATCATTGGGTGTTGTTAGTTGTTCTCTCCCTTTTATTGGTTTGTTGCATGAAGTATTTGTTACGGAATACGGATACGGTCAACTTCGTGAAAACTTGAGAATAACCACAGTGATCATGGATTCATGGATGTTTACTATTTTCCTTGCTTGCTCCACTAAAAAAAAATTGTTTACAGTTACTCTCAGTGTTTTTTAAGTGTTACGTTTAGATGTAGCGTAGTTTTGAAGCTGATTTAATTAAGCCAAATAAAGGAAGAGATAAGTTGGATAATTCTTTAGTTAATTAAGATAGTGGGGTTAATAGTTTATTGAAAAGTAATGAATGTGAGAGGAAAAATATATTTAAAAAATGTCCAGTAGGTTTGGATGGACTATGAAAGAGAAGAAGTGTATACTTTAATGTAGGCACAGCGTAATCTTTATTAAGCAACGGCCCAGGATTTGATAAGTGCAGCAACCCACTTAGGAGGGAATATTTACTCGTTGTCATAGACAGTATAAGTTGACTTTGAGTGTTGATCTCTAAAAGTCAACTTTGGGCCTTAGAATTAAAAATATTACAGGTCAAAGTCTTCTTCAATTATTGCCCTAACCAAGGTATATTTGAGAATATGTAGGGAGAGTCACCTTTGCATAAGGTTAATACATATACTTTGTATACAATGGTAGCATGATCTATTTGGCTTTGTGTTTGCTTTGGCCAAAAATTAAATAGGTCATAACCAAGCAACATTTCCTTTGTGACCTATTAGGTGGACGGAAAATTAAATGGAAGTTAACTTAGGTGTAGATGTAATCGGGGCAATAATCTACATGTGGTGAAATCTTGCCTAAAACACTTTGACTACTTTTACACTATTTAGCATGATCAGAAAATCTTTTAGACTCCTCAAGTTATACCTTAATAACATAAATCTTTAGAATTTTTAATTTATTGCTTATTATTTATAAGTGAGATTAAATGTCTTTCGCATGCGTGTGAAATAAGAAAAGAACACTTAAATAAAAATTAATAGATTTAAGCATAGGTAAATGTTTGAAGTTTACGATATAAAGGGATTAATTAGGGCTTAGAAACAACAGGGTTTAAGGTTGGATAAAAGATATAGGTTTATGGTTGGGATTAGAGTCCACATAAGAAGGGTTAAGGCTAGGGTTTGGTTTGAGTTTAGGGTTAAGGGTTTAGGGTTTAGGGTTAAAGAATTGCATGAGTAAAGTGTAGGGTTTGAGTCGAGGTTTGGGATTGGGGTTTAGGGTTTAGGGTTAAGGGTTTAGGGTTAAGGGTTTGGGGTTTTTAGGGTTAAGGGTTAAAGGGTTTAGGGTTTAGGGTTTAGGGTTAAGGGTTAAGGGTTTTGGGTTTAGGGTTGGGGTTTAGGTTTTTAGTTTAGGGTTTAGGGTTTAGGGTTAGGGTTTAGGGTTTCGGGTTTAGGGTTAAGGGTTTAGGGTTAAGGGTTTAGGAGGGTTTAGGGGTTAGGGTTAAGGGTTTAGGGGTTTAGGGTTAAGGGTTTAGGGTTTAGGGTTTGGGGTTAAGGGTTTGGGTTTAGGGTTAGGGTTAAGGGTTAAGGGTTTAGGGTTTAGGGTTAAAGGTTTAGGGTTAAGGGTTAAGGGTTTAGGGTTAAGGGTTTAAGGGTTGCAGAGGTTTAGGGTTTAGGGTTTAGGGTTTAGAGAGTTGGGGTTTGGGTTTAGGGTTTAGGGTTTTAGGGTTTAGGGTTAGGGTTTAGGTTTAGGGTTTAGGGTTAAAGGGTTAAAGTTTAGGGTTTAGGGTTAAGGGTTTAGGGTTTAGGGTTAGTGTTTAAGGTTAAGGGCTTAGGGGTTTAGGGTTAAGGTTTAGGGTTAAGGGTTAAGGATAAGGGTTTAGGGTTAAGGGTTAAGGGTTTAGGGTTTAGGGTTAAGGGGTTTTTAGGGGTTAGGGTTAAGGGATAAGAGGTTTAGGGTTTAGGGTTTAGGGTTAAGGGTTAAGGGTTTAGGGTTTAGGGTTAAGGGTTTTAGGGTTTGGGGTTTAGGAGTTTAGGGTTTAGGGTTAGGGTTTAGGGTTTAGGTTAAGGGTTTAGGGTTTAGGGTTTAGGGTTTAGGGTTTAGGGTTAAGGGTTTAGGGTTTAGGGTTTAGGGTTTAGGGTTTGGGGTTTAGGGTTTAGGGTTTAGGGTTTAGGGTTTAGGGTTTAGGGTTTGGGGTTGGGGTTTAGGGTTTAGGGTTAAGGTTTAGGGTTAAGGGCTTAGGGTTTGGTTTTAAGGGTTAAGGTTTAGGGTTAGGGTTAGAGTTTAGGGTTTAGGGTTAAGGGTTTAGGGTTAAGGGTTAAGGGATAGGGGTTTAGGGTTAAGGGTTTAGGGTTTAGGGTTAAGGGTTAAGGAGGGTTTAGGGTTAAAGGGGTTTGAATTACTGTTTTGTCCTTATTTCATTATATTTTTATGAAATTATAGAAAAAAGTCACATCCAGAATTGACTTATGTTTTTAAAAAAATAACTATTAGAAGCTGATTTCTCTACGGAAAAGTTTCATCACTTGGGTTTCGAAATCGGCGATATTGATCCTTTTCAGGAATTGAAAATTTTGAGTATGCCAATTTCAAGTTGAAATAATGACTTCAAAAGAATCGGTGTTTAAATAAAAAAAAAAAAAAAAATCATCGAAGCCTTTACTAGAAGATTTAGTTTCACACAAATTATCAAACTGAAATTTGGTTCCAAAAATACAGGAGGTACTTAAAAATGTGGTGGAACAGCCGCTGCTTTATTGTTGATCGATATTAAATTTTAATCCTTGAGAGTAATATTAGTAAATAATAGAAAAATTAAAAAAATTGAAACGAAAGCAGAATATTTATTTCTAAAATCGAAGATGACATTGATCGGTTGTGATTTTGTGAAGAAAGAGGACAAGAACCATCAGCTGGCAGAAGATTTCCTTTGGCGATTTTGTGATTATGTGAAGAGAGGACAAATGAAAAGGAATTTTTATTTCACAGGAATGAGATATGTCATTGTTACCGGGTTTGTGAAGAAAGGAAAAAAGGGTTTCAGTGGGAAAAAAGCAGAGCAGAAGACAGAGGGAATTAGAGGGAGAAAAATAACGGAAAATATATAAAAGCTGAAAATTATATTGTGGGCTTTGTAGAAGCGTTGTAGTTTGCGGAATCATTTATATTTAATCCTCTTTCTGAGAGTAATATTAATGCGGAGCTGTCGGGATTGGCATTTGGTTAGCTACACCACCTGTAGTGTTTTCAGCGTAATTACTAAATTAGGCTATGTTTGTTATAAATATCTTAATTTACTATCACGTTATTTATTTATTTTTGATCGGAAGAAATACGTCTTATCCCGTTAATTCGTTGCTCTGTTTATATAATCAGTGATTTATATAGGACCTTGTGTATATGAAAATAGGCAAATCCCTAAATTATATTTCTATATAGGTAACAAGCATATCTGATAATTTGGAGAGTGTACCAAACTTCAACTAATCTTAATTCGATTTGAACTTTGGTCAAGCCTCGATTGGGGAGAATGATCTCCCAACACATCTAATTGTTTGTGTTTTTAAATCTTAGACCCACGTGTAGATACGAGAAAGTCCTAATACATCACCGTTAGATACGTACAGAATTTGAATAGGCGCTTTCTTTCTTGAAGTCTAATATCTTATTAGGGAGATTTTTTTTTTTTAAAAAAACTACTAATCATATCTAGTATTACCAAGATATTAATGAGCACTATATCACCTTTCCGTAGTACTCTCAAGAATTTTTTGTGACCATGGTTTATTTACAAAATTGTCATTATTATGTACTCGTATTCACTTGTGTCTAGCAGTAAGGAGAAGCCCCAGGGAAGCCCTGGACAGATACCTTGGTTAAGATGGAACAGCTCCTTTCCAACTGATTCAAATTAGGGTTTAATTACAGCTGTCAATCTGGATGTAAACTAGAAGCTCAAATATTGATTACCCAAGTATGAAACTCAAAATGATATTTCTTGATCCCAGCCTTATACTTGTATGAGAGTTATATGTTAGTTCTTTTTTGTGTTTGATTGATCCTTGTATGGCAAACTTCAACCTCTTTTAGCATCAGCTGTAGCAACAATTCATTATGTATATAAATATTCACACAGAAAGCAGAATAATAGGGTTGAGCAGCAACTCAGCTATTTATCTTCACTGTGTGTGTTGCATACAGAGTATTACACACCATTATGTACCATGTTAACTTAGCAGTACATTGGGTATTAGCAAACTGGTAAACAAACGGCTTAGAGTGTAGGAGTAACCAGAGGCGCTTATAAGTGAACTTGACTCAGAGCTGCCATCCCCAACTTGATTACACCGCTCATGAAAACTGCTGATTGGCCGTGCTAGGCATGCATCCCAGCGTTCACTGACATTGTTTCTGCAACTGTATATACTCCTTTGGAATCAGTGAACGCCTGATAGTTCACAACCTCCCCAGATTTAGTTATACACAGGACAGCCACCTCGGCCTCGCGAACACAATTATGCAGTATAACAAAGTTAGCTACTTCAGCTGCAACAATAGCTAAGAACTTCTGTGCTGTATTTGCCTCCTATATTTGATCAATACATATTTTCTTTTTCATTCAAGCATTGCTAAGCCAAGAAGTTAATTACACGTTAAAACCAAGTTTTGGTTTTCAGCTTCAACTAAATTTGTCCTACTCTCTTATATTAGGAACTTGAATAAGTCATGCTCAATGGCAAATTGAATGGAACCTCCTACAATAGCACATGTACCAACATCAATAGACTAGCACCATTTGGCAGCAGCAGCAAGTGAATGGAACCTCCTTACTAATTTGCTTCATTAAAGGCAGGATGTAATTAAGCTGATTGTTATAATGAATCCTATTTCCTAAGAATAATGTAAGGACATAGTTAGTACTTGGTAGAAAAGTAATGTAAAGGGATGATGGGTTGGTTGTTAGATTAACCAACATAGGATAAGAATCCCAAAGACAGTAATTTCAGAGGCCTAAAACAGCATGAACTCTAGTGGTTTCAGCATCACTTAAAAATACTACTATCTGTACAAGTTTTGATGAGTTTATAATTTGAGATCGCTATACAGTTTCTACGCTCCTAAGAAGAACATTCATGAAAACCCTAACGGAACTGCCCTCTACTCAGCCAGGGATGCTGCAATCACAAGGATTGTTACATTCCCACAAAGCTCTTTTCCAGAGTTCGGGTTGTTTGACTTAGTAAACAATGACAAATAGAAGCCTTCTATCATATGTGAGTGTCCAATCAACTTCTCAAAGTTCAACTTCATACATGAGATGTTATAGGCCCAGTATCCACACCTCCAATGAGTGAATTTGAGGGTGTGAAGAGGAGAAAGAGGGACAAGAACCAAGCAAAGCAGGGGGCACGTTAACAACGTGACCAAAAGTCATTAAGGAAACACTAAAAGGGGTGGCATCCTTGGTCTGGTTATTGACGGATTTGATGGTGAAGAAGAGCAGTAGAAGACAAAACTAATTAAAAAAAGTAGGTTACCTTTAGTGATGAATCTTGTGTATATAAAAAGAAGTCATGAGTGGAATGGCAAAGGATTATGTGGAAATTGGCAGAGAACTTTTGAAAGAGAATTCTAGCCTAAAGCAGCTACTAGTTGAGACTATTGTGAGCTGTAAGCTTTCAACCATTATCAAGCAATACATCAAGCTATCACCTTCTTTAATCCTTACTGTTTGCATAATATTTACAATCAGAAAAATAGTCTATTAATGGGATGAAATCTTTTTCATGTCAATATCAAGTCCAATGGAAATCATATAGAAAGGCAATGGTCTTTTGGTAAAAGTGTACAGACTAGGTTGAATTTCCTTGAACAACTAGGTAGGGCATCAACTACAAGAACAATGACAGTATGGATTTTGTCAAAGTTCAGATTATGCAACCTTACCTAAACAATAACAAAGTCCCTCATATAGAGCATTACAAACAAATAATTGAAGTGCACTTGAAATGGAATTTAGAATCCATCCCATAATAAAATTGGAGTAAACAAGTCTGCAGTACTTGAAGAGATTCATGAATACATCCTACATGTACCCAGCTCAATTCATGCAAGATCAGCTAAATAAACTCGGTCAAAAGGCTCCTCTCTCTCCTCACTTTCTCTCTCAAACCTGCGCTCAGGTGTGAATTTTGAGAACTGTCGATTGTCGATTGGAAAGCGCATCATGTTCTTTATTGTTTTTAGGTTTATAATAATCTTCACTCGCTTTTTGGGGAGGAGCTTACTCGAAAGAAAAAGAGTTGTCTCACCCGAGAAGACTGAGGTTTTCGAAATATAAGCCGAAAGAGAATGAGTTTATATACCACTTTTCGAGGAAATTTAACTTTGAATCGTAGTAGCAGATCTAATGGATTGTCGTGAAATTATTTAAATTTAGTGAGAAAAAGTTATCAGTTTTGAATACGGACGAGATGAATCGAAGGAAAACATCGCCATGTTACAAAGAAGGATCGTTAGATTGTCTCTCCAATAATTTTACGTAATTTCCCGGTGGACATAAGCGGGAGATCTTTCCGTGAGTGCGAATCTAACTCGGCGATCGTAGTTTTGAAGGAAAATTACAATTTGATTAACTATGTATTTGTGCGTCGAATCTGTGTAGATGTCATTATGTTTTTAATTAATGAGAATATTTTGCTATCAAAAAAAAAAAAAAATTAGGTGATTTGTAGTGTCAAACTGTCAATGTTCTGATTCAGTTAATTATTTGTGCTAAATGTGTCAAAAGATTGTTAAAACCATCAATATTGACACAATAACGCAAAGGTTGACATGTACACTTCCAAATCATCTTTACTTTGTTATTTTAAGCTCTAACTTGGTATATTTCAATGGCCTGCACTCTAATCTGCACTCTAATCTGCACTAAACACTTTCTGGGGTTAGCGCTAACTTAGCTTGCTCAGATTCCTACTGTGGCATAGCAACATTAAACAAACCTGCTTTCTCATACTTGTAAATCAAAGCCATTGCTTTACATCTGATGTTTCCGTATGTAAGAAGTATATCATCCCGAGCCAAACAGAGGTTTACACATAAATCCTTTTACTCCCGCATCCATTTCCAATATCTCCTGGAGATAGGAGGCAAAATTCATCCTAGAGTCAATTCTATATAACAGTGTTTCAATAGGTCTGACAAAACACTACTCGTATATTACACTTTATCATCATGGAAACCACCACCTCGGCTCCTTAGGAGATCTACCGTTCTTACATTCTTCACATAACGGTCATTGTCTGCAGGGCGTTGCTGCAAAATGTAACAAAATCGATTCAGTTAGAAATAAGAATATGAGTAACAAAAGCTCAAATGGCAACTGCTGGGAGTCGAGGGTGTGGCCAGGGTAAAGTAACTCAACAAACCTTTGAAATTAGAGCTTGAAAGCATTGAAAGAATACTGATACAAATTGAGCTGACTGTCATCGCTGGGACCAAGAGTCGTATAAAATATCTGCAGTCATAGACAATACGAGAAAGTATGTGTTGGCAAGGTCAGTATTAAGACAAATCATCTGTGGTCCATCCAAAGCTGCAAGAACCTATATCAGGAGAGATTAATATCAACAACGATTTGTGGAAGAAACAAGGACTATGAGCTTGCACTACAACATGGCCTACAGCCCTATAATGTTTGAATGACTTGTGAAAAAGATTTCCAATATCAATATCTCATGAAATGCATTACCATCCATTATTAAACAAAATAAAATTAAAGTAAAATACAAAAAAAAAATATATATATCTGCTAGGCACAATAATATCATAATGAGGTGGTAAGCTATTATGATATAAATACAATATTACTATATCTAGGTAAAGATACAATTCTCAAAGTCTATCCACAATAATCACAATCTCCATCACATTAGAGTGGCTGATGTCTTCTTCACATTAGTCTATCCATATATAGAAACTAGGCTGGGAATTTGAAACACGGGCAACACTAAGAGTTTTATAGAAAGACACAATAGTCAATGCATCTATTGAGATACATAAGTGAGATCACACTTTATTTCAGCAACAATAACAGCAGCCATTTAGGAATTTTTTGTCCAGTGGGGTTTGGGAGGTCAAAATCTAAAACGTAGAATGACTCTCAGGTGGAGTGTAGACGGTAGACCAATCAAAGAAACAAGATTATTACTAACTGATGAAAGAAGGGCATTCAATTCTACATAGATCAGCTATATGTTTCGAACCATCTCACAAACTGACCTATCTGGCACCTTTACGTTTTTAAATGAAACTGTAGGTAGTTGAGAAACTGTAGAAATTAATCCTCTATTGTGGCTCGCTTCATTTTATAAATATATATTGAACAAAGACCCATGAAAATACATAAATTAGCTATTATGTGAGGAAATGTAATTTGCCAAAGTCTATCACAAGAATGACAATCTCAAATTCACACATTAGAGGCCATTTTATCTTTTATTAGTTTATAAAGGAATTTACAGAAAATAAGAAGAGGTTAGGAAAAATTGTAACACGATCTTCACTAACTTAACAGAAAGGCACAATAGTCAATGCATCTATCCAAGCTACATGAATAAGATGTCATACATTGTTACATCATTAAAAATGACAGACATTGGGGATCTTGCTTCTAGGGGTTAGGGAGGTCAAGTTAGATAAGCAGTACCTAAGGCGTAGAAAGACTATTTCATATGGAGTTTAGAACCATAATGGAAATATTATATTCATTCTACTAACTGATAAAAGGGTTTTTATCGAGTGCCAAGTGTTCCAAACCATCAAGTAACATATTATGCACATAATATGCCTTGTAAACAATATGGAGCATAAATTTTGTCAACCAAAACAAAGACATTATAATAATTAAGGCTTAGTACTCAATTTGTTGAATAATTATTTATCAGAACAAGGGCACAACTTTTTCAATGGAATGAAGAGGATGATAATTCTAAATTTAAGTCTAGGAGAAAACAGCCCAGCCCTAGGTGCTCCTTAATCTAACATCAGATTGGAACTATGAAAAATATGTACTTATATTAATCTAACTGATTATAAAGGAAAAATCAATAGGCTTATAGGAGGCCATTGTAAAAACTATTAAACCAAATCAACATTAGATTTGTTACTAATTCTTCATAGGCTAGCAATAGCAGTAAAAATAAACAAAAAAAATAGGCACTCAAAGTCAGCTTTCAACAACTTTAGACTCACAAGAACAATGACCATTAGCATTTCTATGCATTGTTATCAGTTCATCCACATTCAAATATCTCCATAATCAGTTATAATAAGCAAATCTGTACAAAGTCCTCTAAAATTTGGAAATTGGAGTTATTCTTTTCATTAATCACTTTCATCCAAACTAATTCTAGGTTCCTCCCTTAAATAAATTAAGCATAAATATCAATAAAATCCAACAAGTGCACAATCAACTCATCACCATTCCTGCCACTATGCATAGGATTACAAATCGTCTAGAATCACCCATTTCCCTCTTCATCTACATATCCATATACTTCAAATTTACGAGGACATGTCAATGCATTAACAAAACTTTCACTAATCATTTAATGTAACCCTAAACAGCTACAACTCAAAATACAAAATCACAATATATAACATATACGTAGTATGAGCAATGTCAAGATTGTCAACTGTCAAATGAAAGTTTCTAACATGTCATTCCATTCAATTGACTTCAATAATACCCCCAAATAGCCTACTATGACTATAATACACTTCAATGATACAAACATACAACATATTGTACTAATTCCACGCCTCTCTCCGTTTTAAACTAGTCGTCACATTGATAGTGTAATTTAAACTAGCTGCACGAACTGAAAGCTAAATTTCTACGGATTTATAATTAAGAAACACAAATAGTGAAATAGAGAACAAATAAGCATTAACAACTAAAAAAAAAAAAAAAAAGTTCATCTGATATATAATTACCTAAACAGATGTACTTACATTGCTGTAAATGTGAGGATGCAGAGGAGCAGGAGGTACAAATATAACCTATACCCCAAATACCAACAAATTAAACCAAATTCTAACCTAGCAAATTATTGCAAATAAAATAAACTATAACAATAATAATTTAACAATTAACATAAAATGATAAGAAATAACAATTTAATTTTCTGGGGTTCAATTGAATTAGAAAATAAATAATAACCTGGGAGTGCTTCCATAGGATAATGCTCTGGAAAATCAACCTGAAGCTGATATGTCTCGTTTGCATACAAAGAAGTTCAGGTGCTCAGAAACTGATCACCCATCTTTTTAACAAAAACAAAAACAAAAACTATATAAAAATCACAACTTTTTTAAAAAATTATGAACAAAAAAAACCAGAAAAATTAATGAAAATATGTGCCACCTTTGAAGATTATCAGGTGACCTTATGTTTGAAGCCAGCAGGAGGATTAAGCTGCCACTCAACAAAAGCTCTTTTTTGGAGTCGATTTTGGGCGATCTTGCTTAAAGTCTGAGAATTTAAACAATCAAACATGTAAATTAGCAATTATTATTATTATATTATACACCCGAGAACACGATGTACAAGTTTAAACTGTACGTGATTTCAAGGAGCATGATACCCATTATGCTATCAGTGTAGATCTAAATTGCGGTAAATTAGGTAAAATTAAGAGATAGAAAAGAAGAACCTTGCGTGATTGAGTGGAGGAACTATTCATGGTTTGAAAGAGAGAGAAAAAGAGAAGAAGAGTGGCAGAAAGTAATTGTGGGATTTTTAATGGGGTCGGAAGATTGAAGAAGATGGAGAGAATTTACTAAAGTTAAGGATAAATCAGTCTTTGGTAAAGAGACATATTCCTATAATAATTCCCAATAAATAAATAAATAAATAAAAATAAAATTTGTGTTCGTTTACGGTGAAATTTGACTTTTCACAATATCTATTTTATATTCTCCGCTATCGTTATTTAAAATGCCTAATTTGATTGCGTATGTAACAAAATTTTAATTGCTTATTTTTTAATCGTATATATCGTCTAAATGTATATAAAGCTAAAAACTTAGTGGGTCGATAAGCGTAACAATAGATACAATTTTTCGCGACGCCAAAGTGTCGCGCCCGTAAAGATAGAGCAATACGT

**Data S4** Example of telomere sequences array in the interstitial position where clusters of TTTAGGG motifs were interspersed with different derivative motifs.

*C. pamiricum*, accession 830-3С, read 36779 (14352 bp). Refer to Figure 1D.

TRF analysis:

Sequence: 830-3С_read_36779

Parameters: 2 7 7 80 10 50 500

Length: 14352

| Indices | Period Size | Copy Number | Consensus Size | Percent Matches | Percent Indels | Score | A | C | G | T | Entropy (0-2) |
| --- | --- | --- | --- | --- | --- | --- | --- | --- | --- | --- | --- |
| 28--291 | 7 | 37.7 | 7 | 89 | 7 | 403 | 12 | 0 | 44 | 42 | 1.43 |
| 287--728 | 7 | 61.9 | 7 | 87 | 11 | 616 | 55 | 0 | 0 | 42 | 1.12 |
| 729--1000 | 7 | 37.7 | 7 | 89 | 7 | 402 | 11 | 0 | 43 | 44 | 1.44 |
| 997--1212 | 7 | 30.7 | 7 | 85 | 11 | 300 | 55 | 0 | 1 | 43 | 1.08 |
| 1208--4907 | 7 | 527.6 | 7 | 89 | 8 | 3689 | 19 | 0 | 36 | 43 | 1.54 |
| 4904--5709 | 7 | 114.9 | 7 | 87 | 11 | 1126 | 56 | 0 | 0 | 42 | 1.04 |
| 5717--6019 | 7 | 43.3 | 7 | 93 | 5 | 524 | 13 | 0 | 43 | 43 | 1.44 |
| 6018--6309 | 7 | 41.4 | 7 | 90 | 9 | 438 | 56 | 0 | 0 | 43 | 1.02 |
| 6306--6734 | 7 | 61.3 | 7 | 92 | 6 | 719 | 13 | 0 | 42 | 44 | 1.45 |
| 8101--8343 | 6 | 38.5 | 6 | 75 | 19 | 187 | 50 | 35 | 0 | 12 | 1.46 |
| 8351--14231 | 7 | 865.3 | 7 | 71 | 17 | 1761 | 7 | 34 | 41 | 16 | 1.77 |
| 8352--14245 | 6 | 897.7 | 6 | 71 | 18 | 1918 | 7 | 34 | 41 | 16 | 1.77 |
| 8352--14245 | 13 | 441.3 | 13 | 71 | 18 | 2269 | 7 | 34 | 41 | 16 | 1.77 |
| 8351--14231 | 20 | 291.8 | 20 | 71 | 18 | 2225 | 7 | 34 | 41 | 16 | 1.77 |
| 11229--11267 | 11 | 3.3 | 12 | 89 | 10 | 53 | 5 | 33 | 43 | 17 | 1.72 |
| 13085--13115 | 16 | 1.9 | 16 | 93 | 0 | 53 | 3 | 41 | 41 | 12 | 1.59 |
| 13285--13328 | 23 | 1.9 | 23 | 100 | 0 | 88 | 0 | 31 | 45 | 22 | 1.53 |
| 13299--13984 | 31 | 22.7 | 30 | 67 | 22 | 126 | 7 | 34 | 41 | 16 | 1.76 |
| 13308--13839 | 51 | 10.5 | 51 | 68 | 17 | 150 | 6 | 34 | 41 | 16 | 1.75 |

Consensus pattern (7 bp):

TTTAGGG

Consensus pattern (7 bp):

TTTAAAA

Consensus pattern (6 bp):

CCTAAA

Consensus pattern (7 bp):

CCCTGGG

Consensus pattern (6 bp):

CCTGGG

Sequence:

>830-3С_read_36779

CGTTACTTCGTTCAGTTACGTATTGCTGTTTAGGGTTTAGGGTTTAGGGTTGGGTTTAGGGGTTTAGGTTTAGGGTTTAGGGTTTAGGGTTTAGGGTTTAGGGTTTAGGGTTTAGGGTTTAGGGTTTAGGGTTTGGGTTTAGGGTTTAGGGTTTAGGGTTTGGGGTTTAGGGTTTGGGGTTTGGGTTTAGGAGTTTGGGGTTTAGGGTTTGAGGGTTTAGGGTTTAGGGTTTAGGGTTTAGGGTTTAGGAGGTTTAGGGTTTAGGGTTTGGGGTTTAGGGTTTAGGATTTAAAATTTAAAATTTAAAATTTCTAAAAATTTAAATTTAAAAATTTAAAATTTAAAAATTTAAAATTTAAAATTTAAAATTTAAAATTTAAAATTTAAAATTTAAAAATTTAAGAATTTAAAAATTTAAATTTAAATTTAAAAATTTAAAATTTAAAATTTAAAATTTAAAATTTAAAAATTTAAAATTTAAAATTTAAAATTTAATTTAAAGTTTAAAATTTAAAATTTAAAATTTAAATTTAAAATTTAAAATTTAAAATTTGTTCAAAATTTAAAATTTAAAATTTAAAATTCCAAAATTTAAAAATTTAAAATTTAAGAATTTAAAATTTAAAAATTTAAAAATTAAAATTTAAAATTTAATTTAAAATTTAAAATTTAAAATTTAAATTTAAATTTAAAAATTTAAAATTTAAAATTTAAAATTTAAAATTTAAGTTTAGGGTTTGGGTTTAGGGTTTGGGGTTTAGGGTTTAGGGTTTTAGGGTTGCAGGGTTTAGGGTTTTGGGTTTAGGGTTTAGGGTTTAGGGTTTAGGGTTTAGGGTTTAGGGTTTAGGGTTTAGGGTTTAGGGTTTTTGGGGTTTGGGGTTTAGGGTTTAGGGGTTTAGGGTTTAGGGGTTTAGGGTTTAGGGTTTAGGGTTTAGGGTTTAGGGTTTAGGGTTTGGGGTTTAGGGTTTTAGGGTTTAGGGTTTTTAGGGTTTAGGGTTTAAAATTTTAAGAATTTAAAATTTAAAATTTAAATTTAAAAATTTAAAATTTAAAAATTTAAAAATTAAATTTAGAATTTAAAATTTAAAATTTAAAATTTAAAAATTTAAATTTAAAAATTTAAAATTTAGATTTAAAATTTAAAATTTAAAATTTAAATTTAAAATTTAAAATTTAAATTTAAAATTTAAAAATTTAAAATTTAAAATTTAAGGTTTAGGGTTGGGTTTGGGTTTAGGGTTTAGGGTTTTTAGGGTTTAGGGTTTAGGGTTTAGGGTTTAGGGTTTAGGGTTTGGGTTTAGGGTTTAGGGTTTAGGGTTTAGGGTTTTAGGTTTAGGGTTTAGGGTTTAGGGTTTAGGGTTTAGGGTTTAGGGTTTGGGTTGGGTTTAGGGTTTAGGGTTTAGGTTTAGGGTTTAGGGTTTAGGGTTTAGGGTTTAGGAGTTTAGGGTTTAGGGTTTAGGGTTTAGGAGTTTAGGGTTTTAGGGGTTTAGGGTTTAGGGTTTAGGGTTTAGGGTTGGGTTTAGGGTTGGGAGTTTAGGGTTTAGGGTTTAGGGTTTAGGGTTTAGGGTTTAGTTAGTTTGGGGTTTAGGGTTTAGGGTTAGGGTTTGGGTTTAGGGTTTAGGGTTTAGGGTTTAGGGTTTAGGGTTTAGGGTTTAGGGTTTAGGGTTAGGGTTTAGGGTTTAGGGTTTAGGGTTTAGGGTTTAGGGTTTAGGGTTTAGGGTTTAGGGTTTAGGGTTTAGGGTTTAGGGTTTAGGGTTTAGGGTTTAAGGTTTAAAGGTTTGGGGTTTAGGGTTTTAGGGTTTGGGTTTAGGGTTTAGGGTTTAGGGTTTGGGTTTAGGGTTTTTAGGGTTTGGGTTTGGGGTTTAGGGTTTAGGGTTTAGGGTTTAGGGTTTAGGGTTTAGGGTTTAGGGTTTAGGTTTAGGGTTTAGGGTTTAGGGTTTAGGTTAGGGTTTAGGGTTTGGGTTTGGGGTTTAGGGTTTAGGGTTGGGGTTTAGGGTTTAGGGTTTAGGGTTTAGGGTTTAGGGTTTAGGGTTTGGGGTTTAGGGTTTAGGGTTTAGGGGTTTAGGGTTTAGGGTTTAGGGTTTAGGGTTTAGGGTTTAGGGTTTAGGGTTTAGGGTTTAGGGTTTAGGGTTTAGGGTTTAGGGTTTTGGGTTAGGGTTTAGGGTTTAGGGTTTAGGGTTTTTAGGGTTTAGGGTTTAGGGTTTAGGGTTTAGGGTTTTAGGGTTTAGGGTTTAGGGTTTAGGGTTTAGGGTTTGGGGTTTAGGGTTTAGGGTTTAGGGTTTAGGTTTAGGGTTTTTGGGGGTTTAGGGTTTAGGGTTTAGGGTTTAGGGTTTAGGGTTTAGGGTTTAGGGTTTAGGGTTTAGGTTTAGGGTTTAGGGTTTAGGGTTTAGGAGTTTAGGGTTTAGAGTTTAGGGTTTAGGGTTTAGTTTAGGGTTTAGGGTTTAGGAGTTTGGGTTTAGGTTTGAGTTTAGGGTTTAGGGTTGGTTTAAGTTTGGGTTTGGGTTGGGGTTTTTAGGGTTTAGGGTTTAGGGTTTAGGGTTTAGAGGTTTTAGGGTTTTGGGGTTTAGGGTTTAGGGTTTAGGGTTTGGGGTTTAGGTTCAGGGTTTAGGGTTTAGGGTTTAGGGTTTAGGGTTTAGGGTTTAGGGTTTAGGGTTTAGGGTTTTAGGGTTTAGGGTTTAGGGTTTAGGGTTTAGGGGTTTGGGTTTGAGGTTGGGTTTAGGGTTTAGGGTTTAGGGTTTGGGTTTAGGGTTTGGGGTTTTTAGGGTTTAGGGTTTGGGGTTTGGGGTTTAGGGTTTAGGGTTTGGGTTTAGGGTTTAGGGTTTAGGGTTTTAGGGTTTAGGGTTTAGGTTTTTAGGGTTTAGGGTTTAGGGTTTAGGGTTTAGGGTTTAGGGTTTAGGGTTTAGGGTTTAGGGTTTAGAATTTAGGGTTTAGGAGTTTAGGGTTTAGGGTTTAGGGTTTAAGGGGTTTAGGGTTTAGGGTTTAGGGTTTTTAGGGTTTGGGGTTTAGGTTTAGGGTTTAGGGTTTAGGGTTTTAGGGTTTAGGGTTTAGGGTTTAGGGTTTAGGGTTTGGGGTTTAGGGTTTAGGGTTTGGGTTTAGGGTTTTTTGGGTTTAGGGTTTAGGGTTTAGGGGTTTAGGGTTTAGGGTTTAGGGTTTAGGGTTTAGGGTTTAGGGTTTAGGGTTTAAAGAATTTAATTACAAAATTTAAAATTTAAATTTAAAATTTAAAATTTAAAATTTAAAATTTAAAATTTAAAATTTAAAAATTTAAAATTTAAATTTAAATTTAAAAATTTAAAAATTTAAAATTTAAATTTAAAATTTAAATTTAAAAATTTAAAATTTAAAATTTAAAATTTAAAATTTAAAAATTTAAAATTTAAAATTTAAAATTTAAAATTTAAAATTTAAATTTAAAAATTTAAAATTTAAAATTTAAAATTTAATTTCGCATTCCTTGCGCAGATTTAGATTTAAAATTTAAAATTTAAAATTTAAAATTTAAAATTTAAAATTTAAAATTTAAAAATTTAAAATTTAAAATTTAAAATTTAAAATTTAAATTTAAAATTTAAAATTTAAAATTTAAAATTTAAAATTTAAAATTTAAAATTTAAAATTTAAAATTTCCAAAATTTAAAATTTAAAAATTTAAAATTTAAATTTAAAATTTAAAATTTAAAAATTTAAAATTTAAATTTAAAATTTAAAATTTGAATTTAAAATTTAAAGTTTAGGGTTTAGGGTTTAGGGTTTAGGGTTTAGGGTTTAGGGTTTGGGTTGGGTTTAGGATTTAGGGTTTAGGGTTTGGGGTTTAGGTTAGGGTTTAGGGTTTAGGGTTTAGGGTTTAGGGTTAGGGTTTAGGGTTTAGGGTTTAGGGTTTAGGTTTAGGGTTTAGGGTTTAGGGTTTAGGGTTTAGGGTTTAGGGTTTTTAGGGTTTAGGGTTTAGGGTTTAGGGTTTAGGGTTTTTAGGGTTTAGGGTTAGGGTTTAGGGTTTAGGGTTTAAGGGTTTAGGGTTTAGGGTTTAGGGGTTTAGGGTTTAGGGTTTAGGGTTTAGGGTTTAGGGTTTAGGGTTTAGGGTTTAGGGTTTAGGGTTTTTGAGGTTTAGGGTTTAGGGTTTAGGGTTTAGGTTTAGGGTTTGGGGTTTAGGGTTTAGGGTTTAGGGTTTAGGGTTTAGGGTTTAGGGTTTAGTTGGGGTTTAGGGTTTAGGGTTTAGGGTTTAGGGTTTGGGGTTTGGGGTTTAGGGTTTAGGGTTTAGGGTTTAGGTTTAGAGGTTTAGGGTTTAGGGTTTAGGGTTTTAGGGTTTAGGGTTTAGGGTTTAGGGTTTAGGGTTTAGGGTTTAGGGTTTAGGGTTTAGGGTTTAGGGTTTAGGGTTTAGGTTTGGGTTTAGGGTTTAGGGTTTAGGGTTTAGGGTTTTTAGGGTTTGGGGTTTAGGGTTTAGGGTTTAGGGTTTAGGGTTTAGGGTTTAGGGTTTAGGGTTTAGGGTTTAGGGTTTAGGAGGGTTTAGGGTTTTTAGGGTTTAGGGTTTAGGAGGTTTAGGGTTTAGGGTTTAGGGTTTAGGGTTTAGGGGTTTAGGGTTTAGGGTTTGGGGTTTAGGGTTTAGGGTTTTTAGGGTTTAGGGTTTAGGGTTTAGGGTTTAGGGTTTAGGGTTGGGTTCAGGGTTTAGGGTTTAGGGTTTAGGGTTTGGGTTTAGGGTTTAGGGTTTAGGGTTTAGGGTTACAGGGTTTAGGGTTTAGGGTTTAGGGTTTAGGGTTTAGGGTTTAGGGTTTAGGGTTTAGGGTTTAGGGTTTAGGGTTGGGTTTAGGGTTGGGTTTAGGGTTTAGGGTTTAGGGTTTAGGGTTTTTTAGGGTTTAGGGTTTAGGAGTTTAGGGTTTAGGGTTTAGGGTTTAGGGTTTAGGGTTTAGGGTTTAAAATTTAAAATTTAAAATTTAAAAAATTTAAAATTTAAATTTAAAAAATTTAAAATTTAAAATTTAAAATTTAAAATTTAAAATTTAAAATTTAAAATTTAAAAATTTAAAAATTTAAAATTTAAAATTTAAAATTTAAATTTAAATTTAAAATTTAATTAAAATTTAAAATTTAAAATTTAAAATTTAAAATTTAAAATTTAAAATTTAAAAATTTAAAATTTAAAATTTAAATTTAAGAATTTAAAATTTAAAAATTTAAAATTTAGGTTTAAAATTTAAATTTAAATTTAAAATTTAAAAAATTTAAAATTTAAAATTTAAAATTTAAAATTTAAAATTTAAAAATTTAAAATTTAAAAATTTAAATTTAAAATTTAAAATTTAAAAATTTAAAATTTAAAATTTAAATTTAAAATTTAAAATTTAAAAAATTTAAAATTTAAAATTTAAAAATTTAAAAATTTAAAATTTAAAATTTAAAAATTTAAAAATTTAAAATTTAAAATTTAATTTAAATTTTAAAATTTAATTTAAATTTAAAAATTTGAAATTTAATTTAAAAATTTAAAATTTAAAATTTAAATTTAAAAATTTAAAATTTAAAATTTAAAATTTAATTTAAAAATTTAAATTTAAAATTTAAAATTTAAAGAATTTAAAATTTAAAATTTAAGAATTTAAAATTTAAAATTTAAAATTTAAAATTTAAAATTTAAAAATTTAAAATTTAAAATTTAAAATTTAAAATTTAAAATTTAAAATTTAAAATTTAAAAATTTAATTTAAATTTAAATTTAAATTCTAAATTTAGGGTTGGGGTTTAGGGTTGGGGTTTAGGGTTTAGGGTTTAGGGTTTAGGGTTTAGGGTTTAGGGTTTAGGTTTAGGGTTTAGGGTTTAGGGTTTAGGGTTTAGGGTTTAGGGTTTAGGGTTTTTAGGGTTTAGGGTTTAGGGTTTAGGGTTTAGGAGGTTTAGGGTTTAGGGTTTAGGGTTTAGGGTTTAGGGTTTAGGGTTTAGGGTTTAGGGTTTAGGGTTTAGGGTTTGGGTTTAGGGTTTAGGGTTTAGGGTTTAGGGTTTAGGGTTTAGGGTTTAGGGTTTAGGGTTTAGGGTTAAAATTTAAATTTAAAATTTTACAAAATTTAAAATTTAAAATTTAAAATTTAAAAATTTAAATTTAAATTTAAAAATTTAAAATTTAAAATTTAAAATTTAAAATTTAAAATTTAAATTTAAAATTTAAATTTAAAAATTTAAAATTTAAAATTTAAAATTTAAAATTTAAAATTTAAAATTTAAAAATTTAAAATTTAAAATTTAAAATTTAAAATTTAAAAATTTAAAAATTTAAAAATTTAAAATTTAAAATTTAAAATTTAAATTTAATTTAAAATTTAAAATTTAGGGTTTAGGGTTGGGTTCAGGGTTTGGGGTTTAGGGTTTAGGGTTTAGGGTTTAGGGTTTAGTTTAGGGTTTAGGGTTTAGGGTTTAGGGTTTAGGGTTTTAGGGTTTAGGGTTTAGGGTTTAGGGTTTAGGGTTTAGGGTTTAGGGTTTAGGGTTTAGGGTTTAGGGTTTAGGGTTTGGGGTTTAGGGTTTAGGGTTTGGGTTTAGGGTTTAGGGTTTAGGGTTTAGGGTTTTTAGGGTTTAGGTTTAGGGTTTAGGGTTTTTAGGGTTTAGGGTTTAGGGTTTAGGGTTTAGGGTTTAGGGTTTAGGGTTTAGGGTTTAGGGTTTAGGGTTTAGGGTTTTTAGGGTTTAGGGTTTAGGGTTTAGGGTTTAGGGTTTAGGGTTTAGGGTTTAGGGTTTAGGGTTAGGGTTTAGGGTTTAGGGTTAGAGCATTTAATGCTTAGCTGGAATTAAGGCTTATTGACTTTTGGCTCTCCATGTTGGGCCAGTTTTTCTTGTCTTTGGTGTGTGTTAATTTGCTGCTCCTTTCCTATGAGGAGTGGAGAGCAAACTAGAAATAAAAATTAGTTTTGAAGTTTGAAAAAAACAGCGAGTTTCATGGTTATGCTTTGAGTTTGTCTCGAGTTTAAACTTCCCTTATTGCTTTGAGTTAGTTCTTGAGGGATAAGCTTAGGAGATTGAGTGAGTCTTGTCTCTGGCCCTCGACCCAAATCATTGAGTGAGTCTTGTGGTTTTGGTTCTATCATTTTGTTTAGTTTCGGGTTGTGTTAATTGATTGCGTGAGCAGTATCTCTTCTTTAATTCAAATCCATCAAAATAAATTTAGAGGAGTTAACATTTGCATTTACATCATCGGTATCGAGCTTAGGTAAGGATTTTCGTGAGGAAAATCGCGTTTAGATAGTTTTGAACAAACCGGATTCGGTCAGGTTTGGAGTTGCGGTTTGTAAAGGCGATAACTCCTTAGTTCTCATCCAAATGGAGTGATCTTTTTTTTCAATTTGGGTATTTTTCTGTGTAGAATACTCATGAAAAATTTCAAATTTGGAATTTTTACTATTAAAAGGGCCAGGTTTCTCGACGGCGAAAGAGAGGCAGAAACTAGGAAGTCGATTTGAGCGGAAGATTTCTCCTTCATCTGAAGAGCTTTTGAGGAGTTTCTTTTTTCAAATTGATTGTTTTGATCTGAATTTTCTAGTTGTAAACCTTTGAGAGTCTCGAGGCAATTTTTCATTCTGCTTTTGTCTCTAATTTCAGCAGTTTTCAATCCTTGTAATTGCGTAATAGCTAGCAAATAGTGGAAAATCATGGCCAGTGAGAGGTTTCTAGAATGTTGAAAACAATTACTGACAAATTGGAGAGTTTGGAGGGAAAAACAGAAGAGAGTAGGGAGGAGAAGGCTAGGCGAAAATACTAGGATGGATCACGATTCACTTGAGAGAAAGTAGTGTTGCAAAACAACTAATTAGTAGGAAAATGAGGATGTGAGTAGTAGGAGAAATCTTAGGCAGTTTGATGAAGATAGGGGACTAGATTTGACCTACGGGGTGATAGAGCATGATGGTGAGGCCTTTCTAGATTGGGTGAGGCAAATTGAAACCGTTTTTGATATAAGGGGTTTGATGATAAAAAAAGATTCAAATTGTCTGTGCTTAAACTCCCACTAAACTCGCTGCCCTATGGTATGAAAACCTCAAGGCAAAAAGGAGAGAGAGGAAAGAAAAGAAAATTGACTCATGGAGGCAAAAAGCCAACAGTTTTCCCTAATTCTAGCTAAGCATTAAATGCTCTAACCTAAACCTAAACCCTAAACCTAAACCTAAACACAAACTAAACCCTAAACCCTAAACCCCATACAAACTACAAACCACAAACTAAACCTAAACCAAACCTAAACCTAAACCTAAACTAAACCTAAACCTAAACACAAAAAACCTAAACCTAAACCTGACCACAAACCAAACCACAAACCCTAAACCTGACCTAAACCTAAACCCTAAACCTAAACCCTAAACCTAAACCCTAAACCTAAACTACTAAACCCTGGGCCCTGGGCCTGGGCCCTGGGCCTGGGGCCTGGGCCTGGGCCTGGGCCTGGGCGCAGACCTGGGCCTGGGCCTGGTGCGGGGCCTGGAGGCCTGGGCCTGGGCCACGGACCTGGGCCTGGGCCCTGGGCCTGGGCCTGGGCCCCTGTTTCAGGCCCTGGGCCCTGGGCCCCTGGGGCCCTGGGCCTGGGCCTGGGCCGGCCTGGGCCCTGGGCCCTGGGCCTGGATACTGGGCCCTGGGCCCTGGGCCTGGGCCTGGGCCTGGGCCCTGAATGGGTACTGGGGCCCTGGGCCTGGGCCTGGGCTGCTGGGCCTAGATACAGACCTGGGCCTAGACCTGGGCCTGGGCCTGGGCCTGGGCCTGCAGGCCCTAGGCCTGGGCCCTGGGCCTGGGCCTGGGCCTGGGCCCTGGGCCCTCGGCCTGGGCCTGGGCCTGGGGCCTGGGCCTGGGCCCTGGGCCCGGCCTGGGCCCTGGGCCTGGGCCCTGGGCCTGGGCCTGGGCCTGGGGCCTGGGCCTGGGCCTGGGCCTGGGCCTGGGCTGGGCCAGGCCTGGGCCTGGGCCTGGGCTGGAGCCTGGGCCAGGCCTGGGCCTGGGCCCTGGGGCCCTGGGCCTGGGCCTGGGCCTGGGCCTGGGCCTGGGCCCTGGGCCTGGGGCCCTGGGCCTGGGCCCTGGGCCCTGGGCCCTGGGCCCTGGGCCCTGGGCCTGGGTGACTGGGCCTGGGCCTGGGCCCTGGGCCCTGGGCCTAAGATACTCAGGCCCTGGGCCCTGGGCCTGGGCCTGGGCCTGGGCCCTGAGCCTGGGCCTGGGCCGGCCTGGGCCTGGGCCACAGGCCCTGGGCCTGGGCCTGGGCCTGGGCCTCCTGGCCTGGGCCCTGGGCCCTGGGCCCTGGAGCCTGGGCCCTGGGCCCTGGGCCTGGGCCCTGGGCCTGGGCTCAGGCCCTGGGGCCCTGGGCCCTGGGCCTGGGGCCCTGGGCCCTGGGGCCTGGGCCCTGGGCCTGGGCTGTTTTCAGGCCTGAATACTGGGCCCACAGGCCTGGGCCTGGGCCTGGGCCTGGGCCTGGGCCTGGGCCCTGGGCCTGGGCCCTGGGCCCTGGGCCTGGGCCCTGGTATGGTAGCAGGCGCAGGCGCAGGCGCAGGCGCAGGCGCAAGTATAAGTAGACCTGGTGCAGGCCACTGAAGTGCTGGACCTCGTAGGCCCTGGGCCACCAGGCCACAGGCCTGGGCCTGAGGCCTGGGCCAGGCCCTGGGCCCTGAGCCTGACCTGGGTGCTGGGCCTGGGCCTGGGCCTGGGTGGGCCCACTGGGCCCTGGGCCTGGGGCCTGGGGCCTGGGCCTGGGCCTGGATTTGGGCCTGGGCCTGGGCCCTGGGCCCTGGGTGGGCCCTGGGCCCTGGGCCCTGGGCCCTGGGCCTGGACCATTACTGAATACTGGGCCCTGGGCCTGAGCCTGGGCCCTGGGCCCTGGGCCTAGACCTCAGGCCTAGGCCTGGGCCTGGGCCCTGGGCCCTGGGGCCTGGGCTGGGCCACAGGCCTGACCTCCTGGGCCCTGGGCCCTGGGCCTGGGCCACTGGGCCACTGGGCCTGGGCCTGGGCCCTGGGCCCTGGGCCTGGGCTTGCTGGGCCCTGGGCCCTGGGCCTGGAGGCCTGGGCCCTGGGCCTGGGCCTGGGCCCTGGGCCCTGGGCCTGGGCCTGGGCCCTGGGGCCTGGGCCCTGGGCCTGGGCCCTGGGCCCTGGGCCACTGGGCCCTGGGCCTGGGCCTGGCACTCAGGCCCTGGGCCTGGGGCCCTGGGCCTGGGCCCTGGGCCCTGGGCCCTGGGCCTGGGTACTGAATACAGGCCCTGGACCCTAGGCCTAGATACGACCCTAGGCCCAGGCCTGGGCCTAGGCCCTGGGCTGGGCCACTGGGCCAGGCCCTGGGCCCTGGGCCCTGGGCCTGGGCCTGGGGCCTGGGCCTGGGCCTGGGTACTGAGGCCCTGGGCCTGGGCCACAGGCCCTGGGCCTGGGCCTGGACCTGGGCCCTGGGCCTGGGCCCTGGGCCTGGGCCCTGGGCCCTGGGCCTGGGCCTGGCCTGGGCCTGGGCCTGGGCCCTGGGCCTGAATTTTTGGGCCTGAATGCTGGGCCCTGGGCTGGGCCTGGGCCACCTGGGCCCTGGGCCCTGGGCCTGGGCTACTGGGCCCTGGGCCCTGGGCCCTGGGCCCTGGGCCTGGGCCCTGGGCCTGGCCTGGGCCCTGGGCCCTGGGCCCTGGGGCCTAGGCCCTGGGCCCTGGGCCACTGAGCCAGGCCTGGTACAGGCCTGGGCCCTGGGCCCACAGGCCTGGGCCCTAGGCCACCAGGCTGGGCCACTGGGCCTGGGCCCTGGGCCCTGGGCCCACCCAGGCCCTGGGCCCTGGGCCCTGGGCCACAGGCCCTGGGCCCTGGGCCCTGGGCCACTGGGCCCTGGGGCCTGGGCCCTGAGGCCCTGGGCCCTGGGCCTGAATGCTGGGCCACTGGGCTGCTGGGCCCTGGGCCCTGAATGCTGGGCTACTGGGCCTGGAGCCTGGGGCCCTGGAGCGCCTGGGCCTGGGCCACTCAGGCCCTGGGCCCTGGGCCTGGGCCTGGGCCTGGGCCCTGGGCCTGGGCCTGGGCCTGGGCCACACAGGCCCTGGGCCTAAAATGCTGGGCCCTGGGCCACTGTGAATACACAGGCCCTGGGCCGACCTCAGGCCTGGGCCTCCAGGCCCTGGGCCCTGGGGCCCTGGGCCTGGTGCAGGCCCTGGGCCTGGGCCTGGGCCCTGGGCCCTGGGCCCTGGGCCACTGGGCCTGGGCCCTGGGCCTGGGCCCTGGGCCTGGGCCTGGGCCCACTGGGGCCCTGGGCCCTGGGCCTCAGATACTGAATACTGGGCCTAAGGCCTGGGCCTGGCCTGGGCCTGGGCCTGAATGCAAGGCCTAGACCCTGAATACTGGGCCTGGGCCCTGGGCCTGGGCCTGGGCCTGGGCCCTGGGCCTGGAGCCTGGGCCCTGGGCCCTGGGCCTGGGCCTGGGCCCTGGGCCTGGGCCTGAATGCCTGGGCCTGAATACAGGCCTGGGCCATGGCCTGGGCCTGGGCCTGGGGCCCTGGGCCTGGGCCTGGTGCAGGCCTGGGCCTGGGCCCTGGGGCCCTGGGCCTGGGCCCTGGGCCTGGGCCCTGGGCCTGGGCCTGGGCCCTGGGCCTGGATGGGCCTGGGCCTGGGTGTGGTGCTGGTGCACAGGCCCTGAATGGGCCCTGGGCCCTGGGCCTGGGCCACCTGGAGCCCTGGGCCCTGAATGCTGGCCCACTGGGCCCTGGGCCTGGGCCCTGGGCCTAGATACTGGGGCCCTGGGCCAGGCCCTGGGCCCTGGAGCCACTGGCCACTGGGGCGGCCTGGGCCACTAGCCTGGGCCCTGGAGCCTGGGCCCTGGGCCCTGGGCCCTGGGCCCTGGGCCTGGGCCTGGGCCCTGGGCCTGGGCCTGAATACTGAATGCTGAATACTGAATACTGGGCCCTGGGGCCTGAATACAGGCCTGGATGGAATACTGAATACTGGGCCTAGATGCACAGGCCTGGACCTGGGCCTCAGATATGGGCCCTGAATACTGGGCCTGGGCCTGGCCTGGGCCTGGGCCCTAGTACTGGGTACAAGGCCTGAATACTGAATGCTGAATACTAAGACCTGGGTACTTTGGACCCTGTTATGGGCCTGGGCCCTAGACCTAGGCCCTGGGCCTGGGCCTGGGCTGCTGGGCCTGGGCCTGGGCCCTGGGCCACTGGGCCCAGGCCCTGGGGCCTGGGCCTGGGCCCTGGGGCCTGGGCCTGGGCCTGGGCCACTGGGCTGGGCCTGGGCCTGCCTGAGGCCTGGGACCTGGGCCCTGAATACTGGCCACTGGAGCCTGCTGGGCCCTGGGCCTGGGCCCTGGGCCTGGGCCCTGGGCCTGGGCCCTGGGCCTGGGCCTGGGCCTGGGCCCTGGGCCTGGGCCTGGGCCTGGGGCCCTGGGCCCTGGGCCCACTGGGGCCCTGGGCCTGGGCCTGGAATGCTGGGCCCTGGGCCCTGGGCCTGGGGCCTCAGATATAATTTCTGGGCCTGGCCTGGGGCCTGGGCCACTGAGGCCTGGGCCTGGGCCCTGGGCCTGGGCCTGGGCCACTGAGCCCTGGGCCCTGGGCCCTGGGCCTGGGCCCTGGAGCCCTGAGGCCTGGGCCTGGGCCCACTGGAGGTGCTGGGCTGGGCCTGGGCCTGGGCCTGGGCCAGGGCCCTGGGCCTGGGCCCTGGTGCTGGGCCCTGGGCCTGGGCCTGGGCCTGGGCCTGGGCCCTGGGCCCTGGTGACCTGAATGGGCCCTGGGCCTGAATGGGCCCTGAATGCACTGGGCCTGGGCCTCAGGCCCTGGGCCCTGGTGCTGGGTGGGCCCTGGGCCTGGGCCTGGGCCATGGGCCTGGGCCTGGGCCTAGACCTGGGCCTGGGCCTGGGCCCTGGACCACTGGGCCTGGGCCTGGCCTGGGCCTGGGCCTGGACCTGGGCCCTGGGCCTAAGGCCTCAGGCCCTGGGCCCTGGGGCCTGGGCCCTGGGCCCTGGGCCCTGAATGCTGGGCCTGGGCCTGGGTGCTGATGCTGGGCCCTGGGCCCTGGGCCCTGGGCCTGGGCCCTGAATGCTGAGATACAGGCCCTGGGCCCTGGGCCACTGGACCACAGGCCTGGGCCTGGGCCTGGGCCTGGATACCTGGGCCCTAGACATGGGCCTGGGCCTGGGGCCTGGGCCCACTGGGCCCTGGGCCCTGGACCTGGGCCTGGGCCTGGGCCCTGGATATAGATACTGGGCCTGGGCCTGGGCCTGAATTTGGGCCTGAATACAGACCTAGATACTGGGCCTAGACCTAGATATAGATATAGATACAGACCTAGGCCATAGACCTAGACCTGGAGCCTGGGGCCCTAGGCCTGGGCCTGGGCCCTGGGCCTGGGCCTGTTTGGGCCCTGGGCCTGGGCCTGGGCCACTGGGCCACCTGGGCCTGGGCCTGGGCCTGGGGCCTGGGCCCTGGGCCCTGGGCCTGGGCCTGGGCCTGGGTGTGGGCCCTGGGCCTGGGCCCTGGGCCCTGGGCCTGAATACAGATATGGGCCTGGGCCCTGGGCCCTGGGCCTGGGCCACTGAATTTTTGGGCCCTGGAATGCTGAACCACTGGGCCTGGGCCTGGGCCTGGGCCTGGACCTGGGCCCTGGGCCTAGGCCTGGAGCCCTGGGCCTGGGCCTGGGCCCTGGGCCTAGATATGGAGCCTGGGCCTGGACCCTGGGCCCTCTGAGCCACCAGGCACGTGCTGGCCAGGCCCTGGGCCCTGGGCCCTGGGCCTGAGCCCTGGGGCCCTGGGCCCTAGATGCAGGCCCTGGGCCTGGGCCTGGGCCCTGGGCCCTGGGCCTGGGCCCTGGGCCGGGGCCTGACCTGGGCCTGGGCCCTGGGCCCTGGGCCTGGGCCACTGGGCCTGGGCCCTGGGCCACTGGGCCCTGGCCTAAGACACTGGAGCCCTGGGCCCTGGGGCCCTGAATACTGGGCCCTGGGCCTGGGCCCTGGGCCTGGGCCTAAAGACCTGGGCTACTGGGCCTGGGCCTGGACCTGGGCCCTGGGCCTGGGCCTGGGCCACTGGGCCTGGGCCTGAATGGGCCCTAGACCTGGGCCTGGAATATAGACCCTGGGCCCTCAGGCCCTGGGCCCTGGGCCCTGGGCCTAAGGCCTGGGCCTGGGCCCTGGAGCCTGGAGTACTGAGTACAGGCCTGGGCCTGGGCCTGGGCCTAGACACAGACCCAGGCCCTGGAATACTGGGCCCTAGACCCTGGGCCTGGGCCTGGACCTGGGCCTGGGCCTGGGCCCTGGGCCCTGGACCCTGGGCCTGGGCCTGGGCCTAGACCTGGGCCTGAATGCCTGAATGGGCCTGGGCCTGTTTGGGCCTGGGCCCTGGGGCCCTGGGCCCTATGGATACTGGGCCTAGGCCTGGGCCCTGAATGATGCGAAGTGAAGAAGGCT

**Data S5** Four cases of ITR-DTR junctions. Approximately 1000 bp read fragments are shown.

Variants:

ITRs: TTTAGGG, TTTAGG and TTAGGG (the latter of small percentage ≈2-5%) (yellow)

DTRs: CCTGGG, CCCTGGG, CCTAGG (green)

*C. iljinii* 433-9 read #13986


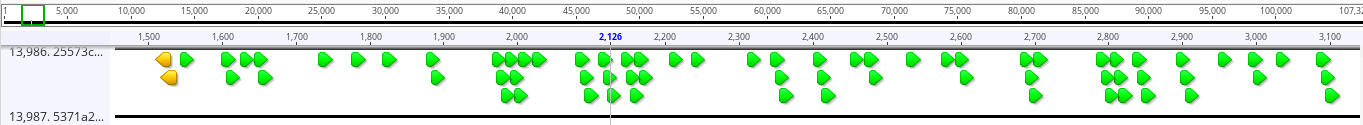


CCTAAACCTAAACCCCTAAACCTAAACCTAAACCTAAACCTAAACCCTAAACCCTAAACCCTAAACCCTAAACCTAAA↓CCTGGGCCTGGGCCTGGGCCTAGACCTG

GGCCCTGAGCCTGGGCCCTGGGCCCAGGCCTGGGCCTGGGCCTGGGCCTGGGCCCTGGGCCTGGGCCTGGGCCCTGGGCCTGGGCCTGGGCCTGGGCCTGGCCTGGGCCTGGGCCCTGGGCCTGGGCTGGGCCTGGGGCCTGGGCCTAGGCCTGGAGGCCCTGGGCCTGGGCCTGGGCCCTGGGCCTAGGCCCTGGGCCTGGGCCCTGGGCCTGGGCCTGGGCCTGACTGGGCCTGGAGGCCTGAGCCTGGGCCTGGGCCTGGGCCCTGGGGCCTGGGCCTGGGCCCTGGGCCTGGGCCCTAGGCCCTGGGCCTGGGCCTGGGCCTGGGCCTGGAGGCCTGGGCCCTGGGCTGCCTGGGCCCTGGGCCTGGGGCCTGGGCCCTAGGCCTGGGGCCCTGGGCCTGGGCCTGGGCCTGGGCCTGGGCCTGGGCCTGGGCCTGGGCCTGGGCCCTGGGCCTGGGCCTGGGCCCTGGGCCCTGGGCCTGGGCCCTGGGCCTGGGCCTGGAGCCTGGGCCTGGGCCTGGGCCTGGGCCTGGGCCCTGGGCCTGGGCCTGGGCCTGGGCCTGGGCCCTGGGCCTGGGCCTGGGCCTGGGCCTGGGCCTGGGCCTGGGCCTAGGCCCTGGAGGCCCTGGGCCCTGGGCCTGGGCCTGGGGCCCATCTGGGCCTGGGCCTGGGCCTGGGCCCTGGGCCTGGGCCCTGGGCCTGGGGCCACAGGCCTGGGCCCTGGGCCTGGGTACGGCCTGGGCCTGGGCCTGGGCCCTGGGCCTGGGCCCTGGGCCTGGGCCTGGGCCTGGGCCTGGGCCCTGGGCCTGGGCCCTGGGCCTGGGCCCTGGGCCTGGGCCTGGGCCTGGGCCTGGGCCCTGGGCCCTGGGCCTGGG

*C. pamiricum* 830-3C read #2613


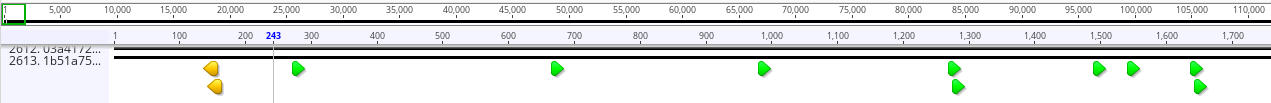


CCCTAAACCCCTAAACCTAAACCCTAAACCCCTAAACCCTAAACCCCTAAACCCTAAACCCTAAACCCTAAACCTAAACCCTAAACCCTAAACCCCTAAACCCTAA↓GACCTGGGCCCTGGGCCACTGAGGCCCTGGGCCACTGGGCCTGGGGCCCTGGAGGCCTGGCCTGGGCCATGGGCCCTGGGCCTGGGCCTGGGCCCTGGGCCTGGGGCCCTGGGCCCTGGGCCCTGGGCCCTGGGCCCTGGGCCTGGGCCCTGGGCCACTGGAGCTGGGCCCTGGGCCTGGGGCCCTGGGCCTGGGCTGGCCTGGGCTGGGCCTGGGCCCTGGGGCCCTGGGCCCTAGGCCCTGGGCCTGGTACCTGGGCCCTGGGCCCTGGGCCCTGGGGCCTGGGCCTAGGCCTGGGCCCTGGGCCCTGGGCCTGGGCCCTGGGGCCTGGGCCCTGGGTGCTGGGCCCTGGGCCTGGGCTTGCTGGGCCTGGGCCTGGCCTGGGCCCTGGGCCTGGGCCCTGGGGCCCTGGGCCTGGGCTGGGCCACTAGGCCCTAGGCCCTGGGCCTGAGGCCCTAGGCCACAGGCCCTGGGCCTGGGCCTGGGCCACAGGCCCTGAGCCTGGGGCCTGGGCCCTGGGCCCTGGGGCCTGGGGCCTGGGGCCCTGGGCCCTGGGCCCACACACAGGCCCTGGGCCCTGGGCCCTGGGCCTGGGCCCTGGGGCCCTGGGCCCTGGGCCCTAGGCCCTAGGCCCTAGGCCTGGACCTAGGCCCTAGGCCACAGGCCCTAGGCCTGGGCCCTGGGCCTGGGCCCTCAGGCCCTGGGCCTGAGCCCTGGGCCTAGGCCCTGGGCCTGGGGCCCTGGGTATTGCTGAGGCCCTGGGCCCTGGGCCTGGGCCACAGGCCTGGGCCTGGGCCTGGGCCCTAGGCCTGGGCCCTGAGCCTAGGCCTGGAGGCCCTAGGCCTAGTACTGAGCCCTGGG

Read # 21993


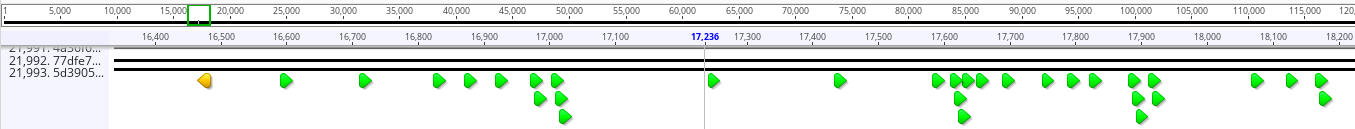


CCCTAAACCCTAAACCCTAAACTAAACCTAAACCTAAACCTAAACCCTAAACCTAAACCCTAAACCCTAAACCTAAACCCTAA↓GCTGGGCCACTGGGCCTGGGCCTGGGCCCTGGGCCTGGGCTGGGCCTGGGCCTGGGCCTGGGCCCACTGACCTGGGCCTGGGCCCTGGGCCCTGGGCCCTGGGCCCTGGGCCCTGGGCCCTGGGCCTGGTGCTGGGCCTGGGCCCTGGGCCCTGGGCTGCTGGGCCTGGGCCTGGGCCTGGGCCACTGGGCCCTGGGCCCTGGGCCCTGGGCCCTGGGCCCCTGGGTACGGACCTTTGGGCCCTGGGCCCTGGGCCCTGGGCCCTGGGCCACGGGGACCTGGGCCTGGGCCTGGGCCCTGGGCCCTGGGCCTGGGCCCTGGAGCCCTGGGCCTGGGCCTGGGCCCTGGAGCCTGGGTGCTGGGCCTGGGGCCCTGGGCCTGGGCCTGGGCCCTGGGCCCTGGGCCCTGGGCCCTGGGCCTGGGCCCTGGGCCTGGGCCTGGGCCTGGGCCCTCGGCCCTGGGCCTGGGCCTGGGCCTGGGCCTGGGCCTAGGCCCTGGGCCACTGGGCCCTAAGGCCTAGGCCTGGGCCCTGGGCCCTGGGCCACAGGCCCTGGGCCACTGGGCCCTGGGCCTGGGCCCTGGGCTGGGCCACAGGCCCTGGGCCTGGGCCCTGGGCCTGGGCTGCGTACTGGGCCTGGGCCACTGTGGGCCTGGGCCTGGGCTCTGGGCTGGGCCTGGGCCTGGGCCACTGGGCCCTGGGCCTGGGCCTGGGCCCTGGGCCTGGGCCTCAGGCCCTGGGCCTGGGCCCTGGGCCTGGGTACTGGGCCACTGTTTGGGGCCTGGGGCCCTGGGCCCTGGGCCCTGGG

*C. acuminatum* 429-3 Read #18036


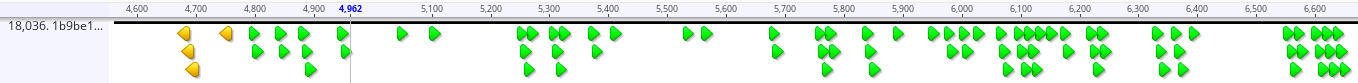


CTAAACCCCTAAACCTAAACCTAAACCTAAACTAAACCTAAACCCCTAAACCTAAACCTAAACCCCACAAACCTAAACCTAAAACCTAAACCCTAAACCCTAAACCCTAAACCCTAAACCCTAAACCTAAACCTAAACCCTAAACCTAAACCCTAAACTCGCCCTAAACCCTAAACCCTAAACCTC↓GCCCTAAGCCTAAACCTAAACCTAAGCCTGGGCCTGGGCCTGGGCCTGGGCCCTGGGCCTGGGCCCTGGGCCCTGGGCCTGGGCCTGGGCCTGGGCCCTGGGCCCTGGGCCCTGGGCCTGGGCCTGGGCCTGGGCCTGGGCCTGGAGGGGCCTGGGCCTGGGCCCTGGGCCTGGGCCCTGGGCCTGGGCCTGGGCCTGGGCCCTGGGCCCTGGGCCACAGGCCTGGGCCTGGGCCTGCGGGCCCATGGCCTGGGCCACTGGGCCCTGGGCCCTGGGCCCTGGGCCTGGGCCTGGGCCCTGGGCCCTGGGCCTGGGCCCTGCAGGCCCTGGGCCCTGGGCCTGGGCCTGGGCCACTGGGCTGGGCCTGGGCCTGGGCCCTGGGCCACAGGCCACGGGCCTGGGCCTGGGCCACTGGGCCTGGGCACTGGTGGCCACTGAGGCCCTGGGCCCTGGGCCCTGGGTACTGGGCCCTGGGCTGGGCCCTGGGCCTGGGCCTGGGCCTGGGCCTGGGCCTGGGCTGGGCCTGGGCCTGGGCCCTGGGCCTGGGCCTGGGCCTGGGCCTGGGCCTGGGGCACTGGGCCCACTGGGCCTGGGCCACTGGGCCTGGGCCTGGGCCTGGGCCTGGGCCCTGGGCCTGGGGCCTGGGCCTGGGCCTGGGCCCACTGGTGCAGGCCCTGGAGCCTGGGCCCTGGGCCCTGGGCTGGGCCACAGGCCCTGGGCCTGGGCCCTGGGCCTGGGTACTGGGGCCCCACTGGGCCTGGGCCCTGGGCCTGGGCCTGGGCCCTGGGCCAGGCCCTGGGCCTGGGCCTGGGCCCTGGGCCTGGGGCCCTGGGCCCTGGGCCCTGGG

**Data S6** Example of telomere sequences array of *Arabidopsis*-type with monomers elongation

*C. pamiricum*, accession 177, read 10528 (11922 bp)

TRF analysis:

Sequence: 177_10528

Parameters: 2 7 7 80 10 50 500

| Indices | Period Size | Copy Number | Consensus Size | Percent Matches | Percent Indels | Score | A | C | G | T | Entropy (0-2) |
| --- | --- | --- | --- | --- | --- | --- | --- | --- | --- | --- | --- |
| 2849--2974 | 33 | 3.6 | 33 | 76 | 14 | 103 | 23 | 1 | 35 | 38 | 1.65 |
| 2740--2982 | 17 | 13.8 | 17 | 67 | 17 | 88 | 23 | 2 | 36 | 37 | 1.67 |
| 3101--3171 | 19 | 3.7 | 19 | 69 | 23 | 67 | 19 | 2 | 39 | 38 | 1.67 |
| 3069--3277 | 112 | 1.9 | 106 | 83 | 8 | 258 | 25 | 1 | 36 | 35 | 1.67 |
| 3175--3288 | 17 | 6.4 | 17 | 69 | 17 | 52 | 30 | 1 | 33 | 34 | 1.68 |
| 3259--3313 | 17 | 3.2 | 17 | 81 | 0 | 65 | 27 | 1 | 36 | 34 | 1.68 |
| 3267--3367 | 17 | 5.6 | 17 | 72 | 15 | 69 | 24 | 0 | 37 | 36 | 1.63 |
| 3072--3343 | 107 | 2.5 | 109 | 82 | 9 | 288 | 26 | 1 | 35 | 35 | 1.68 |
| 3356--3385 | 15 | 2.0 | 15 | 100 | 0 | 60 | 26 | 0 | 40 | 33 | 1.57 |
| 3300--3581 | 71 | 4.0 | 70 | 70 | 21 | 230 | 23 | 2 | 35 | 38 | 1.69 |
| 3250--3582 | 142 | 2.4 | 138 | 79 | 12 | 331 | 24 | 2 | 35 | 38 | 1.68 |
| 3306--3581 | 70 | 3.9 | 68 | 77 | 12 | 231 | 23 | 2 | 35 | 38 | 1.68 |
| 3664--3698 | 7 | 5.0 | 7 | 100 | 0 | 70 | 14 | 0 | 42 | 42 | 1.45 |
| 3869--3901 | 17 | 1.9 | 17 | 93 | 0 | 57 | 24 | 3 | 39 | 33 | 1.71 |
| 3727--3986 | 57 | 4.7 | 56 | 72 | 15 | 164 | 23 | 1 | 37 | 37 | 1.66 |
| 3727--4048 | 167 | 2.0 | 163 | 83 | 9 | 404 | 22 | 1 | 36 | 38 | 1.66 |
| 3995--4500 | 35 | 13.8 | 38 | 68 | 17 | 222 | 24 | 1 | 37 | 35 | 1.67 |
| 4148--4500 | 38 | 9.6 | 36 | 69 | 17 | 194 | 24 | 2 | 37 | 35 | 1.68 |
| 4319--4501 | 73 | 2.5 | 75 | 81 | 7 | 216 | 24 | 2 | 36 | 36 | 1.68 |
| 4396--4521 | 35 | 3.5 | 35 | 71 | 18 | 107 | 23 | 2 | 34 | 39 | 1.68 |
| 4396--4651 | 70 | 3.6 | 72 | 72 | 20 | 200 | 24 | 2 | 35 | 37 | 1.69 |
| 4323--4636 | 70 | 4.4 | 69 | 72 | 21 | 195 | 25 | 2 | 35 | 36 | 1.68 |
| Indices | Period Size | Copy Number | Consensus Size | Percent Matches | Percent Indels | Score | A | C | G | T | Entropy (0-2) |
| 4590--4734 | 51 | 2.7 | 53 | 76 | 17 | 154 | 24 | 1 | 40 | 34 | 1.64 |
| 2740--11921 | 18 | 506.3 | 18 | 64 | 20 | 1149 | 24 | 2 | 36 | 36 | 1.69 |
| 5018--5078 | 19 | 3.0 | 20 | 72 | 13 | 52 | 22 | 1 | 40 | 34 | 1.64 |
| 5388--5508 | 35 | 3.4 | 35 | 70 | 21 | 99 | 24 | 3 | 34 | 37 | 1.72 |
| 5592--5626 | 7 | 5.0 | 7 | 100 | 0 | 70 | 14 | 0 | 42 | 42 | 1.45 |
| 5664--6860 | 55 | 21.9 | 54 | 67 | 21 | 316 | 23 | 1 | 36 | 37 | 1.66 |
| 5647--6863 | 112 | 11.1 | 108 | 68 | 18 | 317 | 23 | 1 | 36 | 37 | 1.66 |
| 6101--6167 | 20 | 3.4 | 20 | 82 | 10 | 93 | 23 | 1 | 41 | 32 | 1.64 |
| 6090--6430 | 40 | 9.0 | 38 | 71 | 18 | 230 | 24 | 1 | 38 | 35 | 1.65 |
| 6268--6488 | 75 | 3.0 | 77 | 82 | 13 | 238 | 22 | 1 | 39 | 37 | 1.63 |
| 6382--6569 | 71 | 2.6 | 68 | 81 | 13 | 202 | 22 | 2 | 38 | 37 | 1.66 |
| 6632--6758 | 53 | 2.4 | 55 | 83 | 12 | 158 | 23 | 2 | 35 | 38 | 1.68 |
| 6600--6824 | 109 | 2.1 | 109 | 78 | 9 | 239 | 23 | 1 | 36 | 38 | 1.66 |
| 6730--6897 | 87 | 1.9 | 88 | 83 | 8 | 218 | 23 | 1 | 38 | 37 | 1.63 |
| 6763--6901 | 51 | 2.6 | 53 | 82 | 10 | 144 | 24 | 0 | 38 | 35 | 1.61 |
| 6829--7131 | 113 | 2.7 | 115 | 79 | 8 | 286 | 26 | 2 | 37 | 33 | 1.69 |
| 6817--7169 | 38 | 9.4 | 39 | 73 | 13 | 228 | 26 | 2 | 37 | 34 | 1.69 |
| 6693--7125 | 73 | 5.9 | 73 | 71 | 15 | 270 | 24 | 1 | 36 | 36 | 1.67 |
| 7090--7168 | 42 | 1.9 | 41 | 92 | 7 | 133 | 30 | 2 | 32 | 34 | 1.71 |
| 7437--7517 | 17 | 4.8 | 16 | 72 | 14 | 54 | 18 | 1 | 40 | 39 | 1.59 |
| 7756--7878 | 56 | 2.3 | 53 | 82 | 12 | 135 | 23 | 2 | 32 | 41 | 1.68 |
| 7722--10760 | 55 | 55.5 | 54 | 67 | 18 | 390 | 24 | 2 | 36 | 36 | 1.70 |
| Indices | Period Size | Copy Number | Consensus Size | Percent Matches | Percent Indels | Score | A | C | G | T | Entropy (0-2) |
| 8193--8303 | 52 | 2.1 | 52 | 85 | 11 | 156 | 25 | 1 | 37 | 35 | 1.67 |
| 8237--8309 | 17 | 4.3 | 16 | 73 | 17 | 58 | 19 | 1 | 38 | 41 | 1.60 |
| 8050--8436 | 195 | 2.0 | 194 | 84 | 9 | 513 | 24 | 2 | 36 | 36 | 1.70 |
| 8515--8981 | 19 | 25.5 | 17 | 64 | 22 | 64 | 23 | 2 | 35 | 37 | 1.70 |
| 8663--8713 | 20 | 2.7 | 18 | 87 | 6 | 75 | 27 | 1 | 33 | 37 | 1.68 |
| 8888--10190 | 73 | 18.0 | 71 | 71 | 15 | 406 | 23 | 2 | 37 | 36 | 1.69 |
| 8827--9881 | 37 | 29.3 | 35 | 68 | 18 | 146 | 24 | 2 | 36 | 36 | 1.68 |
| 9079--9368 | 56 | 5.2 | 54 | 76 | 13 | 238 | 23 | 2 | 37 | 36 | 1.68 |
| 9070--9371 | 111 | 2.7 | 110 | 76 | 15 | 273 | 23 | 2 | 37 | 36 | 1.68 |
| 9502--9567 | 19 | 3.7 | 19 | 73 | 22 | 72 | 19 | 1 | 40 | 37 | 1.61 |
| 9924--9989 | 19 | 3.4 | 20 | 75 | 6 | 64 | 19 | 4 | 40 | 34 | 1.72 |
| 10144--10286 | 53 | 2.7 | 54 | 78 | 8 | 166 | 27 | 2 | 39 | 31 | 1.68 |
| 10217--10273 | 17 | 3.0 | 19 | 77 | 15 | 64 | 29 | 0 | 35 | 35 | 1.58 |
| 10219--10356 | 71 | 1.9 | 72 | 83 | 2 | 179 | 23 | 1 | 39 | 35 | 1.64 |
| 10422--10523 | 35 | 2.8 | 38 | 74 | 11 | 97 | 24 | 2 | 37 | 35 | 1.71 |
| 10998--11147 | 52 | 2.7 | 53 | 78 | 11 | 151 | 25 | 2 | 38 | 34 | 1.70 |
| 11482--11614 | 55 | 2.5 | 53 | 79 | 11 | 137 | 23 | 3 | 38 | 35 | 1.70 |
| 11598--11663 | 19 | 3.6 | 19 | 76 | 10 | 57 | 28 | 3 | 31 | 36 | 1.73 |
| 11612--11780 | 93 | 1.8 | 92 | 83 | 10 | 227 | 23 | 2 | 34 | 38 | 1.70 |
| 11739--11905 | 37 | 4.4 | 37 | 70 | 14 | 117 | 26 | 2 | 35 | 35 | 1.70 |
| 11729--11921 | 55 | 3.4 | 55 | 76 | 12 | 203 | 26 | 2 | 34 | 36 | 1.70 |

Consensus pattern (7 bp):

TTTAGGG

And >1000 alignments of motif GTTTAGG across 66 determined consensus monomers.

Sequence:

>177_read_10528

AACGTGCTTCCGTTTCAGTTACGTATTACTATGGCTTCTTGAGAGTATTACACGTATAACTCTGCTTAGACTTTTTAACCGTTGAAGTTTTCATGTATTTGCTGAAAAGTAGTACTATGATTTAGTTGCTAATTATAAAATTATTTAAGGGTGGTTGACTTTTTGTATGTTTTTGATCGAAATGAAACAAGTGTTTACTTGGTTTTTTTATAAGAAATTAGGCTCCTATGTTGAAATAACCTGCTTCTTCTTTAATACTACGTACGACTTAGTGACATAATTTGCGCCATGCACATCACACTTCCCAACATCAATCCTAAGGCAAAGCAATGTCAAAGTTTAAAAAATGGATGATGAAAGAGCAATAATACTTCATTTTCATACATGAGAATAACTATATTGTTTTGAGGTACAAACATCATGTATTAACGTGCAAGCTACAATGTCATATTCATTCAGAGTTTAAATCTAGATTTAGAAAAGTCTATGAAAAATCGAAGTTGAATAGGCTTTTAAATTCAAAGAATGTAAACTGATGATTAAATCTCACTACCCATAAAGGATAAATCAAATTTATTGCTAGAAAGTGTTTGAATAATTTTCCCTACCTAGCAGTTTCAAAGGAGAATTACTATCAACGACATTAAATCAACTCATTGGAATCACTATCAATGACACTAAATTAAATCAACATATTGCCATATTGGAATCATGAAGAAAACACACTTCCACTATCCCAAGTTTAGCCTAATCGTCAATATAGGTATTTAATTTGTGTGTGACACATTATATATGCATATGGTTTTAACAGCTCAATATAAACGCCATACATTAAAAATGTGTGAACATCATTTCAACACATTATGTTCACGTATTCTGTTTTTTCCAATTTGAGAAGTAGAATGTCTAACTAAGTTATTGACTTATTTCTATATACACGACTCATAACTTTATGATTTACTCTAATAAAGGTTTGCTAAAAATGAATGTTTTAACCAATTACTCGTATGTACTTTGTATACCTTTATCATCATCAAAGTTGTATGTCGCATACATACCAATTCTATTTCCTTTATTGCCTAATAAGCTCTAATCAAATGATTTTATTTGGTAAGCCCTAGCCAGTATGTTAAATAGGAAAATATAATAAACACTAAATTAAAATATACAATTTTTTTTTGAACACATGAATTATTTAAGAAGCATAACATGTTTTCACATACATTTATGTTCTTTTTATTTTCTGTTGCTTGTACAGTATACTAATGTTTGTATCCATGCATGATTTATAGAGCCTAGATTGGAAATGTTAAAACTTAAATCCTAGTTATATGTTGCTATAACAATTATCTAATGTTGAACTTATTAATAATGGTGATCCTAATTAATATTTTCAAAGTAACAGCAACTAATCTAATTATTGGTGCTATGATAAAGTCGTGACATGTTGTTTGGTTTGCCAATTTTTTTGGCGAGATACAAGAACTTTGACGTTTTTGGAAAGTACATTCTAAGAGGGAGTTAAGCTAGGAACTTCGTCCATTATATGTTGGGAAGTACAACACCAATGAAATTGTACTTATAGACTTATTATTGTGAATGAACCAAATAACTACATGCATCATAGTTCAATATTAAATTATTGTGTGCATATATGTCAAATTGTCAACCAATCTATGATTGAGCAATATTTTTATAAAAATATGAGCTACTTTTGAGAAACTAATTTATCCAACTAATTGAGCAATATTTTATAAAATTAGGCTAATTAATTTTGAGAAACTGATTTTATAAAAACGATTTTTATAATATCAGGTCATTCTTGGATTTATAGATATATCTTTTTTTAAAAAACAAAGTATGAATATATATACTCCATATTTTCATATTAAAGTTTTAATAGTTAATATAGGAAAAACTAAAGAAATATTAACATACAAATTGAATGTATAAGAATTGACGAAATATTTTTTTAACAAATCAAATGTACAAGAGTTAATGATAAAAAAAAAAATTAACTCATACTTTTCTTTTAATATGTGAATTTTTTTTTCTTCGTAAATGGTAAGAGGATTCTCTTTGAGCGATGTTTATTATAAGTCGTGACTTACCACAAAGCCAGACTCATCAAAAAGTGAGGAAAAAGGATGTGATAAGGAAAATTCTCACATCCAAGAGCCCCTATTATGGAATTTGATCCTGTCGACCAAACAAGTAAATCTTATTCTATAAGTAAGATAACTTTACTACTAAGCTAAGCACGACCTTACTTGGTTTTAATATAAATATGTGATTATATTAAATGAAAAAATTGAAATGTCTATTAACATCCTCATCTTGTAGGAGCATATAAGCCCTCTAAATTTGATATACAAAGATTTGCAATATATATTGAGGAAAAGGTGCGCACGGTGTGATATGGCTGGAATTTCCAACCTTAAACAGTGATGGCCAACTTCCAAAGATTTGGTACATCACGAAAAGTATATAAAATCATACTATTATCATGCAAAAGGTTGTAAAAGTTTAAATTATTATACTACTTACTATGTGACAAGTTAAATTAAAATTATGATTTTGTGTATATAACCTAAAGGAGAAAAAAGAAGGATAAGAATCTAGCCTGATCACAATAACAGCTACTTTAATTCTACAATAATAGACAAATATCAAATGTACATCACATCTCCATGTCATTATGCACGTACTAGTATTGGGTAGTAGGGAGTTTAGGAGATTTAGTAGGTGTGTAGATGCTAGTAGGGTTTAGTTGATAGTAGGTGTTAGGAGATTTAGTAGGGGTTTAGATGCAGTAGGTGTTTTAGATGATGGTAGTGATTCGGATATTTAGTAGGGGTTTAGATCTTAGTAGGTGTTTTTGTATAGTAGGGAGTTTAGGATTTAGTAGGGGTTTAGATGCTAGTAGGGGTTTTGGTATAGTAGGTTTAGATGATAGTAGGGGTTAGATGATAGTGGGGTTAGGGGATTTAGTAAGGGTTTAAAATCTTAAAGTAGGGAGTTTTGTTGTAGTACGGGTTTAGATGATAGTTCGGTTAGGAGATATAGTGGGATTTAGATGAAAGTAGGGGTTTAGGAGATTTAGTAGGGGTTTTAGATGCTAGTAGGGGTTTGGGAGATTTGTAGTGAGGTTTAGATGCTAGTAGGGGTTTTGGTATAGGAAGGGTTTAGATGATAGTAAGGGTTAGGAGATTTAGTAGGGGTTTAGATCTTTAGTAAAGGTTTATGAAGTTAGTAGGTTTAGATGCTAGTAGGGGTTAGATGATAGTAGAGTTTAGGTTATAGTAGGGGTTAGGGATTTAGTAAGAGGGTTAGATCTTTAGTAGGGGGTTTTGTTATAGTAGGGGTTTAGATAGTAGGGGTTTAGGAGATTTAGTAGGGTTTAGATGCTAGTAGGGTTTTTTGTATAGTAGTGGTTTAGATGATAGCGGGGTTGAGAGTTTAATGAGGGTATAGATCTTTAGTGAGGGTTTCTTTATAATGGGGTTTAGATGATAGTATGGGTTAGGCGTTTAGTAGGGGTTTAGATCTTTAGTAGGGATTTTGGTATAGTAGAGGTTTAGGGATTTAGTAGGGTATCATTTGCTAGTGGGGTTTTTTGTATAGTAGTGATTTAGATGATAGTGAGTTAGGGGATTTAATAGTGGTTTAGGGTTTAGGGTTTAGGGTTTAGGGTTTAGATCTTATTAGGGTTTGCTATAGTCGGGGGTTTAGATGATAGTAGGGGTTAGTGATTTAGTAGGGGTTTAGTTACTAGTAGGGATTTTGGGTATAGTAGGGGTTTAGGAGATTTAGTAGGTGTGTGAATCTTTGGCTAGGGAGTTTAGTTGATAGTAGGTGTTAGGAGTTTAGTAGGGGTTTAGATGCAGTAGGTGTTTAGATGATGGTAGTGATTAGGATATTTAGTAGGGGTTTGGATGCTAGTAAGGTGTTTTGGTATAGTAGGGGTTTTAGGAGATTTAGTAGGGTTTGAATCTTTAGTAGGGGTTTTGGTATAGTAGGGGTTTAGATGATAGTAGGGGTTTAGATGATAGTAGGGGTTTGGGGATTTAGTAAAGAAAGTTAGATGCTAGTAGAGGTTTTGTTGTAGTAGGGGTTTGGATGATAGTTCGGTGAGGAGATATAGTGAGTTTAGATGAAAGTAGGGGTTTAGGAGTTTAGTAGGGTTTAGATGCTAGGCTAGGGGTTTAGGAGATTTAGTAGGGGTTTAGATGCTAGTAGGGGTTTTGGTATAGGAAAGGTTTAGATGATAGTAAGGGTTAGGAGGTTAGTGAGGGTTTGGATCTTTTAGTAAGGGTGTGAGATTTAGTAGGGGTTTAGATGCTAGTAGAGTTTAGATGATAATTAGAGGTTTAGGTTATAGTAGGGTTAGGGGGATTTAGTAAGAGGTTTAGATGCTAGTAGGGGTTTTGTTATATTGGAGGTTTAGATGATGAGCGGGGTTTAGGAGTTTAGTAGGGGTTTAGATGCTAGTAGGGGTTTTTGTATAGTAGTAGTTTAGATAGTAGGGGTTAGAGGAGTTTAATAGAGTGTGAATCTTTAGTAGGGGTTTCTTTATAGTTAGGTTTAGATGATAGTGAGGGTTAGGAGTTAGTAGGGGTATAGATGCTGAGTAGGGATTTGGGTATAGTAGGTTAGGAGTTAGTAGGTTATTGTAGATGCTAGTAGGGGTTTAGTTTGATAGTAGGTGTTAGGAGGTTAGTAGGGGTTTAGATGGGAAGTAGTGTTTAGATGATGGTAGTGATTAGGATGTTAGTAGGGGTTTGAATCTTTAGTAGGTGTTTTGTATAATGGGGGTTTAGGAGATTTAGTAGGGGTTTAGATGCTAGTAGGGGTTTTTTGGTATAGTAGGGAGTTTAGATGATAGTAGGGGTTTAGATGATAGAAGTAGGGGTTAGGGATTTAGTAGGGGTTTAGATGCTAAATAGAGGTTTTGTTGTAATCTGAGTTTAGATGATAGTTCGGTTAGGAGATATAGTAGGGATTTAGATGAAAGTAGGGGTTGGAATTTAGTAGGGAGGTTTGAATCTTTGTAGTGAGGGTTTAGGAAGTTAGTAGGGGTTTAGATGCTAGTAAGGGTTTTGGTATAGGAAGGTTTAGATGATAGTGATAAGGGTTAGGAGATTTAGTAGGGTTTAGATGCTAGTAAGTTTATGAGATTTAGTAGGGGTTTTGAATGCTAGTAGGGGTTTAGATGATAGTAGAGGTTTAGGTTATAGTAGAGTTAGGAGTTTAGTGAGGTTTAGATCTTAGTAGGGGTTTTATTTATAGTAGGTTTAGATGATATTAGGGGTTTAGGAGATTTAGTAGGGGTTTAGATGCTAGTAGGGTTTGTATAGTAGTGGTTTTGATAAATGGAGGTTAGGGTTTAATAGGGGTATAGATCTTTAGTAGGGTTTTGCTATAATAGGGGTTTAGATAGTATGGGTTAGGCGGTTAGTAGGGAGGTTTAGATCTTTAGTAGGGATTTTGGTATAGTAGGGGTTTAGAGATTTGGTAGGGTATAGGTACTTTAGTAGGGGTTTTGTATAGTAGTGATTTAGATGATAGTAGAGGTTGGGGGATTTAATAGTGGTTTAGGGTTTAGGGTTTAGGGTTTAGGGTTTAGATCTGTGGGGTTTTGCTTTATAGTCGGGGTTTAGATGATAGTGGGGTTAGGCGATTTAGTAGGGGTTTAGATGCTAGTAGGGATTTTGGTATAGTAGGGGTTTAGGAGATTTAGTAGGTGTGTAGATGCTAGTAGGGGTTTAGTTGATGATAGGTGTTAGGAGATTTAGTAGGGGTTTTTAGATGCAAGTAGGTGTTTAGATGATGATAGTGATTAGGATGTTAGTAGGGGTTTTAGATGCTAGTAGGTGTTTTGGTATAGTAGGGTTTAGGAGAGATTTAGTAGGGGGGTTTGGATGCTAGTAGGGTTTTGGTATAGTAAGGGTTTAGATGATAGTAGGGGTTTAGATGATAGTAGAGTTTGGGGAGTTTAGTAAGGGTTTAGATGCTAGTAGGGGTTTTTTTTTTTTTTTTTTTTGTTGTAGTGGGTTTAGATGATAGTTCGGTAAGAGGAAGTATAGTGAGTTTAGATGAAAGTAGGGTTTAGGAGATTTAGTAGGGGTTTAGATGCGTTAGTAGGGGTTTAGGAGATTTAGTAGGGGAGTTTAGATGCTAGTAGGGGTTTGGTATGGAAGGGTTTAGATGATAATGAGTTAGGAGATTTAGTAGGGGTTTTTTGGATGCTAGTAAGGGTTTATGAAAGATTTAGTAGGGGTTTAGATGCTAGTAGGGGTTTAGATGATAGTAGAGTTTAGTTATAGTAGGGGGTTAGGGGGATTTAGTAAGAGGGTTTAGATGCTAGTAGGGGTTTTTGTTATAGTAGGGGTTTAGATGATAGTAGGGGGTTTTGGGGGATTTAGTAGGGTTTGAATCTTTGTGGGGTTTTGTATAGTAGTGGTTTAGATGATAGTAGGGGTTAGGGGTTGTAGGAATTATGAATCTTTAGTAGGGTTTCTAGCATAGTTGGGGTTTAGATGATAGTAGGGGTTGGGAGATTTAGTAGGGGTATAGATCTTTGTAGTAGGGATTTGGGTATAGTAGGGTTTAGGAGATTTAGTAGGTGTGCTTAGATGCTAGTAGGGTTTAGTTGATAGTAGGTGTTAGGAGGTTAGTGGAGGTTTAGATGCAGTAGGTGTTTAGATGATAGTAGTGATTAGGATATTTTAGTAGGGGTTTAGATGCTAGTGGTGTTTTTTTGTATAGTAGGGGTTTAGGAATTTATTAGGGTTTAGATGTGAGTAGGGTTTGGTATAGTAGGGGTTTAGATGATAGTAGGGGTTTAGATGATAGTAGGGGTTAGGGGATTTAGTAAGGGTTTAGATGCTAGTAGGGGTTTTTGTTGTAGTACGGGTTTTTAGATGATAGTTCAGGTTCTAGGAGATATAGTAGGGATTTAGATGAAATTAGGGGTTGGAGATTTAGTAGGGGTTTAGATGCTAGTAGGGGTTTAGGAAGTTAGTGGGGGGTTTAGATGCTAGTAGGGGTTTTGGTATAGGAAGGGTTTGAATGATAGTAAGGGTTAAGGAGATTTAGTAGGAGTTTAGATGCTAGTAAGGGTTTATGAAGAGATTTAGTAGGGAGTTTAGATGCTAGTAGGAGTTTAGATGATAGTAGAGTTTAGGTTATAGTAGGGTTAGGGAGTTTGTGGATTTAGATCTTTGAGTAGGGGTTTTTTTTTTATGGTAGGGGTTTAGATGATAGTAGGGTTTAGGAGATTTAGTGAGTTTTGAATCTCTTTAGTAGGGGTTTTGTATAGTGATGGTTTAGATGATAGCAGTTAGGGGATTTAATAGGGAGTATAGATGCTAGTAGGGTTTTGTATAGTTGGGGTTTGAATAGTGGGGTTAGGAGTTGAAGTAGGGGTGTGATGCTAGTAGGGGTTTGGTATAGTAGGGGGTTTAGGAGATTAGTAGGGGTTTGATGCTTGTAGTGGTTTGGTATAGTAGTGGTTTAGATGATAGTAGAGTTAGGGGATTTAATAAGGGTTTGTTGCTAAGTGATTTTGCTATAGTAGGGGTTTAGATGATAGTAGGGTTAGGAGAATTAGTAGGGAGTATAGGCTCTTTATTAGGGGTTTTTGTATAGTATGGGTTTAAGAGATTTAGTAGGAATTATAATTTCTTTAGTAGGTTCGGTTATTATAGGGGGTTTGGAGTTAGTAGGGGTTTTAGATGCTAGTAGGGGTTTTTGTATAGTAGGGTTTAGGGATTTAGTAGGGGTTTAGATCTTTGTGGGGTTTAGATTATAGTAGGGGTTTAGGAGATTTAGTAAGAGGTTTGAATCTTTAGTAGGTTTCTTTATAAGTAGGGGTTTTAGATGATAGTAGGGGGTTAAGAATATAGTAGGGTTTAGATGATAGTAGGGGTTTTTTTTTGTATAGTAGGGTTTGGAGATTTAGTAGGGGTTTAGATGCTAGTAGGGTTTAGGAGATTTAGTAGGGGTTTAGATCTTAGTAGGGGTTTTGGTATAGTAGGGGTTTAGATGATAGTAGGGTTAGGAGATTTAGTAGGGGTTTGAATCTTGTAGGGGTTTTGGTATAGTAGCGGTTTAGATGATAGTAGGGGTTAGGGATTTAATAGGGGTATAGATCTTTATTAGGGTTTTTCTTTATAGTTGGGGTTTAGATGATAGTAGGGGGTTAGGAGATTTAGTAGGTATAGATGCTAGTAAGGGTTTGGTATAGTAGGGTTTAGGAGATTTAGTAGGGGTTTGATGCTAGTAGTGGTTTGGTATAGTAGTGGTTTAGATGATAGTAGGGGTTAGGGGATTTGTAAGGAGTTTAGAGCTGTGAAGGGATTTTTGCTATAGTAGGGAGTTTAGATGATAGTAGGAGTTAGGAGATTTAGTACGGGTATAGGCTAGTAGGGGTTTTAGCCCCATGGCAGGCAGTATGGGTTTAGGAGAGATTTAGTAGGGGTATAGGCTGGCAGTAGGGGTTTCGAGTTATTATAGGGGTTTGGAGATTTCGTAGGAGTTTAGATGCTAGTAGGGGTTTTGTATAGTAGGGGTTTAGGAGATTTGGTAGGGGTTTCGATCTTTAGAAGAGGTTTTAGATGCTAGTGGGGTTTTTTGTATAGTAGGGAGTTTAGGAGGATTTTAGTAGGGGTTTGAATTAACTAGTAGAGGTTTGAATTATAGTAGGGGTTTGGGAAGTTAGTAAGAGGGTTTAGATCTTTAGTAGGGGTTTTGCTATAGTAGGGGTTTAGATTATACTAGGGTTAGGAGATATAGTAGAGGTTTAGATGATAGTGAGGTTTTTGTATAGTAGGGTTTAGGAGATTTAGAAGGGAGTTTAGATCTTAGTAGGGGTTTGATTTGGTAAGTTTAGATGCTAGTGAGGGTTTGGTATAGTAGGGGTTTTAGATGATAGTAGGGTTAGGAGATTCGGTAGGGGTTTTAGATGCTAGTAGGGGTTTGTGGTATAGTAGCAGTTTAGATGATAGTAGGGGTTAGGGGGATTTAATGGGGTTTATATGCTAGTAGGGTTTTTTTGCTATAATAGAAGTTTAGATGATAGTATGGGTTAGGCGATTTAGTAGGGGTTTAGATCTTTATTAGGGATTTTGGTATAGTAGGGGTTTAGGAGATTTAGTAGAGTTTGGATGCTGTGCAGAGGTTTAGTTGATAGTAGGGGTTAGGAAATTTAGTAGGGGTTTAGGTGCAAGTAGGGGTTTGAGATGATAGTAGTGGTTAGGATATTTAGTAGGGGGTTTAGATGCTAGTAGGTGTTTGGTATAGTAGGGGTTTAGGAGATTTAGTAGGGAGGTTTGAATCTTTAGTAGGAGTTTGGTATAGTAGGGGTTTAGATGATAGTAGGGGTTAGGGAGATTTAGTAAGGGTTAGATCTTTAGTAGGGGTTTTGTTATAGTAGGTTTTAGATGATAGTGGGGGTTGGGACATATAGTAGGGATTTAGATGAAAGTAGGGGTTTTGGTATAGTAGGGGTTTAGGAGTTTAGTAGGGTTTAGATCTGTGGGGTTTAGGGATTTAGTAGGGGTTTAGATGCTAGTAGGGTTTTGGTATAGTAAGGGTTTAGATGATAGTAGGGTTGGGAGATTGTAGAGTTTAGATGCTAGTAGGGTTTAGATGCTAGTAGGGGTTTAAGAGGATTTAGTATGGGTTTAGATGCTAGTAGGGAGTTTAGATGATAGTAGAGGTTTAGGTTATAGTAGGGTTGGGGGATTTAGTAGGTTTAGATGCTAGTAGGGATTTTTGTTATAGTAGGGGTTTAGATGATAGTAGGTGTTGGAGATATAGTAGGGATTTAGATGATGGTAGGGGTTTTGCTATAGTAGGGTTTAGATGATAGTAGGGTTTAAGATGATAGTAGGGGTTTGAATCTTGGTAGGGCTTTTGTATAGTAGGGGTTTAGGGCGTTTAGTAGGAGTTTAGATCTTTAGTAGGTGTTTGGGGCGATAGTAGGGAGTTCTGAGATTTAGTAGGGTTTAGATCACAGTAGGGGGTTTAGATGATAGTAGTTGTTAGGATGTTATTAGGGGTTTAGATGCTAGTAGGTGTTTTTGGTATAGTAGGGGTTTGGAGATTTAAGTAAGGGTTTAGATGCTAGTAGGGGTTTGGTATAGTAGGGGTTTAGATGATAGTAGGGGTTAGGGGATTTAGTAGGGACAAGATGCTAGTGAGGTTTAGATGCTAGTAGGGGTTTAGGAGATTTAGTAGGAGTTTAGATATAGTAGGGGTTTAGATGATAGTGAGGTTAGGGGGTTGGTAGGGTTTTAGATCTTTAGTAGGGGTTTTGCTATAGTATGGGTTTAGAAGATAGTAGGGGTTAGGAGATTTAGAAGGGGTTTAAATGCTAGTAGGGGTTTCGATTATACTTGAGGGGAGTTTGGGGATTTAGTAGGGTTTAGATGCTAGTAGGGGTTTGGTATAGTAGGGGGTTTACATGATAGTAATGGTTTGGAGATTTGAAGTAGAGGTTTAGATGCTAGTAGGGGTTTAGATGCTAGTAGGGTTTAAGAGATTTGAGCGGGGGGTTTAGATCTTTAGTAGAGTTTAGATGCTATTAGGGATTTTGTTATAGTAGGGGTTTAGATGATATGATGGTGGGGGGTTAGGAGATATAGTAGGGATTTAGATGATAGTAGGGGTTTGTATAGTATGGGTTAGATGATAGTAGGGTTTAGATGATAGTAGGGGTTTAGATGATAGTAGGGAGTTTAGATCTTCAGTAGGGGCTTTTGTATATTATGGGTTTAGGAGATTAGTGAGGGTTTTAGATGCTAGTAGGGGTTTGAGTTGATGAAGTAGGGTTCACGAGATTTAGTAGGGGTTGAATCTGATAGGGGTTTGAATGATAGTAGTTGTTAGGATATTTAGTAGGGGGTTTAGATGCTAGTAGGTGTTTTGGTATAAATGGGGTTTGGAGATTTAGTAGGGTTAGATGCTAGTAGGGGTTTGAGTATGGTAGGGAGGTTTAGATGATAGCAGGGGTTGAGGGATTTATTAGGGGTTGAATCTTGTAGAGTTTAGATGCTAGTAGGGGTTTAGGAGATTTAGTTAGGGTTTGAAGCGTAGTAGGGAATTGTAGATGATAGTAAGGGTTAGGGGATTTAGTAGGGGTTTTAGATGCAAGTAAGGGTTGTGCTATAGTATGGGTTTAGAAGATAGTAGAGAAATTCGGGAGATTAGAAGGGGTTTAAATGCTAGTAGGGTTTCGTTATATAGGGGTTTGGAATTTAGTAGGGGTTTTTAGATGCTAGTAGGGTTTTTGGTATAAATAGGGGTTTAGATGATGTCAGATTCTGAGATTTAGTAAGGAGTTTAGATACTAGTAGAGTTCTGATGCTAGTAGGGAGTTTAGGAGATTCTTGGGGTTTAGATGCTATTGGGGTTTAGGTGCTAGTAGGGGTTTTGCTATAGTATGGGTTTAGATGATAGTAGGGTTAGGAGTTTGAAGGGTTTAAATGCTAGTAGGGTTTAGAGTTATAGCAGGGGTTGGAGATTTAGTAGGGTTTAGATGCTTGAAGGGGTTTTGGTATAGTAGGGGTTTAGGAGATATAGTAGGGGTTTGAATGCTAGTAGGGGTTTTAGATGCTAGTATGGAATTTAGTATAGTAGTGATTTAGATGATAGTAGGGGTTGGGGATTTAATAGTGGTTTAGGGTTTTAGGGTTTAGGGTTTAGATGCTAGTAGGGTTTGCTATAGTCGAGGTTTTAGATGATAGTAGGGGTTAGGCGATTTAGTAGGGGTTTAGATGCTAGTATGGATTTTTAGTATAGTAAGGGGTTTAGGAGAGATTTAGTAGGTGTGTAGATTCTAGTAGAGGTTTAGTTGATAGTAGGTGTTAGGAGATTTAGTAGGGGTTTAGATGCAAGTAGGTGTTTAGATGATAGTAA

**Data S7** Example of DTRs with monomers elongation

*C. acuminatum*, accession 429-3, read 8544 (18185 bp)

TRF analysis:

Sequence: 429-3_8544

Parameters: 2 7 7 80 10 50 500

Length: 18185

| Indices | Period Size | Copy Number | Consensus Size | Percent Matches | Percent Indels | Score | A | C | G | T | Entropy (0-2) |
| --- | --- | --- | --- | --- | --- | --- | --- | --- | --- | --- | --- |
| 28--163 | 7 | 20.3 | 7 | 72 | 23 | 73 | 13 | 38 | 33 | 15 | 1.86 |
| 39--389 | 19 | 18.4 | 19 | 68 | 24 | 122 | 12 | 36 | 34 | 16 | 1.87 |
| 29--389 | 26 | 13.7 | 26 | 72 | 20 | 159 | 12 | 36 | 34 | 16 | 1.87 |
| 29--387 | 6 | 55.3 | 6 | 70 | 20 | 149 | 12 | 36 | 34 | 16 | 1.87 |
| 29--389 | 13 | 28.8 | 13 | 69 | 22 | 138 | 12 | 36 | 34 | 16 | 1.87 |
| 220--324 | 23 | 4.3 | 23 | 79 | 9 | 102 | 15 | 33 | 34 | 17 | 1.91 |
| 384--524 | 31 | 4.5 | 30 | 76 | 12 | 115 | 4 | 33 | 42 | 19 | 1.71 |
| 109--16869 | 6 | 2563.7 | 6 | 69 | 17 | 4367 | 9 | 35 | 38 | 15 | 1.80 |
| 439--17961 | 13 | 1319.3 | 13 | 69 | 18 | 4187 | 9 | 35 | 39 | 15 | 1.80 |
| 1531--1582 | 21 | 2.5 | 21 | 84 | 9 | 63 | 9 | 40 | 36 | 13 | 1.77 |
| 1850--3404 | 93 | 16.9 | 91 | 72 | 18 | 546 | 7 | 36 | 40 | 14 | 1.75 |
| 3269--3362 | 25 | 3.5 | 25 | 78 | 16 | 100 | 6 | 38 | 41 | 13 | 1.70 |
| 3538--3575 | 15 | 2.5 | 15 | 91 | 0 | 58 | 13 | 39 | 34 | 13 | 1.83 |
| 4363--4400 | 14 | 2.7 | 15 | 88 | 8 | 53 | 10 | 36 | 36 | 15 | 1.82 |
| 4989--5040 | 25 | 2.1 | 25 | 85 | 7 | 70 | 7 | 34 | 44 | 13 | 1.72 |
| 5083--5139 | 29 | 2.0 | 29 | 86 | 6 | 80 | 17 | 38 | 29 | 14 | 1.89 |
| 5103--5146 | 15 | 3.0 | 15 | 83 | 12 | 56 | 18 | 40 | 29 | 11 | 1.85 |
| 5223--6348 | 7 | 168.0 | 7 | 77 | 14 | 715 | 5 | 37 | 41 | 15 | 1.71 |
| 5528--5563 | 11 | 3.1 | 11 | 88 | 11 | 54 | 0 | 33 | 50 | 16 | 1.46 |
| 7847--7961 | 7 | 17.0 | 7 | 80 | 12 | 98 | 12 | 39 | 34 | 13 | 1.83 |
| 8504--8532 | 15 | 1.9 | 15 | 100 | 0 | 58 | 13 | 41 | 31 | 13 | 1.84 |
| 9255--9342 | 22 | 3.7 | 23 | 77 | 10 | 79 | 5 | 35 | 45 | 13 | 1.67 |
| Indices | Period Size | Copy Number | Consensus Size | Percent Matches | Percent Indels | Score | A | C | G | T | Entropy (0-2) |
| 10177--10210 | 15 | 2.3 | 15 | 94 | 0 | 59 | 14 | 41 | 35 | 8 | 1.77 |
| 11506--11564 | 22 | 2.8 | 20 | 83 | 16 | 75 | 13 | 35 | 35 | 15 | 1.87 |
| 11794--11838 | 16 | 3.0 | 16 | 90 | 9 | 69 | 0 | 37 | 44 | 17 | 1.49 |
| 12888--12926 | 16 | 2.5 | 16 | 87 | 4 | 53 | 10 | 41 | 35 | 12 | 1.77 |
| 13167--13224 | 21 | 2.7 | 21 | 84 | 7 | 64 | 15 | 41 | 32 | 10 | 1.81 |
| 13221--13264 | 11 | 4.2 | 11 | 81 | 18 | 58 | 0 | 31 | 50 | 18 | 1.47 |
| 13356--13594 | 28 | 8.6 | 28 | 68 | 22 | 109 | 7 | 37 | 40 | 14 | 1.75 |
| 13433--13532 | 22 | 4.7 | 21 | 77 | 15 | 89 | 6 | 40 | 41 | 13 | 1.68 |
| 13370--13531 | 41 | 3.7 | 44 | 76 | 19 | 146 | 6 | 38 | 41 | 13 | 1.71 |
| 16862--16905 | 14 | 3.1 | 14 | 83 | 9 | 54 | 13 | 43 | 34 | 9 | 1.76 |
| 16874--16983 | 56 | 1.9 | 59 | 89 | 9 | 174 | 18 | 39 | 33 | 9 | 1.82 |
| 16995--17030 | 15 | 2.5 | 15 | 95 | 4 | 65 | 19 | 30 | 30 | 19 | 1.96 |
| 17024--17151 | 6 | 20.2 | 6 | 71 | 20 | 84 | 6 | 32 | 44 | 17 | 1.73 |
| 16994--17084 | 41 | 2.2 | 41 | 84 | 5 | 112 | 13 | 31 | 36 | 18 | 1.89 |
| 17244--17340 | 20 | 4.3 | 25 | 86 | 12 | 108 | 6 | 40 | 40 | 13 | 1.69 |
| 17261--17370 | 34 | 3.1 | 35 | 81 | 17 | 99 | 4 | 35 | 45 | 14 | 1.65 |
| 17293--17369 | 7 | 11.7 | 7 | 81 | 13 | 67 | 1 | 33 | 49 | 15 | 1.53 |
| 17296--17604 | 6 | 48.3 | 6 | 71 | 21 | 136 | 6 | 34 | 43 | 15 | 1.72 |
| 17348--17390 | 5 | 8.2 | 5 | 89 | 5 | 59 | 0 | 27 | 53 | 18 | 1.45 |
| 17458--17512 | 21 | 2.5 | 22 | 77 | 16 | 53 | 5 | 40 | 38 | 16 | 1.72 |
| 17477--17604 | 40 | 3.2 | 38 | 80 | 13 | 127 | 7 | 32 | 43 | 16 | 1.75 |
| 17612--17636 | 8 | 3.1 | 8 | 100 | 0 | 50 | 12 | 40 | 36 | 12 | 1.79 |
| Indices | Period Size | Copy Number | Consensus Size | Percent Matches | Percent Indels | Score | A | C | G | T | Entropy (0-2) |
| 17653--17698 | 6 | 7.2 | 6 | 86 | 13 | 65 | 2 | 34 | 47 | 15 | 1.57 |
| 17788--17831 | 14 | 2.9 | 15 | 86 | 10 | 63 | 15 | 43 | 29 | 11 | 1.82 |
| 17828--17933 | 6 | 16.8 | 6 | 85 | 11 | 142 | 0 | 33 | 48 | 16 | 1.53 |
| 17992--18021 | 13 | 2.2 | 14 | 94 | 5 | 53 | 6 | 36 | 40 | 16 | 1.75 |
| 18048--18077 | 13 | 2.3 | 13 | 94 | 0 | 51 | 3 | 40 | 43 | 13 | 1.60 |
| 17916--18100 | 73 | 2.5 | 72 | 76 | 15 | 184 | 11 | 38 | 36 | 12 | 1.81 |
| 18121--18169 | 25 | 2.0 | 25 | 95 | 0 | 89 | 10 | 40 | 38 | 10 | 1.73 |
| 18048--18177 | 19 | 6.7 | 18 | 70 | 15 | 55 | 12 | 38 | 36 | 12 | 1.80 |

Consensus pattern (6 bp):

CCTGGG

Consensus pattern (7 bp):

CCCTGGG

Consensus pattern (7 bp):

CCCTAGG

49 telomere motifs-based different consensus monomers totally.

Sequence:

>429-3_read_5413

GGTGTGCTGGTTCGGTTGCATTGTTGTCCCTAGGCCTGTAGGCCCTAGGCCTGAGCCTAGGCCCTAGGCCCTAGGCCTAGGCCTAGGCCCTGAGCCCTGGAAGCCCTAGGCCTGGGCCTAGGCCTGAGCCCTGGGCCTGGGCCCTGAGCCTAGGCCCTAGGCCTGGGCCTGGGTACCCCAGGCCTGGGCCACTGAGCCTGGCCTGGGCCTCGCCCTGGAAGGCCTAGGCCCTAGGCCTAGGCCTAAGGCCCTAGGTTTGGCCTAGGCCTAGGCCTAGGCCAGGCCTAGGCCTGGGCCTAAGGCCTAGGCCCTGGGCCTAGGCCTGGTACTGAGCCCTGGGCCTAGGCCCTAGGCCCTGAGCCTAGGCCTGGGCCTGGGCCCTAGGCCCTGAGCCTGGGCCTGTTTCAGGCCTGGGTACAGGCCTGGGCCTGTTTTTCAGGCCTGGGCCTGGGCCTGGGCCTGGGCCTGCAGGCCCTGGGCCCTGGGCCTGGGCCTGGGCCTGGGCCTGGGCCCTGGGCCTGGGCTGTAAACCCCTGAGCCAGGCCTGGGCCTGGGCCTGGGCCTGAATGCTGGGCACCTGGGCTGGGCCCTGGGCCACACAGGCCTGGGCCACAGGCCTGGGCCCTGGGCCTGGGCCTGGGCCTGGGCCCTGGCTGGCCTGGGCCCTGGGCCTGGGCCCTCGGGGCCTGGGCCTGGGCCTAAGCCTGGGCTAGGCCTGGAGCCACAGGCCTGGGCCTAGGCCTGGGCCTAGGCCCTGGGCCCTGGGCCTGGGCCCTGGGCCTGGGCCTGGGCCTAGGCCTGAGCCCTGGCTTCAGGCCCTGGAGAAGCCCTGTTTCAGGCCTGGGCCTGGGCCTGGGCCTGGGCCTGGGCCTGGGCCTGGGCCTGGGCCTGGGCCTGGTGCTGAGCCTGGGCCTGGGCCCTGGGCCTGGGCCTGGGCCCTGGGCCTGGGCCACCAGGCCTGGGCCCACCTGAGCCCAAACCCAAACCACAAGCCCTGGGCTGGGCCTGGTGCAGGCTGGGCCTGGGCCTGGGCCAGGCCTGGGCCTGGGCCTGGGCCCTGGCCCTGGGCCTGGGCCTGGGCCTGGGGCCCTGGGCCCTGGGCCACGGCCTGGGCCTGGGCCTGGGCCTGGGCCTGGGCCTGGGCCTGGTTTGGGCCTGGGCCTGGGCCTGGGCCTGGGCCCTGGGCCTGGGCCACTGAGCCTGGGCCCTGGGCCCTGAGCCCTGGGCCTAGGCCTGGGCCTGGGCCTGGGCCTAGGCCTGGGCCTGGGCCCTGGGCCTGGAGCCACTAAACCTGCCAAACCTGTAAGCCTAAACTACTAGGCCTGGGCCTGAGCCTAGGCCCTAGGCCTAGGCCCTGGGCCTGAGCCTGGGCCTAGGCCTGGGCCTGAGCCCTGGGCCTGGGCCTGGGCCCTGAGCCTGGGCCACCCAAACCTGGGCCTGAGCCTAGGCCTAGGCCTGGGCCTGAGCCTAGGCCTGGGCCCTGAGCCTGGCCTCCAGGCCCTAGGCCTAGGCCTGGGCTGCTGTTTCAGAGCCCTGAGCCACTGAGCCTAGGCCCTGAGGCCCTGGGCCTGGGCCCTGGGCCTAGGCCCTGGAGGCCACTGAGCCCTGGGCCTGGAATACTGGGCCCTGGGCCTGGGCCCTGGGCCTGGGCCACAGGCCTGGGCCCTGGGCCCTGGGCCTGGGCCCTGGGCCTCTGGGCCTGGGCCCTGGGCCCCAGGCCTGGGCCTAGGCCACTGGGCCGCCACAAGCCTGGGCCCTGGGCCTGGGCCTGGGCCTCAGGCCTGAGCCACTGGGCCTAGATACTGGGCCTGGGCCACAGGCCTGAGCCTGAGCCTAGGCCTGGGCCACAGGCCTGGGCCTGGGCCTGAGGCCTAGGCCCTAGGCCACAGGCCTGGGCCTGGGCCACAGGCCCTGGGCCTGGGCCTGGGCCACTGGCCTGGGCCTGGGTACAGGCCTGGCCTGGGCCCTGGGCCTGGGCCTGGGCCTGGGCCTGGGCCTGGGCCACAGGCCCTGGGCCTGGGCCTGGGCCCTGGGCCTGGTGCCTGGGCCTGGGCCTGGGCCTGAGGCCCTGGGCCTGGGCCTGGGCCTGGGCCTGGGCCTGGGCCTGGGCCACTGAGGCCCTGGGCCTGGGCCTGGGCCCTGGGCCTGGGCCTGGGCCCTGGGCCCTCGGCCCTGGGCCTGGGGCCTGGGCCCTGGGCCTGGGCACTGAGCCTGGGCCCTGAGCCCTGGGCCCACTGAGCCACCTAGGCCTACTGGCCTAAACCAGGCCTAGGCCACTGGGCCCTAAGGCCTAGGCCACTGGGCCACTGGCCTGGGCCTGGGCCTGGTACAGGCCCTAGGCCCCTAGCCTCAGGCCCTGGGCCTGGGCCCTGGGCCCTGGGCCCTGGCCACAGGCCTGGGCCACTGGGCCTGGGCTACTGAGGCACAGGCCCTGGGCCACTGAGCCTGGGCCACTGAGCCTAGGCCACTGGGCTGGGCCCACTAGGCTGGGCCTGGGCCTGGGCCTGGCCCACTCGTGCTGGGCCTGGGCCTGGGCCTGGGCCCTGGGCCCTGGGCCCTGGGCCTATTTCAGGCCTGGGCCTGGGCCCTGGGCCTAGGCCCTGGGCCCTGGGCCACGCAAGCCTAAGCCTGGCCTAGGCCCTGGGCCTGGGCCTGGGCCTGGGCCTGGGCCTGGGCCTGGGGCCTGGGCCTGGGGCCTGGGCCTGGGCCCTGGAGGCCCTGGGCCTGGGCCCTGGGCCCTGGGCCCTGGGCTGGGCCTGGGCCTGGGCCTGGGCCTGGGCCTGGGCCTGGGCCTAGGCCCTGGGCCTGGGCCTGGGCTGGGCCTGAGCCTGGGCCTGGGCCTGGGCCCTGCCACTGGGCCCATTTTCAGGCCCTGGGCCCTGGAGCCTGAGGCCTGGGCCTAGGCCCACAAGGCCACAGGCCTAGGCCCTCAGGCCCTGAAGCCTGGGCCTGAGCCTGGGCCCTGGGCCTAGGCCTGGGCCTAGGCCTAGGCCTAGGCCTAGGCCTGGGCCCTAGGCCCTGGGCCTGGGCCCTGGGCCACGACCCTGGGCCTGGGCCTGGGCCCTGGCCCCCTCAGGCCTGGAGGCCTAGGCCTAGGCCTGGGCCTGGGCCTGGGCCTGGGCCACTGGGCCACAGGCCTAGGCCTGGGCCACAGGCCTAGGCCCTGGGCCTGGGCCTGGGCCTGGGCTGGGCCTGGGCCTGGGCCTGGGCCTGGGGCCCTGGGCCTGGGCCTGGGCCCTGAGCCACACAGGCCCTGAGTACTGAGCCACTGGGCCTGGGCCCTGCAGGCCTGGGCCTAGGCCACTGGGCCACCAGGCCCTGGGCCTGGGCCTGGGCCTAGGCCCTGGGCCTGGGCCTGGGCCACTGGGCCTAAGCCCTCAGGCCCTAGGCCTGGGCCCTGGGCCCTGGGCTGGAGGCCTGGTGCTGAGCCCTGGGCCTGGCTTTCAAGCCAGGCTCATTGGCTCATTGGGCCAAAGCCTAAACTAAACTAGTAGATCTCATTGTTAGATACTTTAGGCCTAGGCCCTGGGCCTCAGGCCTGAGCCACTGAGGCCTGGGCCACTGAGCCCTGGGCCTGGCCTAGGCCTGGGCCTGGGCCACTGGGCCTGGGTACTGGAGGCCCTGGGCCCCCAGGCTCTGCTGGGCCACTGGCCACAGGCCCTGGGCCTGGGCCCTGGGCCACTGAGCCTGGGCCCTGGGCCTGGGCCTGAATACTGGGCCTGGCCTCGGTGCTGGGCCTGGGCAAACCCTGGGCCTGGGCCCTGGGCCCTGGGCCCTAGGCCCTAGGCCTGGGCCTGAGGCCTGGGCCTGGAGCCTGAGCCTGGGCCTGGGCCACCTGAGGCCCTGGGCCCACTGGACACTGGGCCTGGTGCACAGGCCACTGGGCCTGGGCCTGGGCCCTGAGCCCTGGGCCCTAGGCCTAGGCCCTGGGCCCTCGACCCTAGGCCTGGGCCTGAGCCCTGGGCCCTCAGGCTGGGGCCTGGGCATTTTCTGAGCCCTAGGCCACAGGCCCTGGGCCCTGGGCCTGGGCCTCAGGCCTGGGCCTGGGCCTGGGCCTGGGCCACTGGGCCTGGAGCTGTAAGACCACTGGGCCACTGGAGCCTCAAGGCCTGACCTGGAAGCCTGGCCCACTGAGCCCTGGGCTGCAGGCCCTCAGGCCACTGGCCTGGGCCTGGGCCTGGGCCCTGGGCACAGGCCTGGGCCTAGGCCCCTGGGGCCTGGGCCTGGGCCCTGGGCCTGGGCCTGGGCCTGGGCCTGGGCCCTGGGCCTGGGCCTGGGCCCTGGGGCCTGGGCCCTGGGCCGGCTGGGCCTGGGCCTCGGCTGCAAGCCTAGGCCCTGTTTGGGCCTGGGCCCTGAGCCTGGGCCCTGGGTACTGAGCCCTGGGCCACTGAGCCTGGGCCTGCGTGCACAGGCCTGGGCCACAAACCACAGGCCCTGGGCCCTGGGCCTAGGCCCACAGGCCCTGGGCCCTGGGCCCTGGGCCCTGGGCCTGGGCCCTGAAGCCCTAAGCCTAAGGCCTAGGCCTCAGGCCCTGGGCCTGGCTGGGTACTCAGGCCTGGGCCTGAGCCTGGGCCCTGGGCCTAGGCCTGGGCCTGAGCCCTGGGCCTGGGCCTGGGCCTGGGCCCTGGGCCTGGGCCTGGGCCTGAGCCTGGGCCTGGGCCTGGGCCTGGGCCCTGGGCCTGGGCCCTGGGCCACAGGCCTGGGCCTGGGCCTAGGCCACGGGCCACTGAGCCTGGGCCTGGGCCCTGGGCCCTGGGCCTGGGCCCTGGGCCTGGGCCTGGGCCTGGGCCCTGGGCCTGGGCCCTGGGCCTGGGCCCTGTTTCGCCTAGGCCCTAGGCCCACTAAACCCAAGTGCCTGGGCCCTGGGCCTGGGCCTGGGCCCTGGGTACAGGCCACTGGGCCTGGGCCTGGGCCTGGGCCCTGGGCCCTGGGCCCTGGGCGCCTAAACCTGGGCCTACAAACCACAAGCTAGGCCTGAGCCTAAACTAGGCCTGGGCCACAGGCCTAGGCCTGGGCCTGGGCCTGGGCCCTAGGCCTGGGGCCACAAACCTAGGCACCTGAAACCTCGGCCTGGCCTAATTTCAGGCCCTGAGCCTGGGCTTACTGAGCCACAGGCCCTGAGCCCTAGGCCACTGAGCCCTGGGCCTGGAAGCCTAGGCCCTGGAGCCACTGAGCCATGCAAACACAGGCCTAGGCCCACTAGTTGCTGAGCCACTGAGCCACTGGGCCTGGGCCTGGGCCTGGGCCTGGGCCTGGGCCTGGGCCTGGGCCTGGGCCACTGGGCCCTGGGCCCTGGGCCCTGGGCCACTGGGCCTGAGCCTGGAGCCTGGGCCCACTGAGCCCTGTTCCCTATGACCTCAAGCCCTGGGCCCTGGGCCCTGAGCTAAATCTCCTGGGCTGCTCTCAGGCCTGGGCCTCAAGGCCCTGGGCCTGGGCCTAGGCCTGGGCCTGGGCCTGGGCCTGGGCCCTGGGCCTGGGCCTGGGCCTGGGCCTGGGCCTAGGCCCACTGGGCCCTAGGCCCTGGGCCCTGGGCCTGGGCTGGGCCTGGGCCTGGGCCCTGGGCCGCCAGGCCTGGCCACTGGGCTACTGGGGCCCTAGGCCTGGGCCTGGGCCTGGGCCTAGGCCTGGGTGCAGGCCCTGGAGGCCCAGGCCTCTGGGCCACTGGGCCCTGAGCCCACAGGCCCTGGGCCCTGGGCCTGGGCCCTGGGCCCTGGGCCCTGGGCCCTGGGCCTGGGCCCTGGGCCCTGGCCTGGGCTGGGCCCTGGGCCCCTGGGCCTAAAGATACTGGGCCTAGGCCCTGGGGCCCTGCAGGCCCTGGGCCCTGGGCCCTGGGCCTATTTCCTGGGCCCTAGGCCCTGGGCCTAGGCCCTGGGCCCTGGGCCTGGCCTGGGCCTGGCCTGGGCCTGGGCCTGGGCCCTGGGCCTAGGCCTGAGCCCTGGGCCGCAGGCCTGGGCCCTGGGCCTGAGCCCTGGGCCTGGGCCTGGGCCCTGGGCCTGAGCCCTGGGCCCTGGGCCTGGGGCCTGGGCCTGAGCCCTGGGCCCTGGGCCACAGGCCCTGGGCCCTGGGCCCTGGGCCTGGGCCTGGAGCCTGAGGCCCTGAGCCCTGGGCCCTGGGCCTGGGCCTGGGCCACTGGGCCTGGGCCTAGGCCCTGGGCCCTGGAGGCCTGGGCCCTGGGCCTGGGCCTGGGCCCTGGGCCCTGGGCCCTGGGCCTAGGCCTGGGCCCTGGGCCTGGGCCTGGGCCCTGGGCCTGGGCCACAGGCCTGGGCCTGGGCCTGGGCCTGGGCCCTGGGCCTGAGTATGGCCTGAGCCCTGGGCCTGAGCCCTGAGCCTGGGCCTGGGCCCTGGGCCTAAACCCCCAAATGCAAGCCAAGCCTGGAGCCTGAAGCCTATGGTGGCCTAGGCTGAGGCCTGGGCCTAAACCACAAACCCTATGGCCTAAGGCCTGCCCCAGGCCTAGGCCTGGGCCTGGGCCTAGGCCTGGGCCGCCTAAACTAAACTAAACCTAGACCATAAACCTGGGCTCACAAACTAGTACAAACTCTAGGCCTGGGCTACCCTGGGCCTAGGCCTGGGCCTGGGCCTGGGCCTGGGCCTGGGCCTACCAGGCCTGGGCCTGGGCCTGGGCCTGGAGGCCTGAGCCCTGGGCCCTGGGCCCTGGGCCCTGGGCTGGGCCTGGGCCTGGGCCTGGGGCCTGAGGCCTGGGCCTAGGCCTGGGCCTAGGCCCTGGGCCTAGGCCCTGGGCCTGGAGTACTGAAGCCCTAAACCTGAGCCTGGAAGCTGGGCCACAAACCTAGGCCTGGGCCCTGGGCCCTGAACCACGAAGCCTGACCTATTTGTTCTGGGCCACTGAAGCACCTGGGCCTAGGCCTGAGCCTGGGCCCTAAACTCAGGCCCTGAGCCTGAGCCTGAGGCCTGGGCCGCAGGCCTGAGCCCTAGGCCACTGAGCCTAGGCCTGAGCCTGGGCCCTGGGCTGGGCTAGGCCTGGGCCCTGGGCCTGGGCCCTGGGCCTGGTACACAGGCCTGGGCCTGGGCCCTGGGCCTGGGCCTGGGCCTGGGCCCTGGGCCCTGGGCCTGGGCCCTGGGCCTGGGCCTGAGGCCTGGGCCTGGGCCTGGGCCTGGGCCTGAGCCTGGGCTGGCTCAGGCCCTACGGCCACAAAACCCTAAACCTAGGCCTGGGCCTAGGCCTACATTCTGTTTTCAGGCCCACTGAGCCTAGGCTGAAACTGACTAAACCTGAAGCCTAGGCCTAGGCCCTAGGCCTAGGCCCTAGGCTCGGCCTAGGCCTGAGGCCTAGGCCTAGGCCTAGGGGCCTAGGCTTAAACTAAACCCTGGACCTGAGCCTAGGCCTGAGCCTAGGCCTGAGCCTGAGCCCTAGGCCCCTGGGGCCTGGGCCTAGATACTGAGGCCTGAATACTGGGCCCTGAGCCTAGGCCTAGGCTTTAGACTTAAACCACAAACCACAGGCTAGGCCTAGGCCTAGGCCCTGGGCCTGGGCCTGGGCCCTGGGCCTAGGCCTGGGCCTGGGCTACTGGGCCCACTGGGCCTGGGCCACTGAGCCTGAGCCTGGGGCCTGGGCCTGGGCCTGGGGCCTGGGCCTGGGCCTGGGCCTGGGCCCTGGGCCTGGGCCTGGGCCCTGGGCCCTGGGCCCTCAGGCCTGGGCCCTGGGCCTGGGCCTGGGCCACAGGCCCTGGGCCCTGGGCCTGCAGGCCTGGGCCTGGGCCTGGGCCTGGGCCTGGGCCTGGGCCTGGCTGGGCCTGGGCCTGGGCCTGGGCCCTGGGCCACAGGCCCTGGGCCTGGGCCACTGAGCCCTGGGCCTGGGCCTGGGCCCTGAAGCCCTGAGCCTAAACTAGGCACTGGGCCCTAGGCCTAGGCCTAGGCCCTAGGCCCTAGGCGCCTAGGCCATAGGCCACAGGCCTGGGCCCTGGGCCCTCGGCCTGGGCCTAGGCCCTAGGCCCTGGGCCTGAGCCACTGAGCCTGGGCCTGAGCCCTAGGCCTGGGCCTAGGCCTGAGCCTGGGCCTAGGCCTAAGCCAGGCCTGGGGCTATTTTTAAACCTGAGCCCACAGGCCTGGGCCTGGGCTAAACCTGGTGGGGCCTGGGCCTAGGCCTAGGCCCTGGGCCTAGGCCTAGGCCTAGGCCCTAGGCCCTAGGCCTGAGCCCTAAGGCCCTGGGCACAGGCCTGAGCCCTGGGCCTGGGCCACTGAGCCTGGGCCTGGGCCTGGGCCCTGGGCCCTAGGCCCTGGGCCTGGGCCTGGGCCCTGGGCCTGAGCCTAGACCACAGGCCTAGGCCTGGGCCTGAGCCTGAGGCCTGAGCCTGGAGGCCCTGGGCCTCAGAGCCTAGGCCTGGGCTGGAGCCTGGGCCTGGGCCTGGGCCTTTGCCTGGGCCCTAGGCTCCTGAGCCACAGGCCTGAGCCTGGGCCTGAGCCCTGGGCCTAGGCCTGGGCCACTGGCTCAGGCCCTGGGCCCTGGGCCTGGGCCTGGGCCTACAGCCTGGCTGGGGCCACTGAGCCCTAGGCCCTGGAGCCCTAGGCCCTAGGCCTGGGCCTAGGCCCCGAGGCCTGGGCCTGAGCCTGAGGCCTGGGCCTGGGCCTAGGCCTGGGCCTGGGCCTGGGCCTACAAACCCTGGGCCTGGGCCTAGGCCTGGGCCCTGGGCCTCGCCTGGGCCACAGGCACTGAGCCTGGGCCTGGGCCTGGGCCTGGGCCCTGGGTACCTGAGCCCTCGGCCTCGACACTGGGCCTGGAAGCCTGAGCCCTGAGCCCTGGGCCTAGGCCTGGGCCTAGGCCTAGGCCTGGGCCTGAGGCCTAGGCCCTGGGGCCCTAGGCCTGAGCCTGGGCCCTAGGCCTAGGCCCTGGGCCCTGGGCCTAGGCCTAGGCCTAGGCCACAGGCCCTGGAAGCCTAGGCCCTAGGCCTAGGCCGGGCCTGGGCCTGGGCCCTGGGGCCTAGGCTGCAAACCTGACCTGGGCCTGGCCACTATTTTAGGCCACCAGGCCTGGGCCTCAGGCCCTGGGCCCTGGGCCACTAGGCCTAGGCCTAGGCCTAGGCCTGGGGCCTGGGCCTGGGCCTGAGCCCTGGGCCCTCAGGCCTGGGCCTGGGGCCTGGGCCACGAATACAGGCCTGAGCCTGGGCCTGGGCCACAGGCCACTGGCCTCGGCCTGGGCCTCAGGCCTGGGCCTGGGCCTGGGCCTGGGCCCTAAACCTGGGCCTGGGCCTGGGCCTGGGCCTGGGCCTGGGCTGGGCCCTGAGCCCTGGGCCTGCGGCCTGGGCCTGGGCCACCGGAGGCCTGGGCCTGGGCCGCAGGCAGGCCCTGGGCCCTGGGTTTCAGGCCTGGGCCTGGGCCTGGGCCTGGGCCTGAGCTGGGCCTGGGCCCTGGGCCCTGGGCCCTGAGCCTGGGCCCTGGGCTGGCTGGCCTGAGCCTGGGCCTGGGCCTGGGCCTGGGCCTGGCCTCGCTCAGGCATAGGCCTGGGGCCCTGGGCCTGGGCCTGGGCCCTGAGCCTGGGCCTGGGCCACAGGCCACAGGCCTAGGCCCTGGGCCTAGGCCTGGGCCTGGGCCTAGGCCCTGGGCCTGGCTGTTTTCTGGCTGACCCTGGGCACCTAGGCCTGGAGCCTGGGCCTGGCCTGGGCCTGGGCCAGCAGGCCTGGGCCTGGGCCCTGGCACATAGGCCTGGGCCCGCAAACCTGGGCCCTGGGCCTAGGCCTAAGCAGAAGCCTAAATTTCAAGCTTCCTAGGCCCTAGGCCTGGGCCTGGGCCTGGGCCCTGGAAGCTAAACCCCTAGCCTGAAACAAGGCCTAAGATACAGGCCACTGAGCCCTGGGCCCTGGGCCCTGGGCCTGGGCATACTGTTGCACTGGGCCTGAGCCTGGGCCCTGGGCCTGGGCCTGGTGCAGGCCTGGGCCCTGGGCCTGGGCCTGGGCCCTGAGCCTGGGCCACAGCACATGCCTGGGCCCTGGGCCTGGGCCTGGGCCTGGGCCTGGGCCTGGGCCCTGGGCCTGGGCCCTGGGCCTGGCTAAACCTGTGGGCTCCCTGGGCCTGGGCTGCTGAGCCCTCAGGCCTGGGCCTGGGCCCTGGGCGCTGGGCGGCTCAGCCTCGTGCCCTGTGTTTCAAACCCTGGGCCCTGGGCCCTGGGCCCTAGGCCCTGGGCCACTGGCGTGGGCCTGGGCCCTGGGCCTGGCTGGGCTGGGCCTGGGCCACTCAGGCCTGGGCCACACAGGCCACAGGCCTGGGCCTGGGCCCTGGGCCTGGGCCTGGGCCTGGGCCCTGGGCCTGGGCCTGGGCCTGGGCCACCTGAGCCTGGGCCTGAGCCACTGGGCACTGGGCCACAGGCCCTGGGCCTGACCCTGGCGCCTGGCCCTGGGCCTGGGCCTGGGCCTGGGCCTGGGCCACAGGCCTCGGGCCCTGGGCCTGGGCCTGGGCCTGGGCCTGGGCCCTGGGCCTGGGCCTGGGCCTGGGCCTGGGCCTGGGCCCTGGGCCTCAGGCCTGGGCCTGGGCCTGGGCCTCAGGCCACAGGCCTGGGCGCTGGAGCCCTGGGCCTGGGCCTGGGCCTGGGCCTCTGAGGCCTGGGCCTGGGCCCTGGAGCCCTGAGGCCTGGGCCACAGGCGCCTGGGCCCTGGGCCTGGGCCTGGGCGCAGGCCTGGTGCAGCCTCGACACTCAAACCACAGGCCTGAGCCTAAACCTGGGCCCTGGGCCCTGGGCCTGGGCTGGGCCCTGGGCCTGAGCCCTGGGCCTGGCCTGGGCTTTCAGGCCTGGGGCCCCCAAACCTGGGCCTGAGCCTGAGCCCTGGGCCTGGGCTGAGCTGCACAGGCCTGGGCCACTGGGCCACAGGCCTGGGCCTGGGCCTGGGCCTGGGCCTAGGCCCTAGGCCTAGGCCTGGGCCTGGAGCCTGGCCCACTGGGCCTAGGCCCTGGGCCTGAGCCTGGGCCCTGGGCCCTGAGCCACTGGGCTGCCTAAACCTGGAAGCCTAGGCCTAGGCCCTGAGCCACAAGGCCTGAGCCTGGGCCACAGGCTGGGCTGGGCCTGGGCCACCTCGGCCTGGGCCTGGGCCTGGGCCTGGGCCTGGGCCTGGGCCTGGGCCTGGGCCTGGGCCTGGGCCCTGGGCCTGGGCCCTGGGCCTGAGCCCTAGGCCTGGGCCTGAGCCCTGGGCCTGGGCCCAGCACTGGGCCCTGGGCCTGAGCCACAGGCCCACTGAGCCCTGGGCTGGGCCACAGGCCCTGGGCCACTGGGCCTAGTGGGCCTGGGCCACTGGGCCCTGGGCCCTGAGGCCCTGGGCCCTGGGCCTGGCCCTGGGCCCTAGGCCTGGGCCTGGGCCTGGGCCTGGGCCACTGAGCCACTGAGCCCACAGGCCCACCTCGGCCTGGGCCTGGGGCCTGGGCCTGAGCCCTAGGCCTGGGCCCTGGCCTGGGCTTTAAATTACTGGAGAAGCCTCAGGCCTAGGCCTAGGCCCTAGGCTATGACCCTAGGCCCACAGGCCTAGGCCCTAGGTAGCTAGGCCTAGGCCTAGATACTGAGCCCTGGGCCACTGAGCCTGGGCTGCAGGCCACTGAGCCCTGGGTACTGGGCCACTGAGCCCCACTGGAAGCCTGAGCCTGAGCCACTGAGGCCTAAGGCCTCTGAGCCACTGGGCCTAGGCCTGGGCCCTAGGCCCTGGGCCTATTGCTGAGCCTAGACTGACCCACCACAGGCCTGGGCGACCTGGGCCTGGCCTCAGGCCTGGGCCTGGGCCCTGGGCGGCTGCAGGCCTGGGCTGGCCTGGCCTCAGGCCCTGGGCCTGGGCCTGGGCCACAGGCCTGGGCCTGGGCCTGGGCCTGGGCCCTGGGCCCTGGGCCTGCTGGGCCCTGGGCCTGGGCCCTGCGGGCCACAGGCCTGGGCCTGGGCCTGAGCCCTGGGCCCTGGGCCTGGGCCTGAGCCTGGGCCACAGGCCTGGGCCTCGGCCTGGGCCTACCCAGGCCCTGGGCCCTAGGCCCTGGCCGGAGCCTAGGCCCTAGACCTGGGCCTGGAGAAGCTATTTCTTCATTTCAGGCCTGGGCCTGGGCCACAGGCCTGAGCCTGGGCCTGGGCCTGGGCCACTGGGCCTGGTACAGGCCTAGGCCTGGGCCTGGGCCTGGGCCCTGGGCCTGGGCCTGGGCCTGGGCCTGGGCCCTGAGCCTGGGCCTGGGCCTGGGCCCTGGGCCCTGGGCCCTGGAGGCCCTGGGCCTAGGCCTGGGCCCTAGGCCCTGGGCCTAGGCCCTGGGCCTGGGCCGCAGGCCTGGGCCTGGGCCTGGGCCTATGGGCCCACTAGGCCACTGGGCCCTGGGCCCTGGGCCACAGGCCACTGGGCCTAGGCCTAGGCCACTGAGCCCACTGGCCTCAGGCCCTGGGCCCTGGGCCCTGGGCCTGGGCCTGGGCCTGGGCCCTGGGCCTGGGCCACAGGCCTGGTACTCAGGCCTGGGCCTAGGCCACTGGGCCCTGGGCCTGGGCCTGGGCCTAGGCCTGGGCCCTGAGCCTGGGCCTGGGCCCTGGGCCACTGGGCCCTGGAGCCACGGGACCGGAGCCTGGGCCCTGGGCCTGGCCGCAGGCCACTGGCCACTACAGGCCAGGCCCTGAGCCCTGGGCCCTGGGCTACTGGTACTGAGTACTGAGCCACTGAATACTGAGCCTAGGCCTAGGCCTGGGCCTGGGCCTGAGCCACTGCAGGCCTGGGCCACTGGGCCCTGGGCCACTGGGCCCTGGGCCTGGGCCTGGGCTGGGCCTGGAAGCCTGGGCCTGGGCCTGGGCCCTGGGCCTGGGCCTGGGCCTGAGTACAGGCCTCAGGCCTGGGCCTGGGGCCTGGGCCCTGGCCCTGGGCCCTGGGCCCTGGGCCTGGGCCTGGGCCTGGGCCTGGGCCTGGGCCTGAGCCTGGGGCCCTGGGCCACTGGGCCCTGGGCCGCCAGGCCACTGGGCCTTCAGGCCACTGAGCCACTGGGCCAGGCCTGGGCCCTGGGCCTGCACAGGCCTAGGCCACTGAGCCTAGGCCTGAGCCTGAGCCCTGGGCCCTGGGCCTAGGCTGCCCAAACCTCGGCCTGAGCCACTGAGCCTAGGCCCCTAGGCCCTGGGCCCTGAGCCTAGGCCTAGGCCTGGGCCCTGGGTGCAGGCCTAGGCCCTAGGCCCTGGGGCCCTAGGCCACTGAGCCACTGAGCCTGGGCCTGGGCCTGGGCCTGGGCCACTGAGCCACCAGGCCCTGGGCCACAGGCCCTAGGCCCTGGGTACAGGCCTGGGCTGCTGGGCCTGGGCCTGGGCCTGGCCTGGGCCTGGGCCACTGGGCCACTGAGCCTAGGCCTGGGCCCTGGGCCTGCAAGCCTGGGCCTAGGCACCTGACCTGGGCGCTGGAGCTACTGGGCCCACAGGCCACTGAGCCTGGGGCCCTAGGCCCTGGGCCACTGGGCCCTGGGCCACAGGCCTGGGCCTGGCCTGGGCCTGATGCTGGGCCACTGGGCCCCCAGGCCCTGGGCCCTGGGCCTAGGCCTGGGCCTGGGCTGCACAGGCCCTGGGCCCTGGGCCCTGGAGGCCTGGGCCCTGGGCCTAGGCCCTGGCTCCTGGGCTACTGGGCCTGGGCCACTGAGGCCTGGCCCTGAGGCCTGGGCCTGGGCCTCAGGCTACTAAGCCCTAGGCCTGGGCCTGTAAACCATGGAGCCACAGGCCTGGGCCTGGGCCTGGGCCTGGGCCTCAGGCCTGGGCCACAGGCCTGGGCCTGAGCCACTGGTACTGGCTCAGGCCTGAGCCCACTGAGGCCTAAGGCTGGGCCCTGGGCCTGGGCCCTGGGCCTAGGCCACTGGTACTGGTACTGGGCCTGGGCCTGGGCCCTGGCCTGGGCCTAGGCCTAGGCCACCCGACCACTAAACTAAACCCCTAAGCCTAGACACTGAAGCCCTGGGCCCTAGGCCTAGGCCTGGGCCCTGGGCCCTGAGCCACAGGCCTAGGCCTGGGCCTAGGCCCTGGGCCTGGCCTAGGCCTGGGCCTGGGCCACAGGCCTGGGCCCTGGGCCTGGGCCCTGGGCCCTGAGCCTGGGCCCTAGGCCCTGGGCCTGAGCCTAGGCCTGGGGCCTCAGACCTGGGCCACTGAGCCACTGAGCCTAGGCCACTGAGCCTGGGCCCACTGAGCCTGGGCCTGGGCCTAAGGCCTGGGCCTGGGCCTGGCCTGGGCCCTGGGCCTGGGCCTGGGCCCTGGGCCCTGGGCCTGGGCCCTGGGCCTGGGCCACAGGCCACTGGGCCACTGAGCCTGGGCCCTGTTTGGGGCGCCACAAACTAGGCCTAGGCCCTGAGCCTGGGCCTGAGCCCTGAGCCCCTGGGCTCCTGGGCCTAGGCCCTGGGCCTAGGCCACTGGGTGGCTGAGCCTGGCTAAGCCTGGGCGGCCTCGCCTGGGCCTGGGCCTGGGCCCTGGGCCTAGGCCTGGGCCCTGGGCCACAGGCCTGGGCCTGGGCCTGGGCCTGGGCCACTGGGCCTGGGCCTCAGGCCCTGGGCCTGGGCCTGGGCTCCTGGGCCCTGGGCTGGGCCCTGGGCCTGGGCCTGGGCCTGGGCCACTGAGCCTGGGCCTGGGCCTGGGCCTGGGCCTGGGCCTGGGCCTGAGCCTGGGCCAGGCCTGGGCCTGGCTGCAGGCCTGACACACAGGCCTGGGCCTGGGCCCTGGGCCACCAGGCCTAGGCCACAGGCTGGGCCTGGGCCTGGGCCTGGGCACTGGGCCTGGGCCACTGGGCCACTGAGCCTGGGCCTGGGCCACAGGCCCTGGGCCCTGGGCCTGGGCCCTGGGCCTGGGCCCTGGGCCCTGGGCCTGGGCCCTGGGCCTGGGCCCTGGGCCTCAGCCTGGGGCCTGGGCCTGGGCCTGGGCCCTGGGCCCTGGGCCTGGGCCTGAGGCCTGGGCCCTGGGCCCTGGGCCTGAATACACTGAGCCTAGGCCTGGGCCTGGGCCTGGGCCTGGGCCCTGGGCTCAGGCCACAGGCCTGAGCCTGGGCCACAGGCCTGGGCCACTGGGCCTGGGCCTGAGCCTGGGCCTGGGCCTGGGCCCTAGGCCCTGGGCCTGGGCCCAGGCCTGGGGCCCTGGGCCACTGAGCCTGGGCCTGGGCCTGGGCCTAGGCCTGGGCCTGAATTTTTCGACCTGGGCCTGGGCCTGGGCCTGGGCTGGGCCCCTAGGCCTGGGCCTGGGCCTGGGCCTGGGTGCTGGGCCTGGGCCTGGGCCCCTGGAGCCTATGGGCACCTGGGCCTGGGCCCTGGGCCCTGGGCCCTGGGCCACTAGGCCCTGGAGCCCTGGGCCCTGGGCCTGGGCCTGGGCCTAGGCCCTGGGCCCTGGTGCAGGCCCTGGGCCCTGGGCCTAGGCCTGAGCCCTGGGCCCTGGGCCTGGGCCTGGGCCTGGGCCTGGGCCTGGGCCTGGGCCTGGGCCTGGGCCTGGGCCCACTGGGCCTCTGGGCCTGAGCCACGGGCCACTGAGGCCTGGGCCTGGGCCTGGTGCAGGCCTGGGCCCACTGAGCCACTGGGGCCTGAGCCTGAGCCCTGAGCCCTAGGCCTGGGCCTGGGCCCTGGGCCCTGGGCCTGGGCCCTGTTTGGGCCTGGGCCTGAGCCACCAGGCCTGAGCCCTGGGCCTGGGCCTGGGCCTGGGCCCTGACCACAGGCCCTGGGCCCTGGGCCCTGGAGGCCTGGGCCTGGGCCTGGGCCCTGGGCCTGGGCCTGGGCCTGGGCAGCCTGGGCCTGGGCCTGGGCCTGGGCCCTGGGCCTGGTACAGGCCCTGGGCCCACTGGGCCTGGGCCCTGGGCCACTGGGCCTGGGCCTGGAGGCCTGGGCCCTGGGCCTGGGCCCTGGGCTGGGCCTGGGTACTGGGCCTGGGCCTGGGCCACTGACCTGGGCCACTGAGCCACCCAGGCCCTGGCCTGGGCCTGGGCTGCTGGGCCTGGCTGCAGGCCTGATACTAAACTGACCCTGGAGGCCTGGGCCCTGGGCCTGCAAACCACAGGCCTGGGCCTGGGCCCTGGGCCTGGGCCTGGGCCTGGGCCTGGGCCTGGGCCACTGGGCCACTGGTGGCCTGGGCCTGGGCCTGGGCCTGGGCCCTGGGCGGCCTGGGCCCTGGGCCTGGGCCCTGGGCCACTGGGCCTGGGCCACTCAGGCCCTGGGTGCAGGCCCTGGGCCTGGGCCTGGGCCTGAATGCTGGGCCTGAGCCTCGGCCTGGGCCTGGGCCTGGGCCTGGGCCTGGGCCTAAGGCCTGGGCCTGGGCCCTGGCCCTGGGGCGCTCAAGGGCCTGGGACCTGCAGACCGCTGGCCTCGGCCTCAGCTGATAGCCTAGGCCTGGGCCTGAGCTGCTCAGGCCTGGGCAGGCCTGTCGTGCTGAGCCTGGGCCTGAGCCTATAAGCCCTGCAGGCACTGGCCTGGGCCTGAGCCTAGGCCCTGAGCCCTGGGCCTAGGCCTAGGCCTGGGCCACTGAGCCTGAGCCACACAGGCCCCTGGGCCTGGGCCCTAGGCCTGAGCCTGGGCCTGGCCCAGGCCACAGGCCTGGGCCTAGGCCTGGGCCACTGGGCCCTGGGCCTGGGCCACAGGCCTAGGCCCTGAGCTACTGACCCTAGGCCTAGGCCTGTGACGCCTGGGCCTGGGCCCTGGGCCTGGGCCCTGGGCCTGAGCCTGGGCCTGGGCCTGGGCCTGGGCCCTGGGCCTGGGCTGGAGCCACCTGGGCCTGGGCCTGGGCGGGGCCTGGGCCTGGGCCCTGGCCTGGGCCTGGGCCTGGGCCCAGGCCTGGGCCACAGGCCCAGGCCTGGGCCACAGGCCTGGGCCCTGGGCCTGGGCCTGGGCCCTAGGCCTGGAGGCCTGGGTACAGGCCCTGGGCCCTGGGCCTGGGCCTGGGCCTGGGCCTCGGCCTGGGGCCACTGAGCCTGGGCCTGGGCCACTGAGGCCTCGGCCACAGGCCCTGGGCCACAGGCCACAGGCCTGGGCCTGGCCACAAACTAGGCCTAGGCCACAGGCCTGGGCCTAAGGCCACAGGCCTGGGCCCTGGGCCACTGAGCCTAGGCCTGGGCCCTGAATACTGGGCCACTGAATACTGGGCCTGGGCCCTGGGCCTGAGCCTGGGCCACTGAATGCCTGGGCCTGGGTACTGGGCCACTGGCCTGGGGCCTGGGCCTGGGTGGCCCTGGGCCTAAGGCTGGGCCTGGGGCCTGGGCCTGGGCCCTTAAACTAGGCCACTGAGCCTGCAGGCCACTGAGCCCTGGAGCCTGGGCCACTGAGGCCACTGGGTACGGACTAGGAGCCTGCAAACAAGCCTGGGCCCACTGAGCCCTGGGCCACTGGGCCCACTGAGGCCCTGGGCCACTGGGGCCTGGGCCTGGGCCCTGGGCCTGGGCCCTGGGCCCTGGGCCCTGGAGGCCTGGGCTGGGCCTGGGCCTGGGCTGGGCTGGGCTGGCCTGGGCCCCCAAACCTAGGCTCAGGCCCTGGGGCCTGGGCCACTGGGCCTGGGCCTGGCTCAGGCCTGGGCCACCCTGGGCCTGGGCCCACTGGCCTGGGCCCTGGGCCTAAGGCCCTGTGGGCCCTGAATGCTGAGCCTGGGCCTGGGCCTGGGCCTGGGCCCTGGAGGCCTGGGGCCTGGGCCTGGGCCTAGGCCTGGGCCACTGAGCCTGGGCCTGGAAGCCCGCTGGGCCACTGGGCCACTGGGCCACCACAGGCCCTCGTGCAGGCCTGGGCCTGGGCCTGGGCCACTGGGCCTGGGCCCTGGGCCTGGCTGCTGAGGCCACTGAATACCTGAATACTGAGCCTGAGCCACAGGCCTGAGCCTAGGCCCTAGGCCTAGATAGACACTGGGCCCTGGGCCTGAGCCCTGGGCCCACTGAGCCCAGGCCACTGAGCCCTAGGCCTGGGCCTGGGCCTGGGCCCTGGGGCCTGGGCCTGGGCCTGGGCCTGGGCCCTGGGCCTGGGCCTGGGCCTGGGCTGTGGCCTGAGCCCTGGGGCCTGGGCCTAAGGCCACTGGGCCTGAGCCCTGCGGCCACAGGCCTGCACAGGCCCTGGGCTGCAGGCCTGGGCCTGGGCCACTGGGCCTGGCCACTGAGCCACTGAGCCACACAGGCCTCCTGGGCCCTGGGCCTAGGCCCTGGGCCTGGGCCTAAGGCCCTGGAGCCTGGGCCACAAGCCTGACACTGGGCCACAGGCCACCAGGCCTGGGCCTGGGCCTGGCCACCAGGCCTAGGCCTGGGCCCTGGGCCTTGGCTGGG
